# Supplementary material for: Evidence for late Pleistocene origin of Astyanax mexicanus cavefish
Source: BMC Evol Biol. 2018 Apr 18;18:43. doi: 10.1186/s12862-018-1156-7 (PMC5905186; doi:10.1186/s12862-018-1156-7)
Supplement: Supplementary file 1 — Supplementary figures and tables. (PDF 1092 kb) [file 12862_2018_1156_MOESM1_ESM.pdf]

Supplementary figures

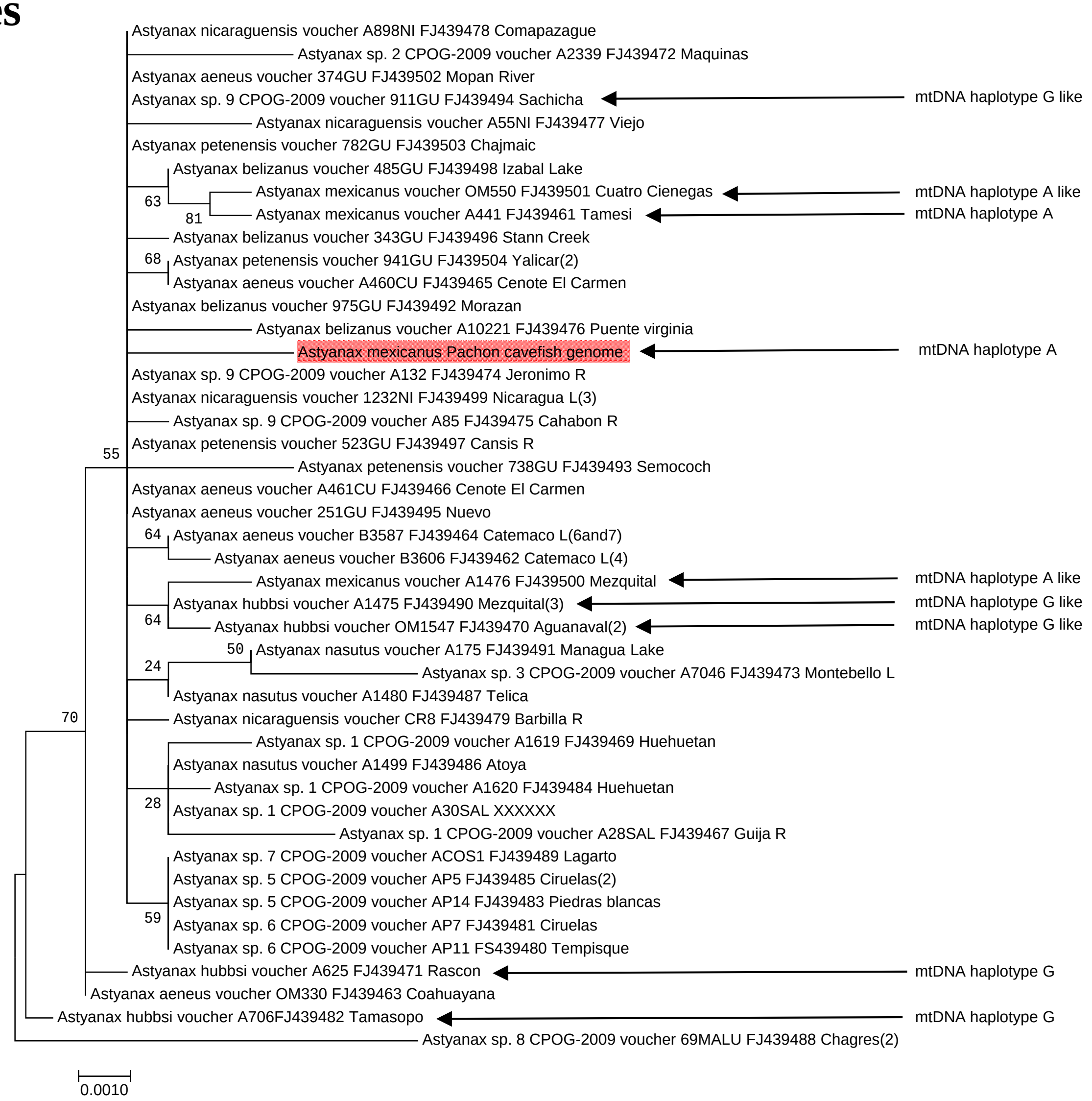

Figure S1: Rag1 phylogeny. The evolutionary tree was inferred by using the Maximum Likelihood method based on the Tamura-Nei model The analysis involved 45 nucleotide sequences All positions containing gaps and missing data were eliminated. There were a total of 1251 positions in the final dataset. Evolutionary analyses were conducted in MEGA5 [1]. All sequences belong to surface fish, excepted one Pachón cavefish sequence from the genome project and highlighted in red. Individuals with mtDNA belonging to A or G haplogroups are indicated as well as those with mtDNA haplotypes closely related to these haplogroups.

[1] Tamura K., Peterson D., Peterson N., Stecher G., Nei M., and Kumar S. (2011). MEGA5: Molecular Evolutionary Genetics Analysis using Maximum Likelihood, Evolutionary Distance, and Maximum Parsimony Methods. Molecular Biology and Evolution (10):2731-9.

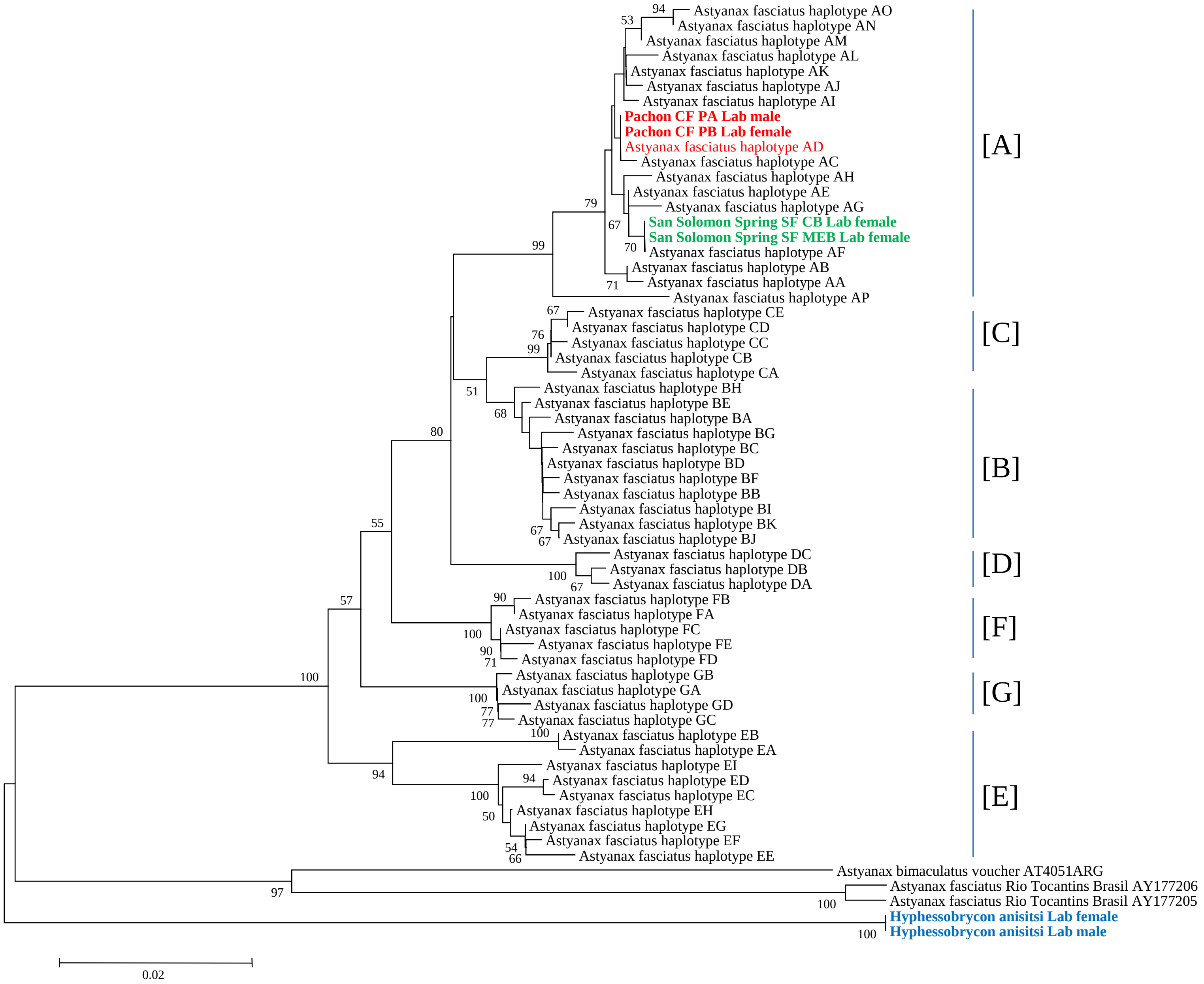

Figure S2: Neighbor joining phylogeny of *Astyanax mexicanus* cyt b haplotypes (see Stecker et al. 2004 for more details). Seven haplogroups were identified [A] to [G]. Cavefish haplotypes belong to haplogroup [A] and [G]. In the Pachón cave, only the haplotype AD was found (red). The two San Solomon Spring surface fish are in green, the two *Hyphessobrycon anisitsi* fish are in blue. The names of the lab fish we sequenced are in bold.

Chica - Surface
O8 – S3

Sample size : 22 loci; 60 alleles/locus/population
Command line:
./IMa2 -iinfileO8S3_n60.u -ooutput_O8S3_n60_m10.out -b200000 -l100000 -d100 -m10 -q200 -t4 -c1 -p23567 -r25 -u1 -hfg -hn50 -ha0.999 -hb0.3

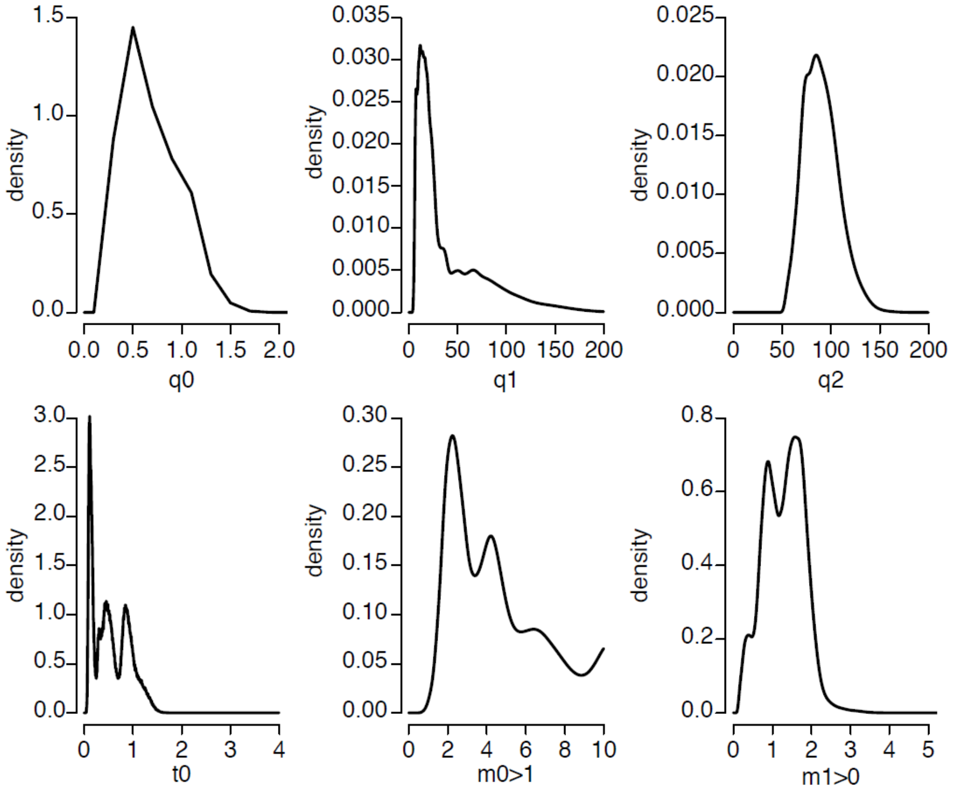

Estimation of demographic parameters with IMa2

	**HiPt**	**HPD 95%**
**q0 ($4N_eu$ Chica)**	0.500	0.300 – 2.500
**q1 ($4N_eu$ Surface)**	11.700	3.900 – 120.7
**q2 ($4N_eu$ Ancestral)**	84.900	57.500 – 125.300
**t0 (tu)**	0.114	0.005- 1.186
**m0>1 ($m_{0->1}/u$)**	2.245	1.275 – 9.995
**m1>0 ($m_{1->0}/u$)**	1.585	0.275 – 2.145
**N_e Chica**	250	150 – 1,250
**N_e Surface**	5,850	1,950 – 60,350
**N_e Ancestral**	42,450	28,750 – 62,650
**t**	798	30 – 8,302
**$m_{S->C}$**	11.2×10^{-4}	$6.4 – 50.0 \times 10^{-4}$
**$m_{C->S}$**	7.93×10^{-4}	$1.3 – 10.7 \times 10^{-4}$

We assumed a mutation rate (u) of 0.0005 and a mean generation time (g) of 3.5 years (2 years for surface fish and 5 years for cavefish). HiPt: the highest value in the histogram of the posterior marginal density. HPD 95%: the lower and upper bound of the estimated 95% highest posterior density (HPD) interval. Effective population size (N_e) Chica = q0 / 4u ; N_e surface = q1 / 4u ; N_e ancestral = q2 / 4u ; t (divergence time in years) = t0 x g / u ; $m_{S->C}$ (migration rate from surface to Chica) = m0>1 x u ; $m_{C->S}$ (migration rate from Chica to surface) = m1>0 x u. m0>1 and m1>0 are migration rate backward in time, whereas $m_{S->C}$ and $m_{C->S}$ are migration rate forward in time.

Figure S3:

Pachón - Chica
O1 – O8

Sample size : 22 loci; 60 alleles/locus/population
Command line:
./IMa2 -iinfileO1O8_n60.u -ooutputO1O8_n60_long.out -b10000 -l100000 -d100 -m5 -q200 -t4 -p2367 -r25 -u1 -hfg -hn50 -ha0.999 -hb0.3

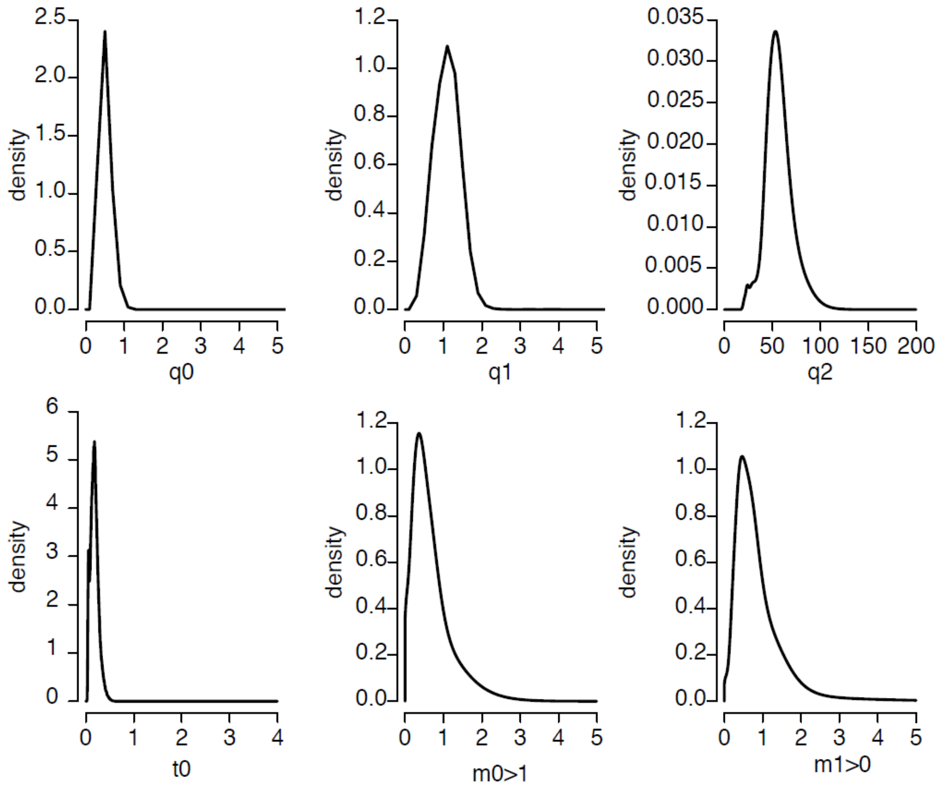

Estimation of demographic parameters with IMa2

	HiPt	HPD 95%
**q0 ($4N_eu$ Pachón)**	0.500	0.300 – 2.300
**q1 ($4N_eu$ Chica)**	1.100	0.300 – 3.100
**q2 ($4N_eu$ Ancestral)**	53.300	28.300 – 88.300
**t0 (tu)**	0.182	0.038 – 0.346
**m0>1 ($m_{0\text{->}1}/u$)**	0.686	0.000 – 1.748
**m1>0 ($m_{1\text{->}0}/u$)**	0.866	0.000 – 2.027
**N_e Pachón**	250	150 – 1150
**N_e Chica**	550	150 – 1,550
**N_e Ancestral**	26,650	14,150 – 44,150
**t**	1,274	266 – 2,422
**$m_{C\text{->}P}$**	3.4×10^{-4}	$0.0 – 8.7 \times 10^{-4}$
**$m_{P\text{->}C}$**	4.3×10^{-4}	$0.0 – 10.1 \times 10^{-4}$

We assumed a mutation rate (u) of 0.0005 and a mean generation time (g) of 5 years for cavefish. HiPt: the highest value in the histogram of the posterior marginal density. HPD 95%: the lower and upper bound of the estimated 95% highest posterior density (HPD) interval. Effective population size (N_e) Pachón = q0 / 4u ; N_e Chica = q1 / 4u ; N_e ancestral = q2 / 4u ; t (divergence time in years) = t0 x g / u ; $m_{C\text{->}P}$ (migration rate from Chica to Pachón) = m0>1 x u ; $m_{P\text{->}S}$ (migration rate from Pachón to Chica) = m1>0 x u. m0>1 and m1>0 are migration rate backward in time, whereas $m_{S\text{->}P}$ and $m_{P\text{->}S}$ are migration rate forward in time.

Figure S4:

Molino - Surface
N1 – S1

Sample size : 14 loci; 40 alleles/locus/population
Command line:
./IMa2 -iinfileN1S1_n40.u -ooutput_N1S1_n40_longrun.out -b200000 -l100000 -d100 -m20 -q200 -t4 -c1 -p23567 -r25 -u1 -hfg -hn50 -ha0.999 -hb0.3

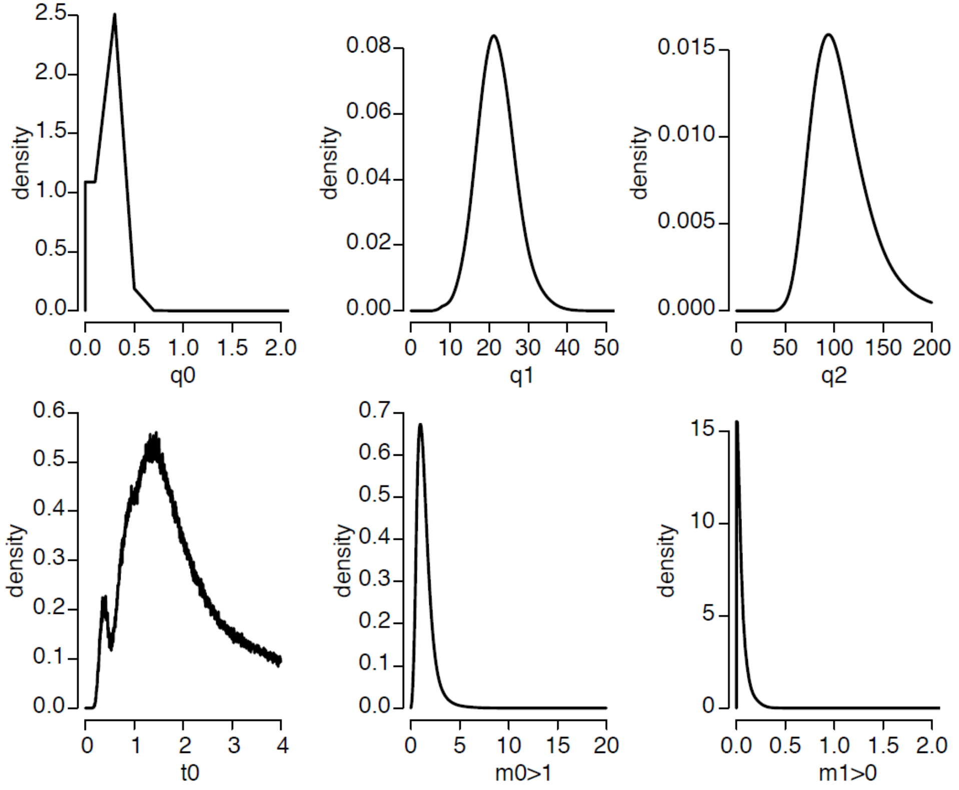

Estimation of demographic parameters with IMa2

	**HiPt**	**HPD 95%**
**q0 (4N_eu Molino)**	0.300	0.000 – 1.900
**q1 (4N_eu Surface)**	21.100	12.100 – 31.900
**q2 (4N_eu Ancestral)**	94.100	57.900 – 163.100
**t0 (tu)**	1.450	0.286 – 3.570
**m0>1 ($m_{0->1}$/u)**	0.970	0.190 – 3.030
**m1>0 ($m_{1->0}$/u)**	0.010	0.000 – 0.250
**N_e Molino**	150	0 – 950
**N_e Surface**	10,550	6,050 – 15,950
**N_e Ancestral**	47,050	28,950 – 81,550
**t**	10,150	2,002 – 24,990
**$m_{S->M}$**	4.9 x 10^{-4}	1.0 – 15.2 x 10^{-4}
**$m_{M->S}$**	5. 0 x 10^{-6}	0.0 – 1.3 x 10^{-4}

We assumed a mutation rate (u) of 0.0005 and a mean generation time (g) of 3.5 years (2 years for surface fish and 5 years for cavefish). HiPt: the highest value in the histogram of the posterior marginal density. HPD 95%: the lower and upper bound of the estimated 95% highest posterior density (HPD) interval. Effective population size (N_e) Molino = q0 / 4u ; N_e surface = q1 / 4u ; N_e ancestral = q2 / 4u ; t (divergence time in years) = t0 x g / u ; $m_{S->M}$ (migration rate from surface to Molino) = m0>1 x u ; $m_{M->S}$ (migration rate from Molino to surface) = m1>0 x u. m0>1 and m1>0 are migration rate backward in time, whereas $m_{S->M}$ and $m_{M->S}$ are migration rate forward in time.

Figure S5:

Caballo Moro - Surface
N2 – S2

Sample size : 18 loci; 40 alleles/locus/population
Command line:
/IMa2 -iinfileN2S2_n40.u -ooutput_N2S2_n40_m5_longrun.out -b200000 -l100000 -d100 -m5 -q200 -t4 -c1 -p23567 -r25 -u1 -hfg -hn50 -ha0.999 -hb0.3

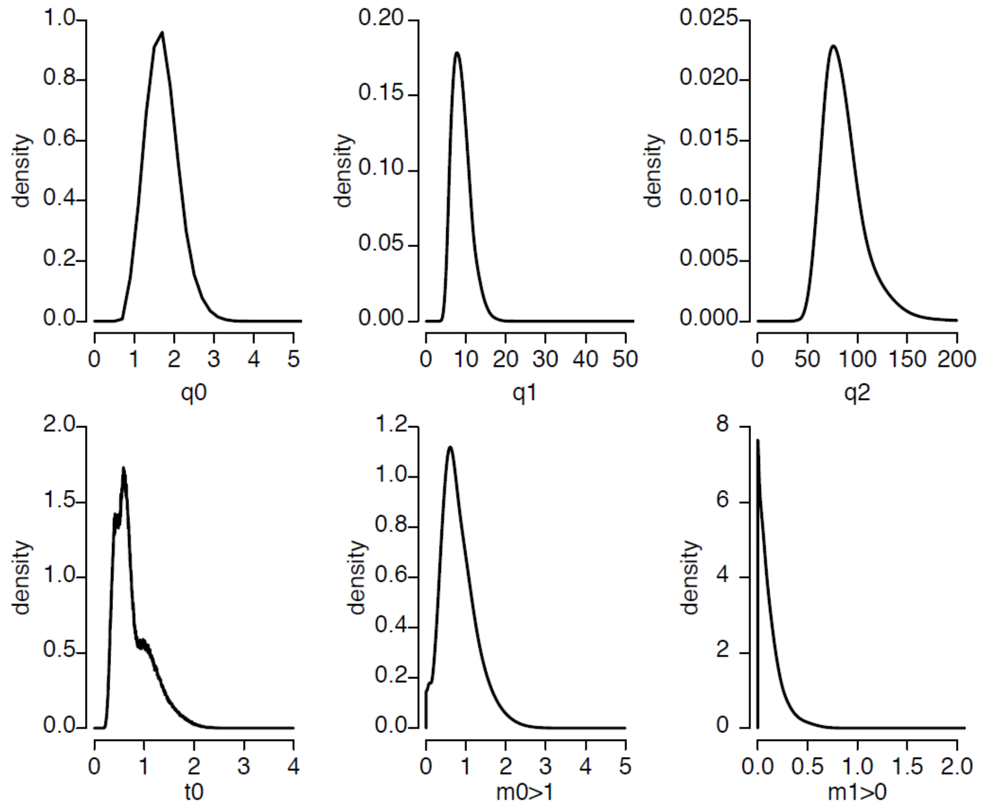

Estimation of demographic parameters with IMa2

	HiPt	HPD 95%
**q0 (4N_eu Caballo M.)**	1.700	0.700 – 3.700
**q1 (4N_eu Surface)**	7.700	4.300 – 13.900
**q2 (4N_eu Ancestral)**	75.900	50.500- 130.700
**t0 (tu)**	0.582	0.262 – 1.518
**m0>1 ($m_{0\text{->}1}$/u)**	0.603	0.008 – 1.657
**m1>0 ($m_{1\text{->}0}$/u)**	0.003	0.000 – 0.363
**N_e Caballo Moro**	850	350 – 1,850
**N_e Surface**	3,850	2,150 – 6,950
**N_e Ancestral**	37,950	25,250 – 65,350
**t**	4,074	1,834 – 10,626
**$m_{S\text{->}C}$**	3.0 x 10^{-4}	0.0 – 8.3 x 10^{-4}
**$m_{C\text{->}S}$**	1.5 x 10^{-6}	0.0 – 1.8 x 10^{-4}

We assumed a mutation rate (u) of 0.0005 and a mean generation time (g) of 3.5 years (2 years for surface fish and 5 years for cavefish). HiPt: the highest value in the histogram of the posterior marginal density. HPD 95%: the lower and upper bound of the estimated 95% highest posterior density (HPD) interval. Effective population size (N_e) Caballo Moro = q0 / 4u ; N_e surface = q1 / 4u ; N_e ancestral = q2 / 4u ; t (divergence time in years) = t0 x g / u ; $m_{S\text{->}C}$ (migration rate from surface to Caballo Moro) = m0>1 x u ; $m_{C\text{->}S}$ (migration rate from Caballo Moro to surface) = m1>0 x u. m0>1 and m1>0 are migration rate backward in time, whereas $m_{S\text{->}C}$ and $m_{C\text{->}S}$ are migration rate forward in time.

Figure S6:

Subterráneo - Surface
N3 – S4

Sample size : 21 loci; 40 alleles/locus/population
Command line:
/IMa2 -iinfileN3S4_n40.u -ooutput_N3S4_n40_m20_longrun.out -b200000 -l100000 -d100 -m20 -q200 -t4 -c1 -p23567 -r25 -u1 -hfg -hn50 -ha0.999 -hb0.3

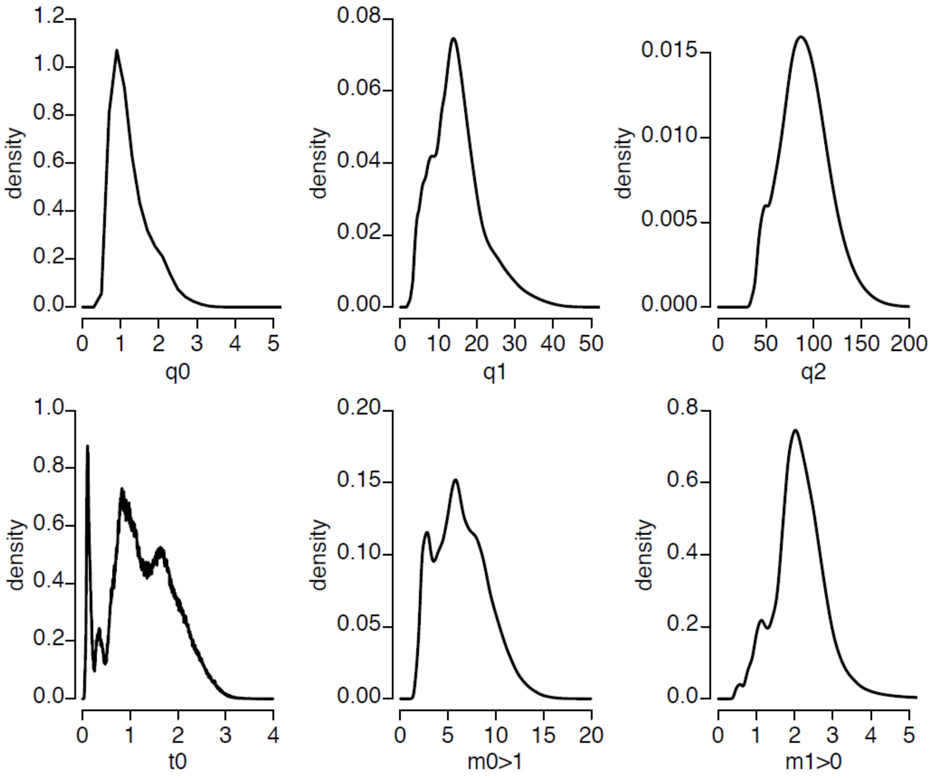

Estimation of demographic parameters with IMa2

	**HiPt**	**HPD 95%**
**q0 (4N_eu Subterráneo)**	0.900	0.500 – 3.300
**q1 (4N_eu Surface)**	13.900	3.100 – 28.700
**q2 (4N_eu Ancestral)**	86.700	40.700 – 138.300
**t0 (tu)**	0.110	0.058 – 2.462
**m0>1 ($m_{0->1}$/u)**	5.790	1.790 – 11.430
**m1>0 ($m_{1->0}$/u)**	2.030	0.750 – 3.430
**N_e Subterráneo**	450	250 – 1,650
**N_e Surface**	6,950	1,550 – 14,350
**N_e ancestral**	43,350	20,350 – 69,150
**t**	770	406 – 17,234
**$m_{S->C}$**	29.0 x 10^{-4}	9.0 – 57.2 x 10^{-4}
**$m_{C->S}$**	10.2 x 10^{-4}	3.8 – 17.2 x 10^{-4}

We assumed a mutation rate (u) of 0.0005 and a mean generation time (g) of 3.5 years (2 years for surface fish and 5 years for cavefish). HiPt: the highest value in the histogram of the posterior marginal density. HPD 95%: the lower and upper bound of the estimated 95% highest posterior density (HPD) interval. Effective population size (N_e) Subterraneo = q0 / 4u ; N_e surface = q1 / 4u ; N_e ancestral = q2 / 4u ; t (divergence time in years) = t0 x g / u ; $m_{S->C}$ (migration rate from surface to Subterraneo) = m0>1 x u ; $m_{C->S}$ (migration rate from Subterraneo to surface) = m1>0 x u. m0>1 and m1>0 are migration rate backward in time, whereas $m_{S->C}$ and $m_{C->S}$ are migration rate forward in time.

Figure S7:

Curva - Surface
O6 – S3

Sample size : 14 loci; 20 alleles/locus/population
Command line:
/IMa2 –iinfileO6S3_1.u -ooutput_06S3_1_m20_longrun.out -b200000 -l100000 -d100 –m20 -q200 -t4 -c1 -p23567 -r25 -u1 -hfg -hn50 -ha0.999 -hb0.3

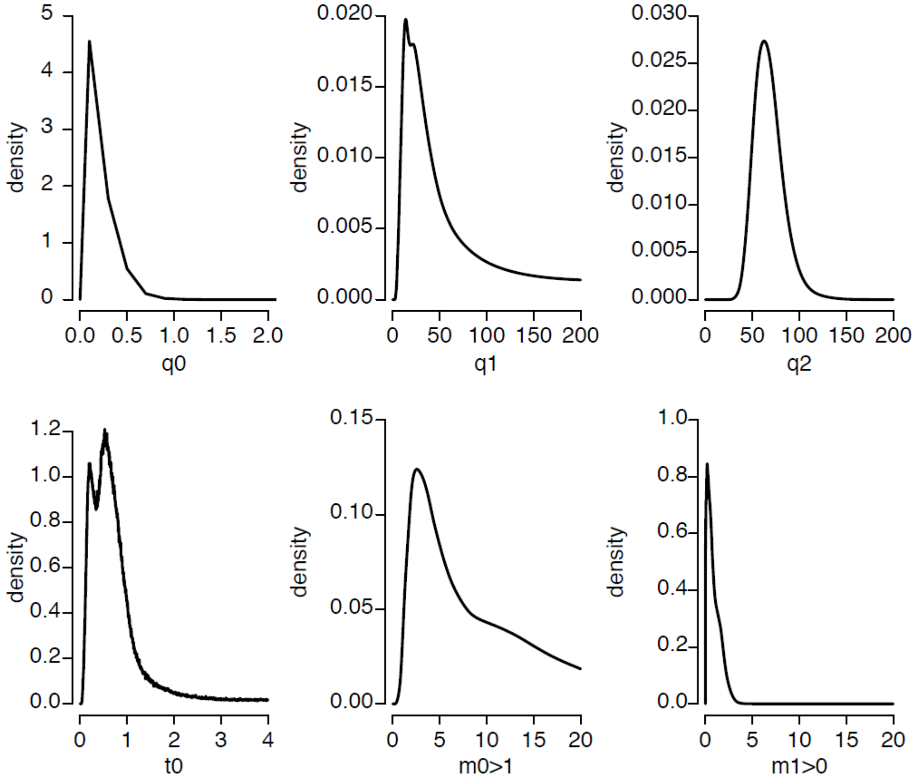

Estimation of demographic parameters with IMa2

	**HiPt**	**HPD 95%**
**q0 (4N_eu Curva)**	0.100	0.000 – 1.900
**q1 (4N_eu Surface)**	13.900	4.300 – 166.300
**q2 (4N_eu Ancestral)**	62.500	39.700 – 99.300
**t0 (tu)**	0,526	0.050 – 1.982
**m0>1 ($m_{0\text{->}1}$/u)**	2.570	0.930 – 17.790
**m1>0 ($m_{1\text{->}0}$/u)**	0.190	0.000 – 2.230
**N_e Curva**	50	0 – 950
**N_e Surface**	6,950	2,150 – 83,150
**N_e ancestral**	31,250	19,850 – 49,650
**t**	3,682	350 – 13,874
**$m_{S\text{->}C}$**	12.8 x 10^{-4}	4.7 – 89.0 x 10^{-4}
**$m_{C\text{->}S}$**	1.0 x 10^{-4}	0.0 – 11.2 x 10^{-4}

We assumed a mutation rate (u) of 0.0005 and a mean generation time (g) of 3.5 years (2 years for surface fish and 5 years for cavefish). HiPt: the highest value in the histogram of the posterior marginal density. HPD 95%: the lower and upper bound of the estimated 95% highest posterior density (HPD) interval. Effective population size (N_e) Curva = q0 / 4u ; N_e surface = q1 / 4u ; N_e ancestral = q2 / 4u ; t (divergence time in years) = t0 x g / u ; $m_{S\text{->}C}$(migration rate from surface to Curva) = m0>1 x u ; $m_{C\text{->}S}$ (migration rate from Curva to surface) = m1>0 x u. m0>1 and m1>0 are migration rate backward in time, whereas $m_{S\text{->}C}$ and $m_{C\text{->}S}$ are migration rate forward in time.

Figure S8:

Pachón - Curva
O1 - O6

Sample size : 14 loci; 20 alleles/locus/population
Command line:
/IMa2 -iinfileO1O6_0.u -ooutput_O1O6_0_m10_longrun.out -b200000 -l100000 -d100 -m10 -q200 -t4 -c1 -p23567 -r25 -u1 -hfg -hn50 -ha0.999 -hb0.3

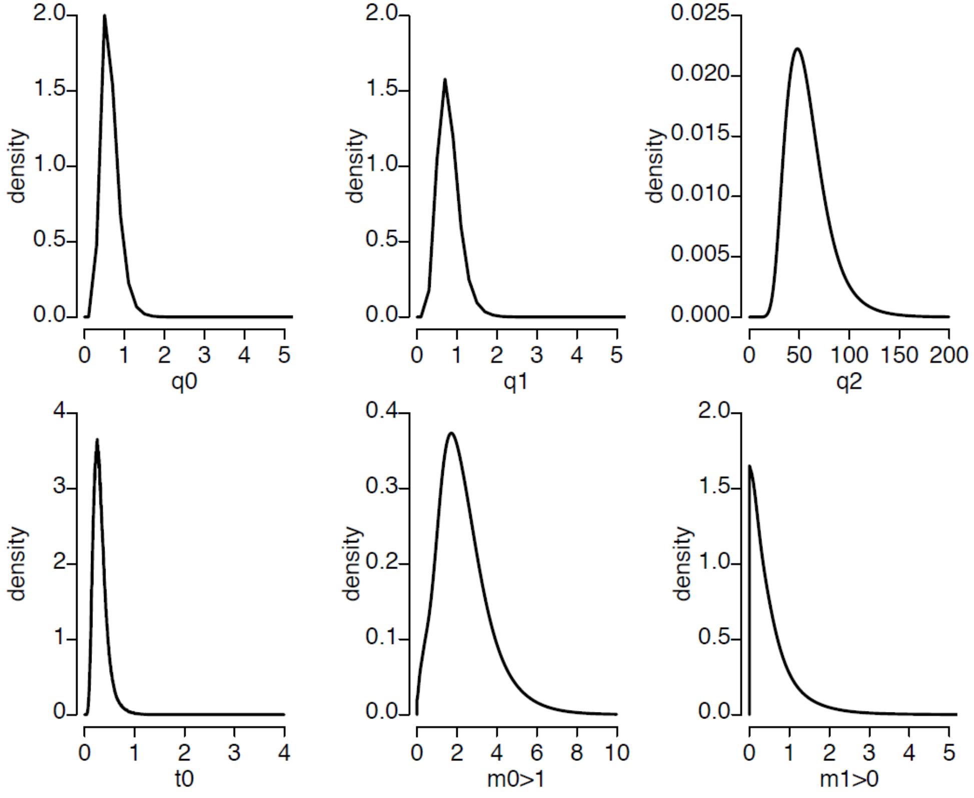

Estimation of demographic parameters with IMa2

	**HiPt**	**HPD 95%**
**q0 ($4N_eu$ Pachón)**	0.500	0.300 – 2.500
**q1 ($4N_eu$ Curva)**	0.700	0.300 – 2.700
**q2 ($4N_eu$ Ancestral)**	48.100	23.900 – 101.500
**t0 (tu)**	0.254	0.086 – 0.598
**m0>1 ($m_{0->1}/u$)**	1.715	0.105 – 4.905
**m1>0 ($m_{1->0}/u$)**	0.005	0.000 – 1.705
**N_e Pachón**	250	150 – 1,250
**N_e Curva**	350	150 – 1,350
**N_e ancestral**	24,050	11,950 – 50,750
**t**	2,540	860 – 5,980
**$m_{C->P}$**	8.58×10^{-4}	$0.53 – 24,52 \times 10^{-4}$
**$m_{P->C}$**	2.50×10^{-6}	$0.00 – 8.53 \times 10^{-4}$

We assumed a mutation rate (u) of 0.0005 and a mean generation time (g) of 5 years. HiPt: the highest value in the histogram of the posterior marginal density. HPD 95%: the lower and upper bound of the estimated 95% highest posterior density (HPD) interval. Effective population size (N_e) Pachón = q0 / 4u ; N_e Curva = q1 / 4u ; N_e ancestral = q2 / 4u ; t (divergence time in years) = t0 x g / u ; $m_{C>P}$ (migration rate from Curva to Pachón) = m0>1 x u ; $m_{P->C}$ (migration rate from Pachón to Curva) = m1>0 x u. m0>1 and m1>0 are migration rate backward in time, whereas $m_{C->P}$ and $m_{P->C}$ are migration rate forward in time.

Figure S9:

	Lab populations collected in the wild in 2000		Purchased in 2012
	A. mexicanus Texas surface fish	*A. mexicanus* Pachón cavefish	*H. anisitsi*

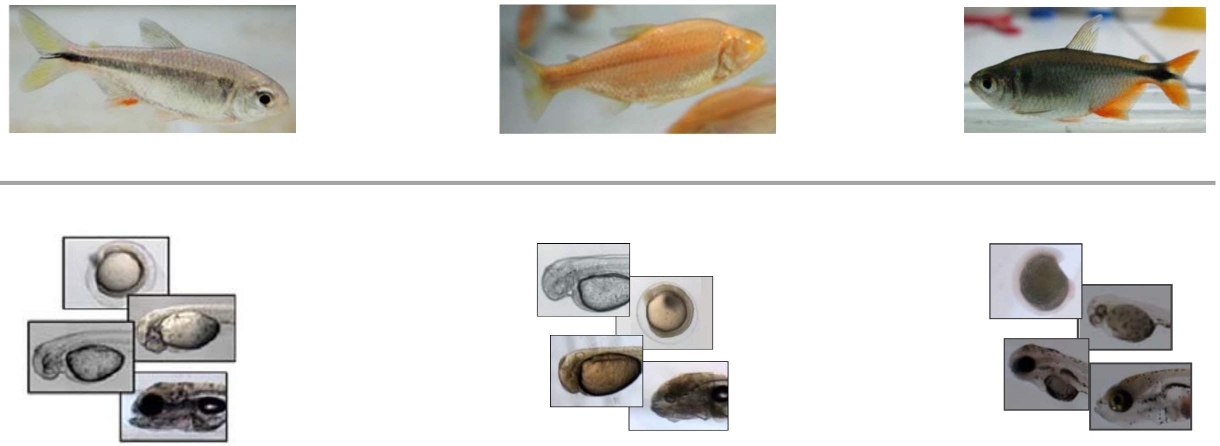

Fifty to 200 embryos/larvae from several independent spawn events and several developmental stages (6 hpf to 15 dpf) were pooled.

Number of RNA sequences:			
Sanger	1.10^5	1.10^5	
454	1.10^6	1.10^6	
Illumina	250.10^6	250.10^6	250.10^6

Figure S10: Population sampling and RNA sequencing

Effect of “sequencing depth” threshold, *i.e.* the minimal number of reads in each population.
Blast e-value = 10^{-5}
Minor allele frequency : 0.05
Window : 50

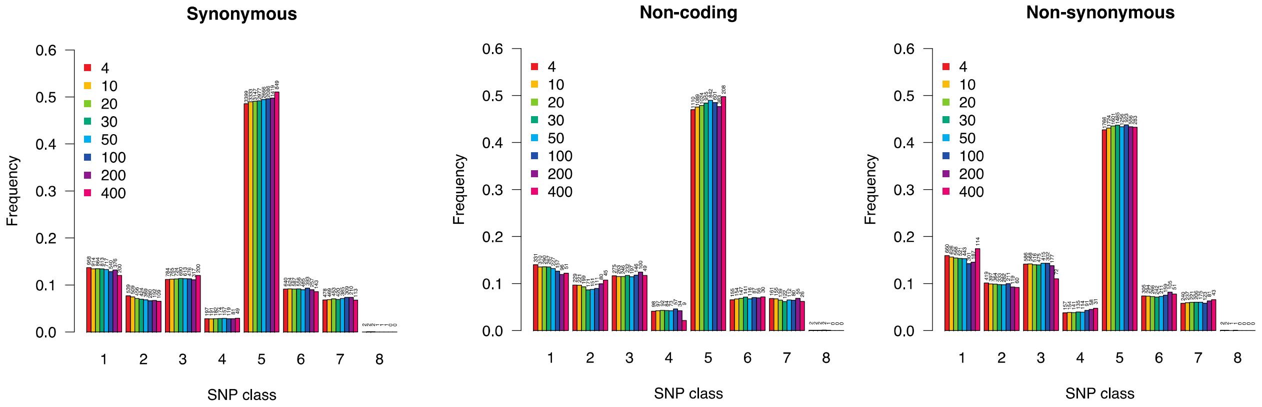

Effect of “sequence conservation” threshold, *i.e.* the blast best hit e-value between the contig and the zebrafish sequence.
Sequencing depth = 100
Minor allele frequency : 0.05
Window : 50

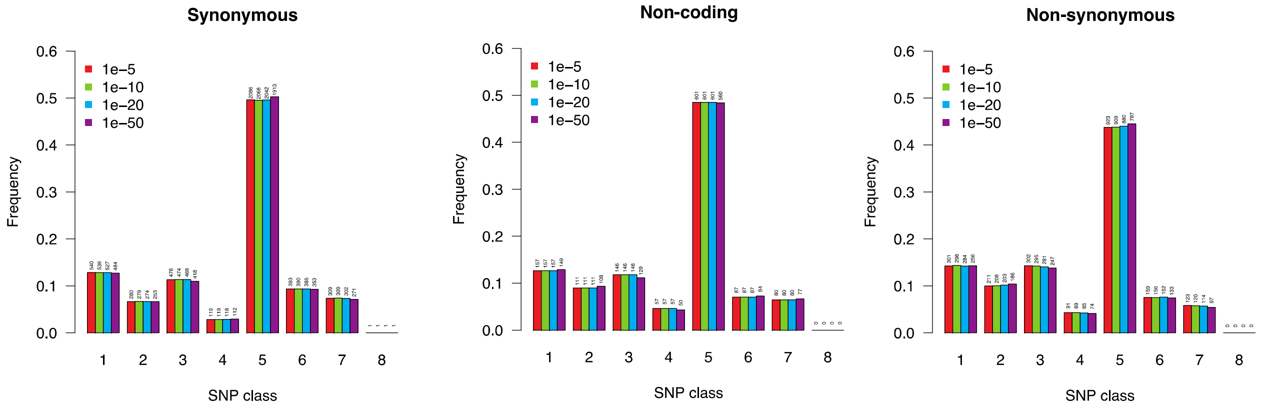

Effect of “SNP isolation” threshold, *i.e*. no other SNP in a window of a given size
Blast e-value = 10^{-5}
Sequencing depth = 100
Minor allele frequency : 0.05

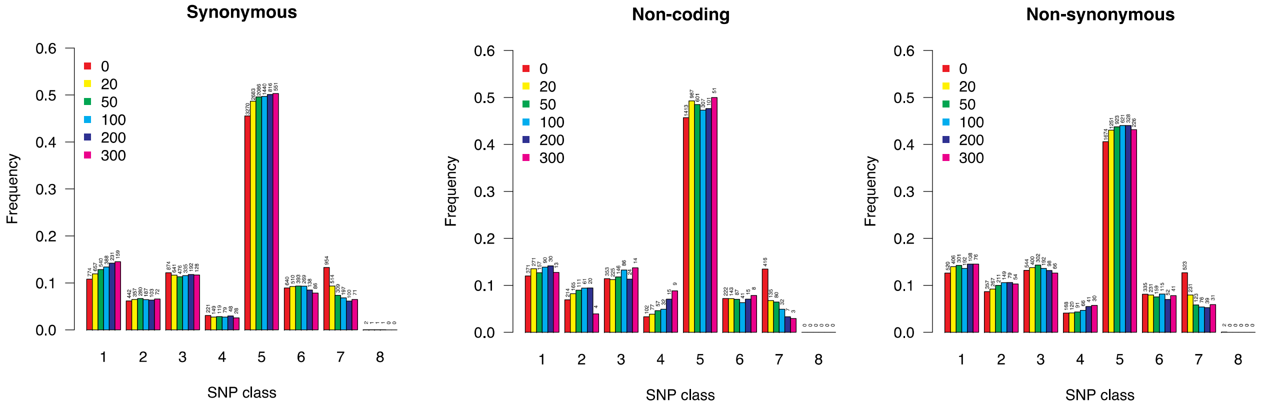

Figure S11: The effect of different thresholds on the estimation of SNP class frequencies

Supplementary tables

Table S1

number of alleles genotyped / locus / locality

			A13e5	Hc5	Vf9	Ra8	Xa3	Ib1	A13f8	A14d12	A19c8	A14d8	M_f4	Vf3	A7c5
surface	Guatemala	**S1**	56	64	56	70	70	72	40	72	60	72	44	82	80
surface	Guatemala	**S2**	32	54	24	62	68	52	64	74	36	48	24	74	60
surface	El Abra	**S3**	142	166	126	170	180	176	108	212	154	178	102	228	210
surface	Micos	**S4**	44	52	28	44	34	58	34	58	40	42	24	56	56
cave	Pachón	**O1**	68	82	0	74	86	66	88	88	30	78	38	84	80
cave	Yerbaniz	**O2**	16	20	4	22	22	16	22	22	16	20	18	24	20
cave	Japones	**O3**	16	18	4	14	16	14	18	20	14	20	18	18	20
cave	Arroyo	**O4**	14	22	0	14	20	16	22	24	8	16	10	22	22
cave	Tinaja	**O5**	8	8	0	8	8	4	8	8	4	4	4	8	8
cave	Curva	**O6**	8	20	0	14	22	6	26	26	0	16	26	18	18
cave	Toro	**O7**	4	6	0	4	6	6	6	6	4	4	4	6	6
cave	Chica	**O8**	206	210	206	220	206	180	228	162	196	198	142	218	220
cave	Molino	**N1**	34	42	26	32	42	36	44	44	38	32	34	44	44
cave	Caballo Moro	**N2**	32	44	34	44	44	26	48	52	46	42	42	50	50
cave	Subterráneo	**N3**	78	122	106	124	124	52	140	138	54	136	0	134	144

			A5f9	A2a7	a4g11	a6f1	wd11	a12f10	Lb9	wc12	a6h6	wf6	A10b5	vc10	a8g5
surface	Guatemala	**S1**	84	82	70	68	80	78	82	78	82	74	70	28	58
surface	Guatemala	**S2**	66	76	68	58	60	56	56	58	46	54	30	32	42
surface	El Abra	**S3**	224	226	202	158	198	208	188	210	194	202	184	66	128
surface	Micos	**S4**	58	58	44	54	52	54	48	54	44	56	40	32	40
cave	Pachón	**O1**	86	84	86	80	70	82	74	80	54	84	76	74	84
cave	Yerbaniz	**O2**	22	22	22	22	22	16	16	16	16	20	22	16	16
cave	Japones	**O3**	18	20	10	12	12	16	16	16	16	16	10	14	18
cave	Arroyo	**O4**	24	24	16	18	18	20	22	20	22	18	14	22	24
cave	Tinaja	**O5**	8	8	8	8	8	6	8	8	8	8	8	8	8
cave	Curva	**O6**	26	26	26	22	26	22	26	22	26	18	18	8	14
cave	Toro	**O7**	6	6	6	6	6	6	6	6	6	4	2	0	2
cave	Chica	**O8**	234	216	212	216	208	196	216	184	216	232	234	224	232
cave	Molino	**N1**	44	44	22	30	36	38	42	42	42	44	44	42	42
cave	Caballo Moro	**N2**	44	50	40	38	44	50	52	52	52	50	50	52	52
cave	Subterráneo	**N3**	138	142	130	128	108	140	136	140	136	140	128	130	140

Table S2a:
Classification of polymorphisms in *Astyanax mexicanus* Texas surface *vs* the Pachón cave populations

	Class	Synonymous		Non-coding		Non-synonymous	
		n	*%*	n	*%*	n	*%*
**Ancestral fixed SF, derived fixed CF**	(1)	612	*14.6*	180	*14.6*	343	*16.3*
**Ancestral fixed CF, derived fixed SF**	(2)	351	*8.4*	144	*11.7*	246	*11.7*
**Polymorphism CF, ancestral fixed SF**	(3)	434	*10.3*	130	*10.6*	274	*13.0*
**Polymorphism CF, derived fixed SF**	(4)	110	*2.6*	49	*4.0*	81	*3.8*
**Polymorphism SF, ancestral fixed CF**	(5)	2,062	*49.1*	588	*47.8*	911	*43.3*
**Polymorphism SF, derived fixed CF**	(6)	391	*9.3*	84	*6.4*	155	*7.4*
**Shared polymorphism**	(7)	240	*5.7*	55	*4.5*	96	*4.6*
**Divergent**	(8)	0	*0.0*	0	*0.0*	0	*0.0*
**Total**		4,200	*100.0*	1,230	*100.0*	2,106	*100.0*
**Polymorphism SF**	(5+6+7)	2,693		727		1,162	
**Polymorphism CF**	(3+4+7)	784		234		451	
**Ratio SF/CF**		3.43		3.11		2.58	
**Derived and fixed SF**	(2+4)	461		193		327	
**Derived and fixed CF**	(1+6)	1,003		264		498	
**Ratio CF/SF**		2.18		1.37		1.52	

Thresholds: 100; MAF > 10%; Score Blast < 10^{-5}; interval > 50bp (see materials and methods for threshold definitions.)
CF: Cavefish; SF: Surface fish; numbers in brackets are class identifiers described in fig. 4.

Table S2b:
Classification of polymorphisms in *Astyanax mexicanus* Texas surface *vs* Pachón cave populations

	Class	Synonymous n	Synonymous %	Non-coding n	Non-coding %	Non-synonymous n	Non-synonymous %
**Ancestral fixed SF, derived fixed CF**	(1)	462	*11.0*	123	*9.9*	247	*11.6*
**Ancestral fixed CF, derived fixed SF**	(2)	207	*4.9*	83	*6.7*	173	*8.1*
**Polymorphism CF, ancestral fixed SF**	(3)	495	*11.8*	154	*12.4*	317	*14.9*
**Polymorphism CF, derived fixed SF**	(4)	123	*2.9*	57	*4.6*	94	*4.4*
**Polymorphism SF, ancestral fixed CF**	(5)	2,105	*50.0*	609	*49.0*	935	*43.8*
**Polymorphism SF, derived fixed CF**	(6)	414	*9.8*	100	*8.0*	177	*8.3*
**Shared polymorphism**	(7)	400	*9.5*	117	*9.4*	187	*8.8*
**Divergent**	(8)	2	*0.0*	1	*0.1*	4	*0.2*
**Total**		5,387	*100.0*	1,244	*100.0*	2,134	*100.0*
**Polymorphism SF**	(5+6+7)	2,919		826		1,299	
**Polymorphism CF**	(3+4+7)	1,018		328		598	
**Ratio SF/CF**		2.87		2.52		2.17	
**Derived and fixed SF**	(2+4)	330		140		267	
**Derived and fixed CF**	(1+6)	876		223		424	
**Ratio CF/SF**		2.65		1.59		1.59	

Thresholds: 100; MAF > 1%; Score Blast < 10^{-5}; interval > 50bp (see materials and methods for threshold definitions).
CF: Cavefish; SF: Surface fish; numbers in brackets are class identifiers described in fig. 4.

Table S3a:

Best score and age of the cavefish population with different sets of demographic parameters.

Ne ancestral = 10,000

Ne SF = 5,000 (% of migrant fish)

Ne CF	Migration rate	1% score	1% age	0.1% score	0.1% age	0.01% score	0.01% age	0% score	0% age
75	0.10								
	0.01								
	0.001								
	0.0001								
	0.00001								
	0.0							15.81	1,200
150	0.10	53.19	73,800						
	0.01	1.63	90,700						
	0.001	9.47	2,300						
	0.0001	12.87	2,200						
	0.00001	13.03	2,000						
	0.0							12.07	2,400
313	0.10	94.76	63,600						
	0.01	4.30	90,400						
	0.001	3.95	13,900						
	0.0001	5.81	4,100						
	0.00001	5.63	5,300						
	0.0							6.92	4,800
625	0.10	118.60	89,900						
	0.01	28.69	49,700						
	0.001	2.01	11,600						
	0.0001	1.03	9,300						
	0.00001	1.00	11,200						
	0.0							2.81	10,800
1,250	0.10	123.32	77,600	63.99	80,000				
	0.01	55.39	94,600	9.24	17,200				
	0.001	11.52	19,700	5.35	17,000				
	0.0001	7.60	16,300	6.78	15,700				
	0.00001	8.14	16,400	7.08	16,100				
	0.0							6.14	13,800
2,500	0.10	128.43	64,900	94.14	91,600				
	0.01	98.60	97,300	41.88	18,600				
	0.001	46.00	17,600	35.05	21,900				
	0.0001	30.64	21,000	30.83	19,700				
	0.00001	30.16	19,300	31.41	25,000				
	0.0							30.10	21,100
5,000	0.10	129.69	97,100	111.16	93,900				
	0.01	117.72	81,800	85.92	17,500				
	0.001	76.51	15,500	67.12	28,700				
	0.0001	69.12	22,900	69.84	33,500				
	0.00001	75.10	12,100	75.07	18,000				
	0.0							63.98	20,300
10,000	0.10	129.75	99,700	123.88	80,800	96.26	12,300		
	0.01	130.87	87,200	109.00	12,600	99.99	13,500		
	0.001	104.11	9,300	98.88	17,600	102.56	13,400		
	0.0001	99.72	14,500	94.96	9,600	92.89	14,000		
	0.00001	99.51	9,300	101.17	9,000	95.71	12,800		
	0.0							96.86	12,700

Ne SF = 10,000 (% of migrant fish)

Ne CF	Migration rate	1% score	1% age	0.1% score	0.1% age	0.01% score	0.01% age	0% score	0% age
75	0.10								
	0.01								
	0.001								
	0.0001								
	0.00001								
	0.0							15.81	1,000
150	0.10	51.19	76,300						
	0.01	1.37	72,100						
	0.001	15.97	1,900						
	0.0001	16.47	2,000						
	0.00001	16.77	2,300						
	0.0							10.80	2,300
313	0.10	102.20	39,300						
	0.01	4.48	50,100						
	0.001	8.10	37,500						
	0.0001	10.64	4,800						
	0.00001	8.93	4,300						
	0.0							12.90	4,700
625	0.10	123.54	68,000						
	0.01	24.18	23,700						
	0.001	1.06	28,600						
	0.0001	5.51	10,300						
	0.00001	5.28	10,200						
	0.0							4.49	8,900
1,250	0.10	133.67	54,400	54.15	72,600				
	0.01	50.80	28,100	1.27	27,100				
	0.001	0.68	21,500	1.13	21,100				
	0.0001	1.71	20,500	1.47	21,100				
	0.00001	1.06	19,100	1.54	19,000				
	0.0							1.45	20,600
2,500	0.10	137.76	8,300	76.99	95,000				
	0.01	83.03	30,400	13.29	36,700				
	0.001	12.06	26,700	8.09	27,900				
	0.0001	6.65	32,300	7.99	29,100				
	0.00001	6.82	32,600	7.22	28,100				
	0.0							8.39	30,800
5,000	0.10	141.32	96,100	107.74	32,600				
	0.01	103.27	40,700	53.69	28,600				
	0.001	41.08	40,400	27.74	49,600				
	0.0001	27.95	42,100	29.32	54,500				
	0.00001	29.37	44,700	25.11	37,300				
	0.0							29.27	44,300
10,000	0.10	142.90	72,900	114.90	8,700	56.75	47,900		
	0.01	115.02	20,500	80.04	20,400	61.27	28,200		
	0.001	79.82	38,000	53.87	39,200	58.48	48,100		
	0.0001	56.67	39,000	59.37	45,700	55.21	29,200		
	0.00001	57.47	41,000	59.44	43,600	56.06	42,100		
	0.0							60.38	35,700

Ne SF = 20,000 (% of migrant fish)

Ne CF	Migration rate	1% score	1% age	0.1% score	0.1% age	0.01% score	0.01% age	0% score	0% age
75	0.10								
	0.01								
	0.001								
	0.0001								
	0.00001								
	0.0							19.06	900
150	0.10	47.53	14,700						
	0.01	5.14	82,500						
	0.001	14.47	54,300						
	0.0001	15.97	1,900						
	0.00001	16.94	2,200						
	0.0							15.34	2,100
313	0.10	93.05	47,300						
	0.01	7.63	48,000						
	0.001	4.95	83,500						
	0.0001	13.76	3,800						
	0.00001	13.20	4,200						
	0.0							11.99	3,800
625	0.10	110.15	27,400						
	0.01	13.41	30,200						
	0.001	3.11	74,800						
	0.0001	10.21	9,800						
	0.00001	8.81	10,100						
	0.0							8.63	8,800
1,250	0.10	120.07	20,500	45.95	19,100				
	0.01	44.52	24,800	2.03	73,200				
	0.001	2.04	51,000	4.80	21,500				
	0.0001	3.56	22,300	3.79	18,100				
	0.00001	4.40	19,700	5.12	16,700				
	0.0							4.19	17,800
2,500	0.10	123.72	25,400	68.34	23,000				
	0.01	72.82	17,200	5.65	76,200				
	0.001	8.72	51,000	5.35	37,100				
	0.0001	5.58	34,600	5.02	35,100				
	0.00001	5.34	34,200	4.11	31,000				
	0.0							4.70	34,300
5,000	0.10	127.73	20,900	94.27	24,800				
	0.01	90.60	30,800	31.37	65,400				
	0.001	23.38	85,100	13.87	65,200				
	0.0001	12.24	66,900	13.01	50,000				
	0.00001	13.13	61,800	13.07	67,900				
	0.0							12.37	56,600
10,000	0.10	124.72	23,600	102.74	29,400	29.37	74,100		
	0.01	97.61	13,000	55.67	51,500	29.65	90,800		
	0.001	53.97	42,700	34.13	97,200	31.40	94,000		
	0.0001	33.37	84,100	31.26	78,100	31.83	96,000		
	0.00001	28.88	96,900	31.94	61,000	31.15	80,800		
	0.0							31.59	93,100

For each simulation, the best score (left value) and the age of the cavefish population (right value) are given.
score < 3, green background; 3 < score < 15, orange background; score > 15, red background.
When the percentage of migrants is less than one fish at each migration, the model is equivalent to the model without migration and the cell was colored in grey.

Table S3b:

Best score and age of the cavefish population with different sets of demographic parameters.

Ne ancestral = 20,000

Ne SF = 5,000 (% of migrant fish)

Ne CF	Migration rate	1% score	1% age	0.1% score	0.1% age	0.01% score	0.01% age	0% score	0% age
75	0.10								
75	0.01								
75	0.001								
75	0.0001								
75	0.00001								
75	0.0							20.39	900
150	0.10	56.72	78,000						
150	0.01	1.59	80,600						
150	0.001	17.61	2,200						
150	0.0001	15.45	1,900						
150	0.00001	15.32	2,100						
150	0.0							14.62	2,200
313	0.10	98.33	66,600						
313	0.01	3.16	94,100						
313	0.001	8.16	5,500						
313	0.0001	7.82	4,800						
313	0.00001	8.98	4,400						
313	0.0							9.92	4,100
625	0.10	116.35	99,300						
625	0.01	29.57	92,000						
625	0.001	3.74	10,100						
625	0.0001	3.23	9,900						
625	0.00001	5.72	10,100						
625	0.0							3.27	8,700
1,250	0.10	130.78	99,300	67.63	98,200				
1,250	0.01	56.11	99,600	16.82	17,900				
1,250	0.001	14.01	13,100	11.95	15,300				
1,250	0.0001	11.11	14,600	11.50	14,200				
1,250	0.00001	10.53	13,400	11.89	14,000				
1,250	0.0							9.70	14,900
2,500	0.10	133.00	94,800	105.77	73,900				
2,500	0.01	101.60	81,800	56.16	16,100				
2,500	0.001	51.63	16,300	38.42	19,300				
2,500	0.0001	46.09	23,700	44.90	18,400				
2,500	0.00001	42.86	21,100	43.48	20,600				
2,500	0.0							39.55	20,600
5,000	0.10	136.44	88,200	117.40	99,600				
5,000	0.01	124.11	83,400	105.31	13,900				
5,000	0.001	101.21	10,600	90.36	22,300				
5,000	0.0001	90.47	15,900	87.95	13,700				
5,000	0.00001	89.50	27,200	92.71	16,300				
5,000	0.0							84.13	14,900
10,000	0.10	136.69	91,500	130.94	92,300	119.40	13,800		
10,000	0.01	131.75	94,000	121.94	7,500	122.98	12,800		
10,000	0.001	126.12	9,800	118.62	12,700	119.23	14,600		
10,000	0.0001	122.95	8,400	118.28	13,300	117.15	7,600		
10,000	0.00001	120.00	11,800	121.37	10,300	118.15	9,600		
10,000	0.0							120.72	12,000

Ne SF = 10,000 (% of migrant fish)

Ne CF	Migration rate	1% score	1% age	0.1% score	0.1% age	0.01% score	0.01% age	0% score	0% age
75	0.10								
75	0.01								
75	0.001								
75	0.0001								
75	0.00001								
75	0.0							20.27	1,000
150	0.10	59.03	94,600						
150	0.01	3.18	90,100						
150	0.001	17.28	1,600						
150	0.0001	19.77	1,900						
150	0.00001	20.55	1,700						
150	0.0							19.88	1,800
313	0.10	110.68	86,300						
313	0.01	6.74	96,400						
313	0.001	10.89	34,600						
313	0.0001	16.63	4,600						
313	0.00001	12.76	5,000						
313	0.0							14.01	4,900
625	0.10	128.52	93,000						
625	0.01	24.23	70,800						
625	0.001	3.14	27,000						
625	0.0001	6.18	9,000						
625	0.00001	7.56	9,500						
625	0.0							7.75	8,800
1,250	0.10	144.17	98,600	60.04	58,000				
1,250	0.01	63.25	88,500	2.54	24,600				
1,250	0.001	2.90	25,400	2.11	19,400				
1,250	0.0001	1.35	18,900	3.24	20,300				
1,250	0.00001	1.63	18,900	1.74	17,900				
1,250	0.0							2.51	16,400
2,500	0.10	157.76	94,700	88.14	80,700				
2,500	0.01	95.49	97,900	18.84	37,800				
2,500	0.001	24.34	28,600	9.30	29,000				
2,500	0.0001	8.29	29,000	7.97	29,900				
2,500	0.00001	7.23	29,700	6.69	29,900				
2,500	0.0							8.13	29,200
5,000	0.10	150.63	76,500	119.71	99,700				
5,000	0.01	117.80	74,100	57.54	32,500				
5,000	0.001	57.12	43,100	31.49	45,100				
5,000	0.0001	37.59	34,500	32.99	46,200				
5,000	0.00001	32.21	45,200	31.99	44,300				
5,000	0.0							33.02	49,000
10,000	0.10	148.02	80,200	124.50	88,100	68.76	35,100		
10,000	0.01	124.62	96,000	97.47	28,900	73.38	66,000		
10,000	0.001	93.29	25,900	78.34	59,900	74.66	44,300		
10,000	0.0001	73.06	45,400	74.58	81,100	68.74	42,100		
10,000	0.00001	74.31	46,300	72.89	58,400	68.20	61,000		
10,000	0.0							74.07	41,700

Ne SF = 20,000 (% of migrant fish)

Ne CF	Migration rate	1% score	1% age	0.1% score	0.1% age	0.01% score	0.01% age	0% score	0% age
75	0.10								
75	0.01								
75	0.001								
75	0.0001								
75	0.00001								
75	0.0							22.47	1,000
150	0.10	61.23	52,200						
150	0.01	6.47	99,500						
150	0.001	14.85	42,700						
150	0.0001	21.53	1,800						
150	0.00001	19.52	2,300						
150	0.0							18.64	2,200
313	0.10	110.36	28,300						
313	0.01	9.80	51,900						
313	0.001	4.87	97,100						
313	0.0001	17.96	3,700						
313	0.00001	17.49	4,300						
313	0.0							15.87	3,900
625	0.10	128.01	15,600						
625	0.01	26.98	91,400						
625	0.001	1.50	90,900						
625	0.0001	9.93	8,900						
625	0.00001	11.73	8,700						
625	0.0							11.72	8,700
1,250	0.10	140.36	87,700	57.64	70,200				
1,250	0.01	53.67	26,600	0.29	54,200				
1,250	0.001	0.81	38,600	5.45	19,100				
1,250	0.0001	4.39	23,000	5.02	17,200				
1,250	0.00001	5.31	20,500	4.62	20,000				
1,250	0.0							4.50	20,100
2,500	0.10	147.32	90,900	86.35	76,500				
2,500	0.01	79.54	61,600	4.30	51,200				
2,500	0.001	4.99	60,800	1.48	37,600				
2,500	0.0001	1.31	51,300	0.90	37,100				
2,500	0.00001	1.49	38,400	1.61	38,300				
2,500	0.0							1.73	33,500
5,000	0.10	146.97	93,900	114.89	22,200				
5,000	0.01	113.56	66,700	34.72	89,500				
5,000	0.001	31.00	59,600	7.98	67,000				
5,000	0.0001	9.23	69,200	8.41	56,200				
5,000	0.00001	7.69	66,800	7.49	62,100				
5,000	0.0							6.78	57,000
10,000	0.10	152.48	20,300	127.31	95,600	26.73	83,600		
10,000	0.01	124.49	62,300	65.85	57,200	29.74	83,200		
10,000	0.001	69.36	57,900	32.53	76,400	29.89	82,100		
10,000	0.0001	33.29	95,100	32.94	96,700	29.91	92,300		
10,000	0.00001	31.85	92,700	27.34	83,200	29.43	99,000		
10,000	0.0							29.49	86,200

For each simulation, the best score (left value) and the age of the cavefish population (right value) are given.
score < 3, green background; 3 < score < 15, orange background; score > 15, red background.
When the percentage of migrants is less than one fish at each migration, the model is equivalent to the model without migration and the cell was colored in grey.

Table S3c:

Best score and age of the cavefish population with different sets of demographic parameters.

Ne ancestral = 50,000

Ne SF = 5,000 (% of migrant fish)

Ne CF	Migration rate	1%		0.1%		0.01%		0%	
75	0.10								
	0.01								
	0.001								
	0.0001								
	0.00001								
	0.0							20.21	900
150	0.10	54.47	95,300						
	0.01	2.33	92,100						
	0.001	15.24	1,900						
	0.0001	16.63	2,100						
	0.00001	16.59	2,100						
	0.0							18.17	2,100
313	0.10	103.91	81,400						
	0.01	5.58	97,100						
	0.001	11.70	5,600						
	0.0001	11.93	5,000						
	0.00001	11.58	4,100						
	0.0							11.31	4,600
625	0.10	126.61	99,500						
	0.01	29.82	86,600						
	0.001	8.66	11,500						
	0.0001	6.88	9,000						
	0.00001	6.39	9,200						
	0.0							8.21	9,100
1,250	0.10	128.07	95,600	78.10	98,300				
	0.01	75.38	97,200	20.85	16,700				
	0.001	26.42	18,300	16.19	14,000				
	0.0001	18.46	15,800	16.02	14,100				
	0.00001	15.81	15,600	15.94	15,000				
	0.0							16.28	14,700
2,500	0.10	137.62	88,900	108.57	97,800				
	0.01	111.66	96,900	74.49	16,700				
	0.001	69.78	14,500	55.17	20,500				
	0.0001	52.72	19,900	52.24	19,800				
	0.00001	54.76	21,200	51.55	18,600				
	0.0							52.71	20,100
5,000	0.10	141.77	85,600	131.81	94,500				
	0.01	128.05	97,500	123.23	9,800				
	0.001	124.25	9,000	108.76	15,000				
	0.0001	107.41	11,700	104.53	16,700				
	0.00001	109.24	10,500	108.51	11,600				
	0.0							105.16	15,100
10,000	0.10	138.94	99,000	139.14	98,100	135.90	9,900		
	0.01	143.98	98,500	139.04	9,200	131.51	8,700		
	0.001	140.97	7,000	136.75	6,400	133.83	10,200		
	0.0001	132.54	8,300	132.65	7,300	137.58	9,000		
	0.00001	133.45	8,900	133.46	7,600	137.86	9,600		
	0.0							133.05	8,300

Ne SF = 10,000 (% of migrant fish)

Ne CF	Migration rate	1%		0.1%		0.01%		0%	
75	0.10								
	0.01								
	0.001								
	0.0001								
	0.00001								
	0.0							23.38	900
150	0.10	73.29	95,000						
	0.01	8.47	98,400						
	0.001	19.39	1,800						
	0.0001	19.06	1,800						
	0.00001	19.71	1,900						
	0.0							20.80	2,100
313	0.10	128.87	96,400						
	0.01	11.80	82,100						
	0.001	14.58	29,800						
	0.0001	17.34	4,100						
	0.00001	15.56	4,000						
	0.0							15.55	4,100
625	0.10	145.84	900						
	0.01	34.06	80,900						
	0.001	7.09	17,300						
	0.0001	10.46	8,900						
	0.00001	9.87	9,700						
	0.0							9.65	8,900
1,250	0.10	154.06	98,700	71.01	86,700				
	0.01	67.07	90,800	6.71	27,800				
	0.001	9.25	28,600	4.46	17,800				
	0.0001	4.94	19,800	5.54	17,300				
	0.00001	4.88	17,600	5.61	19,100				
	0.0							4.26	17,800
2,500	0.10	161.09	300	110.16	95,300				
	0.01	116.81	99,200	26.03	26,800				
	0.001	26.29	36,500	13.03	28,300				
	0.0001	13.44	30,400	14.29	28,200				
	0.00001	13.41	31,000	12.52	29,700				
	0.0							12.30	27,700
5,000	0.10	160.27	92,000	135.08	87,300				
	0.01	138.39	99,600	83.43	25,400				
	0.001	74.78	23,800	48.73	40,600				
	0.0001	47.84	38,900	42.85	42,000				
	0.00001	46.44	34,400	46.17	46,200				
	0.0							43.42	37,500
10,000	0.10	165.52	100	149.29	98,000	93.94	27,800		
	0.01	146.97	97,400	117.59	16,600	97.00	51,800		
	0.001	121.34	14,800	97.46	28,400	93.32	30,700		
	0.0001	99.95	31,800	97.85	36,500	95.02	42,600		
	0.00001	94.06	32,300	93.53	33,800	94.41	39,700		
	0.0							97.90	38,400

Ne SF = 20,000 (% of migrant fish)

Ne CF	Migration rate	1%		0.1%		0.01%		0%	
75	0.10								
	0.01								
	0.001								
	0.0001								
	0.00001								
	0.0							21.20	900
150	0.10	75.05	2,500						
	0.01	17.83	76,400						
	0.001	20.21	2,200						
	0.0001	21.12	2,200						
	0.00001	21.63	1,900						
	0.0							20.49	2,200
313	0.10	124.06	3,900						
	0.01	18.27	30,000						
	0.001	11.74	64,800						
	0.0001	18.39	4,400						
	0.00001	18.24	4,200						
	0.0							18.63	4,200
625	0.10	145.80	2,400						
	0.01	33.71	86,700						
	0.001	5.96	49,300						
	0.0001	14.70	9,500						
	0.00001	13.64	8,300						
	0.0							14.56	8,600
1,250	0.10	155.98	1,900	75.47	82,900				
	0.01	70.85	92,600	3.85	49,500				
	0.001	3.98	42,400	8.41	21,300				
	0.0001	8.12	25,800	7.38	20,600				
	0.00001	7.24	18,700	8.42	17,600				
	0.0							7.93	17,000
2,500	0.10	161.18	1,100	107.18	12,000				
	0.01	112.16	30,400	8.14	60,700				
	0.001	4.52	52,000	2.46	38,100				
	0.0001	2.82	40,600	2.45	37,400				
	0.00001	2.46	38,500	3.04	36,000				
	0.0							2.91	37,500
5,000	0.10	164.31	300	140.52	7,100				
	0.01	137.42	15,800	47.83	61,400				
	0.001	42.52	66,400	12.25	69,000				
	0.0001	11.20	62,500	8.92	65,200				
	0.00001	9.62	65,400	9.83	56,400				
	0.0							8.05	63,400
10,000	0.10	169.33	1,600	152.84	8,400	37.32	80,600		
	0.01	151.68	6,500	90.36	45,200	36.62	93,900		
	0.001	85.17	49,500	42.27	74,400	36.78	86,700		
	0.0001	44.75	82,300	39.48	84,200	36.29	81,200		
	0.00001	36.57	89,800	36.80	80,500	35.23	84,800		
	0.0							39.34	91,700

For each simulation, the best score (left value) and the age of the cavefish population (right value) are given.
score < 3, green background; 3 < score < 15, orange background; score > 15, red background.
When the percentage of migrants is less than one fish at each migration, the model is equivalent to the model without migration and the cell was colored in grey.

Table S3d:

Best score and age of the cavefish population with different sets of demographic parameters.

Ne ancestral = 100,000

Ne SF 5,000 (% of migrant fish)

Ne CF	Migration rate	1% score	1% age	0.1% score	0.1% age	0.01% score	0.01% age	0% score	0% age
75	0.10								
	0.01								
	0.001								
	0.0001								
	0.00001								
	0.0							20.76	900
150	0.10	59.65	99,700						
	0.01	6.43	97,100						
	0.010	18.35	1,900						
	0.0010	16.58	2,100						
	0.00010	17.87	2,100						
	0.0							17.76	1,900
313	0.10	102.85	98,400						
	0.01	7.89	96,900						
	0.010	12.32	5,500						
	0.0010	12.28	4,600						
	0.00010	12.64	4,500						
	0.0							12.11	4,600
625	0.10	125.84	97,300						
	0.01	29.81	95,300						
	0.010	8.62	9,500						
	0.0010	7.66	8,700						
	0.00010	8.79	9,200						
	0.0							8.64	8,700
1,250	0.10	134.35	95,900	85.30	99,900				
	0.01	86.15	91,500	25.25	15,700				
	0.010	23.26	13,100	19.29	13,200				
	0.0010	18.42	14,100	18.49	13,700				
	0.00010	18.98	13,700	17.89	14,100				
	0.0							18.41	13,600
2,500	0.10	140.12	96,600	126.78	99,600				
	0.01	117.38	94,800	75.30	13,600				
	0.010	79.15	17,400	61.05	19,500				
	0.0010	58.31	20,700	57.72	18,700				
	0.00010	57.90	17,300	58.02	21,300				
	0.0							59.20	19,200
5,000	0.10	146.85	98,100	135.37	95,500				
	0.01	149.59	96,200	127.95	7,100				
	0.010	132.81	7,500	115.37	12,500				
	0.0010	119.10	9,600	111.55	14,400				
	0.00010	116.89	12,900	116.57	15,700				
	0.0							114.74	10,600
10,000	0.10	149.54	96,200	152.75	93,500	139.52	9,400		
	0.01	150.84	98,700	148.10	7,800	140.45	6,600		
	0.010	143.42	5,500	142.87	6,700	139.63	8,600		
	0.0010	143.04	6,600	142.16	8,700	141.18	7,800		
	0.00010	143.02	7,300	140.98	7,800	141.52	7,900		
	0.0							143.19	7,800

Ne SF 10,000 (% of migrant fish)

Ne CF	Migration rate	1% score	1% age	0.1% score	0.1% age	0.01% score	0.01% age	0% score	0% age
75	0.10								
	0.01								
	0.001								
	0.0001								
	0.00001								
	0.0							22.42	900
150	0.10	79.90	1,400						
	0.01	17.22	95,800						
	0.010	19.83	2,000						
	0.0010	19.83	1,800						
	0.00010	19.95	1,800						
	0.0							19.76	2,100
313	0.10	131.45	2,200						
	0.01	18.76	95,700						
	0.010	15.65	5,600						
	0.0010	16.88	4,700						
	0.00010	16.96	4,200						
	0.0							15.30	4,600
625	0.10	152.49	1,100						
	0.01	42.27	82,300						
	0.010	9.62	12,600						
	0.0010	11.74	10,400						
	0.00010	10.66	9,600						
	0.0							11.42	9,600
1,250	0.10	161.50	400	79.23	98,600				
	0.01	86.53	10,200	8.57	20,300				
	0.010	11.10	23,400	7.17	17,300				
	0.0010	7.08	18,300	7.05	17,000				
	0.00010	6.97	17,400	6.94	17,100				
	0.0							7.21	18,600
2,500	0.10	168.91	400	126.30	7,100				
	0.01	132.62	8,800	33.71	30,200				
	0.010	33.03	31,500	17.41	29,300				
	0.0010	18.81	31,300	15.24	28,600				
	0.00010	16.23	29,500	15.17	29,000				
	0.0							16.37	27,700
5,000	0.10	170.44	200	152.61	4,800				
	0.01	154.77	8,400	94.58	20,100				
	0.010	96.46	32,500	56.52	44,700				
	0.0010	53.32	40,000	54.84	40,800				
	0.00010	51.84	42,900	51.58	38,600				
	0.0							54.03	42,200
10,000	0.10	171.25	400	161.04	3,400	109.11	30,400		
	0.01	160.53	2,700	128.97	15,300	107.31	29,500		
	0.010	129.57	21,000	113.11	23,200	105.76	33,300		
	0.0010	108.58	32,100	109.46	26,300	109.94	31,600		
	0.00010	107.77	34,100	109.31	31,100	106.74	28,900		
	0.0							107.89	39,300

Ne SF 20,000 (% of migrant fish)

Ne CF	Migration rate	1% score	1% age	0.1% score	0.1% age	0.01% score	0.01% age	0% score	0% age
75	0.10								
	0.01								
	0.001								
	0.0001								
	0.00001								
	0.0							23.50	900
150	0.10	75.36	2,200						
	0.01	20.00	23,400						
	0.010	22.00	2,000						
	0.0010	20.83	2,100						
	0.00010	21.34	1,800						
	0.0							21.46	1,800
313	0.10	129.33	2,700						
	0.01	22.03	8,200						
	0.010	15.96	38,300						
	0.0010	19.83	4,200						
	0.00010	19.99	4,100						
	0.0							20.00	3,900
625	0.10	147.10	1,800						
	0.01	36.50	10,300						
	0.010	12.46	70,300						
	0.0010	15.64	8,100						
	0.00010	15.80	7,600						
	0.0							14.97	8,900
1,250	0.10	161.63	700	84.25	11,600				
	0.01	86.22	26,300	7.94	32,500				
	0.010	6.91	43,100	9.43	19,500				
	0.0010	11.42	18,100	10.16	16,300				
	0.00010	9.38	17,500	10.30	16,700				
	0.0							10.32	18,600
2,500	0.10	163.56	1,100	122.14	7,800				
	0.01	120.50	10,400	11.60	47,400				
	0.010	9.24	41,700	5.14	38,000				
	0.0010	5.17	39,000	5.39	36,900				
	0.00010	4.61	38,700	5.19	34,600				
	0.0							5.02	36,100
5,000	0.10	167.40	1,600	148.32	6,000				
	0.01	148.23	9,200	57.65	46,900				
	0.010	45.58	73,400	15.39	63,500				
	0.0010	16.23	61,800	13.23	60,800				
	0.00010	13.49	57,600	13.16	58,700				
	0.0							13.21	60,300
10,000	0.10	170.03	0	157.85	5,800	46.53	80,400		
	0.01	157.54	5,900	101.47	40,300	45.33	88,300		
	0.010	100.48	40,600	53.09	90,900	45.54	93,000		
	0.0010	50.80	71,400	47.70	92,000	43.87	83,100		
	0.00010	47.97	88,100	46.00	79,600	47.42	81,600		
	0.0							47.26	89,600

For each simulation, the best score (left value) and the age of the cavefish population (right value) are given.
score < 3, green background; 3 < score < 15, orange background; score > 15, red background.
When the percentage of migrants is less than one fish at each migration, the model is equivalent to the model without migration and the cell was colored in grey.
