# Supplementary material for: Evidence for late Pleistocene origin of Astyanax mexicanus cavefish
Source: BMC Evol Biol. 2018 Apr 18;18:43. doi: 10.1186/s12862-018-1156-7 (PMC5905186; doi:10.1186/s12862-018-1156-7)
Supplement: Supplementary file 2 — Four taxon unrooted nuclear gene phylogenies. (PDF 101 kb) [file 12862_2018_1156_MOESM2_ESM.pdf]

Mutation mapping in *A. mexicanus* nuclear gene most parsimonious unrooted trees

Mc1r (complete coding sequence, 972 bp)

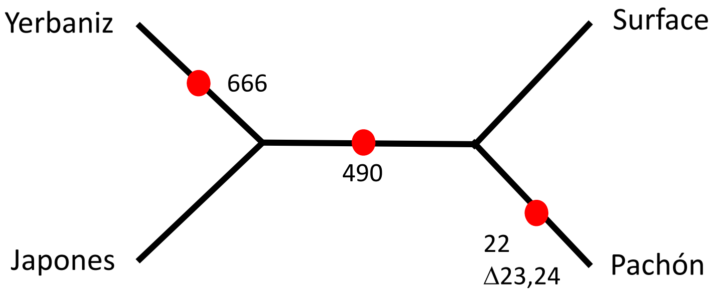

Positions of the mutations

22: C/T non synonymous
Δ23,24: 2 bp deletion
490: C/T non synonymous
666: G/A synonymous

Note: 'Pachon' haplotype is found also in Yerbaniz and Japones

Accession numbers: FJ665983, FJ665984, FJ665985, FJ665986

Oca2 (complete coding sequence, 2499 bp)

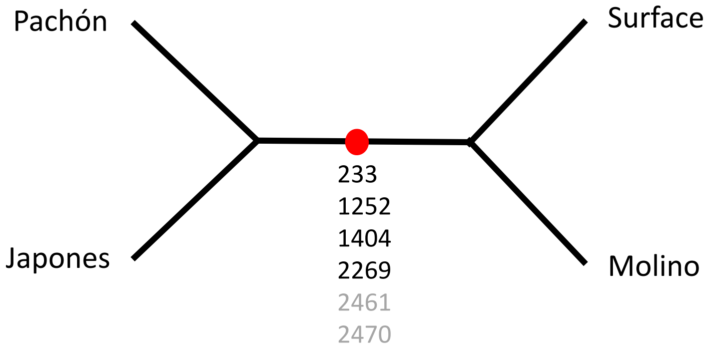

Positions of the mutations

233: G/A non synonymous
1252: G/A non synonymous
1404: C/T synonymous
2269: C/T non synonymous
2461: C/G non synonymous
2470: G/A non synonymous

Note: exon 24 containing position 2461 and 2470 is deleted in Pachon sequence.

Accession numbers: no accession numbers, based on Supplementary Table 2 in Protas ME *et al.*: Genetic analysis of cavefish reveals molecular convergence in the evolution of albinism. Nat Genet 2006, 38(1):107-111.

Mc4r (complete coding sequence, 1008 bp)

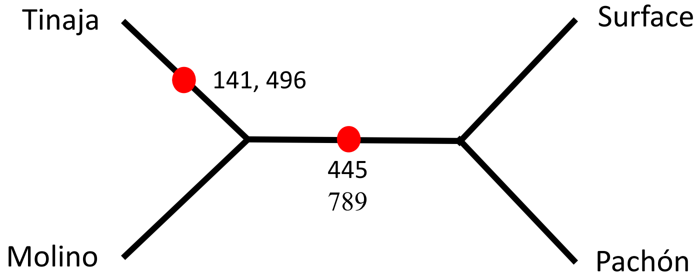

Positions of the mutations

141: C/T synonymous
445: G/A non synonymous
496: G/A non synonymous
789: G/T non synonymous

Accession numbers: no accession numbers, sequenes found a Supporting Information File of Aspiras AC *et al.*: Melanocortin 4 receptor mutations contribute to the adaptation of cavefish to nutrient-poor conditions. Proceedings of the National Academy of Sciences 2015, 112(31):9668-9673.

Mc3r (complete coding sequence, 1158 bp)

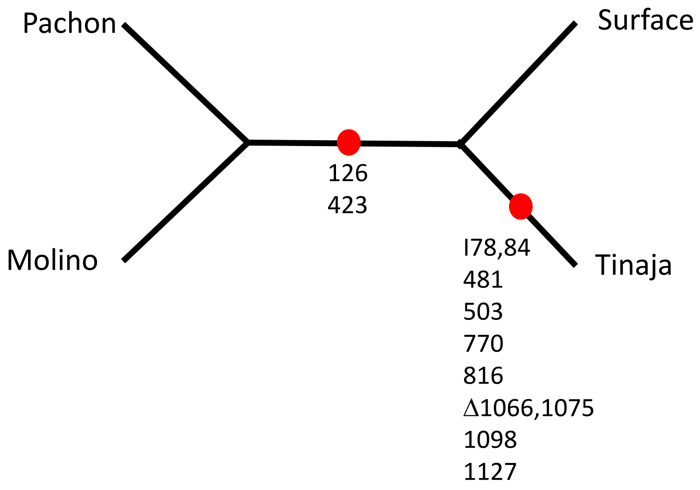

Positions of the mutations

I78,84: 6 bp insertion
126: G/A synonymous
423: T/C synonymous
481: G/A non synonymous
503: T/C non synonymous
770: G/C non synonymous
816: T/C synonymous
∆1066,1075: 12 pb deletion
1098: G/A synonymous
1127: G/A non synonymous

Accession numbers: no accession numbers, sequenes found a Supporting Information File of Aspiras AC *et al.*: Melanocortin 4 receptor mutations contribute to the adaptation of cavefish to nutrient-poor conditions. Proceedings of the National Academy of Sciences 2015, 112(31):9668-9673.

Leptin b (Lepb) (complete coding sequence, 483 bp)

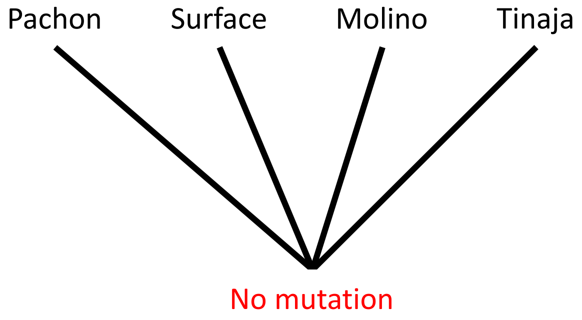

Accession numbers: no accession numbers, sequenes found a Supporting Information File of Aspiras AC *et al.*: Melanocortin 4 receptor mutations contribute to the adaptation of cavefish to nutrient-poor conditions. Proceedings of the National Academy of Sciences 2015, 112(31):9668-9673.

Pomcb (complete coding sequence, 522 bp)

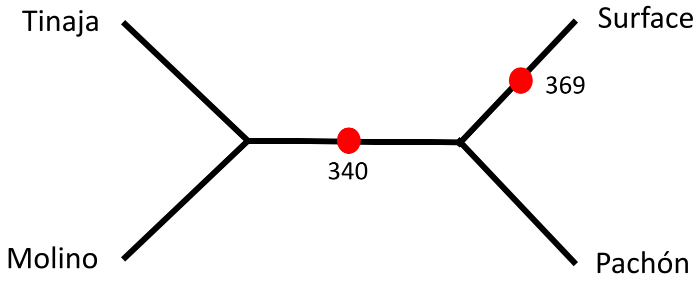

Positions of the mutations

340: G/A non synonymous
369: C/A synonymous

Accession numbers: no accession numbers, sequenes found a Supporting Information File of Aspiras AC *et al.*: Melanocortin 4 receptor mutations contribute to the adaptation of cavefish to nutrient-poor conditions. Proceedings of the National Academy of Sciences 2015, 112(31):9668-9673.

Leptin receptor (Lepr) (complete coding sequence, 3354 bp)

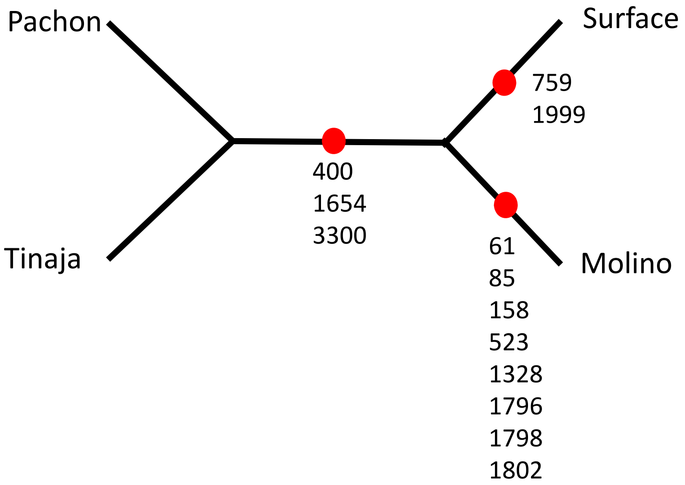

Positions of the mutations

61: G/T non synonymous
85: C/T non synonymous
158: G/C non synonymous
400: G/A non synonymous
523: G/A non synonymous
759: G/T synonymous
1328: C/T non synonymous
1654: G/A non synonymous
1796: G/C non synonymous
1798: G/A non synonymous
1802: C/T non synonymous
1999: G/T non synonymous
3300: G/T synonymous

Accession numbers: no accession numbers, sequenes found a Supporting Information File of Aspiras AC *et al.*: Melanocortin 4 receptor mutations contribute to the adaptation of cavefish to nutrient-poor conditions. Proceedings of the National Academy of Sciences 2015, 112(31):9668-9673.

Informative sites in Mc4r, Mc3r, Lepr and Pomcb

	Genes							
	Mc4r		Mc3r		Lepr			Pomcb
	1	2	3	4	5	6	7	8
Surface	G	G	G	C	G	A	G	G
Pachón	G	G	A	T	A	G	T	G
Tinaja	A	T	G	C	A	G	T	A
Molino	A	T	A	T	G	A	G	A

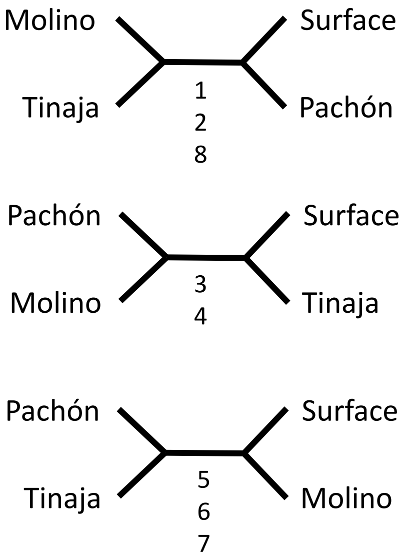

Per1 (complete coding sequence, 4296 bp)

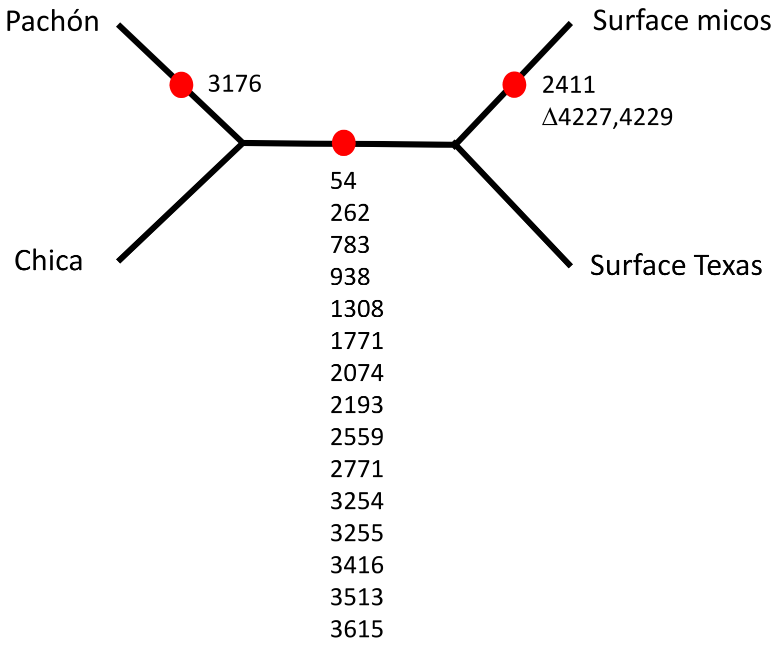

Accession numbers: KF737843, KF737844, KF737845

Positions of the mutations

54: C/T synonymous
262: T/A non synonymous
783: G/A synonymous
938: T/A non synonymous
947: G/A non synonymous
1308: G/C non synonymous
1771: G/A non synonymous
2074: G/C non synonymous
2193: G/C synonymous
2411: T/C non synonymous
2559: G/A synonymous
2771 G/A non synonymous
3176: C/T non synonymous
3254: C/A non synonymous
3255: C/T synonymous
3416: G/A non synonymous
3513: G/T non synonymous
3254: C/A non synonymous
3615: G/A synonymous
Δ4227,4229 : 3 bp deletion

Per2 (coding sequence, 481 bp)

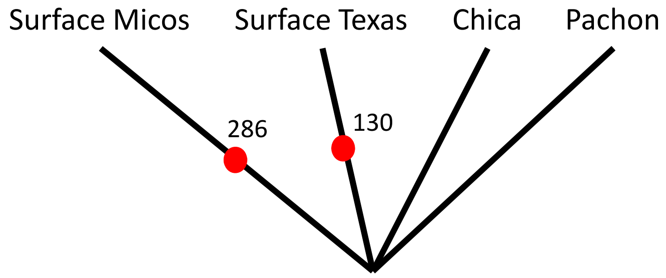

Accession numbers: KF737849, KF737850, KF737851

Positions of the mutations

130: C/T synonymous
286: G/A synonymous

Tef1 (coding sequence, 396 bp)

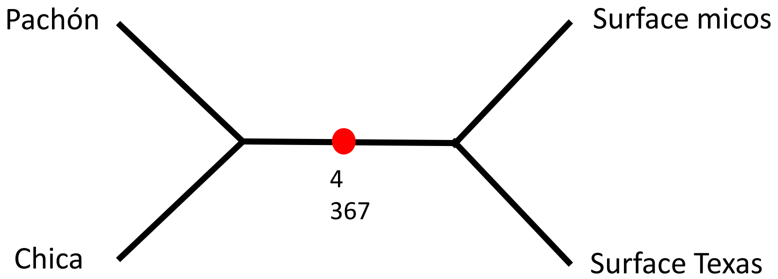

Accession numbers: KF737852, KF737853, KF737854

Positions of the mutations

4: G/A synonymous
367: G/T non synonymous

Cry1a (complete coding sequence, 1884 bp)

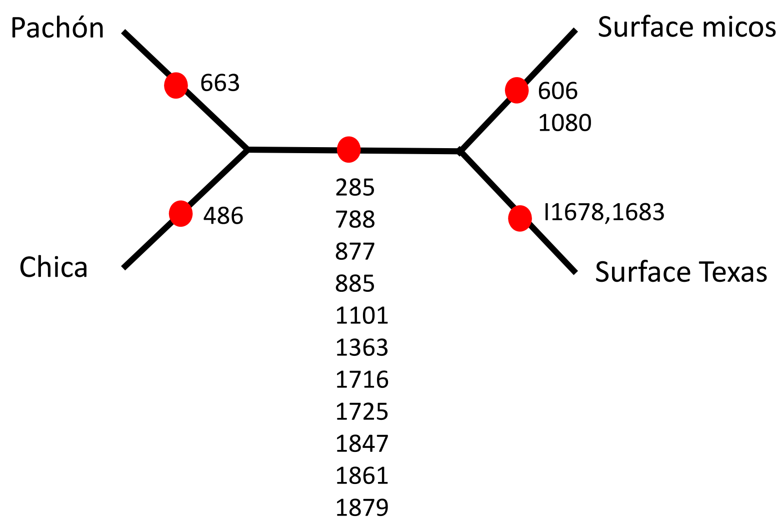

Positions of the mutations

285: G/T synonymous
486: G/A synonymous
606: C/A synonymous
663: C/T synonymous
788: G/A non synonymous
877: C/A non synonymous
885: C/T synonymous
1080: C/T synonymous
1101: G/C synonymous
1363: C/A non synonymous
I1678,1683: 6 pb insertion
1716: C/A synonymous
1725: C/T synonymous
1847: C/T non synonymous
1861: G/C non synonymous
1879: C/A non synonymous

Accession numbers: KF737846, KF737847, KF737848

Cpd photolyase (complete coding sequence, 890 bp)

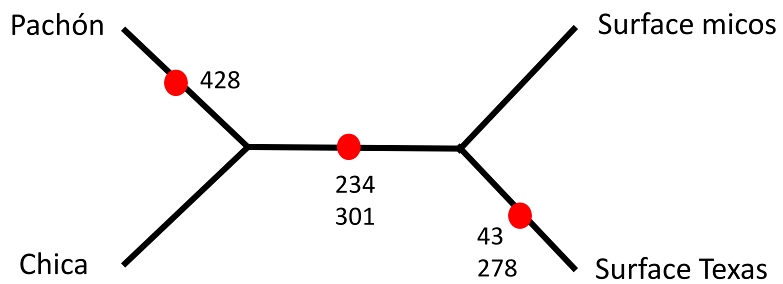

Positions of the mutations

43: C/Y non synonymous
234: T/C/Y synonymous
278: C/Y non synonymous
301: G/A non synonymous
428: C/T non synonymous

Note: Surface Texas population is polymorphic (T,C) at position 43, 234, 278.

Accession numbers: KF737857, KF737858, KF737859
