# Supplementary material for: Evidence for late Pleistocene origin of Astyanax mexicanus cavefish
Source: BMC Evol Biol. 2018 Apr 18;18:43. doi: 10.1186/s12862-018-1156-7 (PMC5905186; doi:10.1186/s12862-018-1156-7)
Supplement: Supplementary file 3 — Allele frequency distributions at 25 microsatellite loci. (PDF 2321 kb) [file 12862_2018_1156_MOESM3_ESM.pdf]

Additional Information for

Evidence of late Pleistocene origin of *Astyanax mexicanus* cavefish

Julien Fumey[1,2], Hélène Hinaux[3], Céline Noirot[4], Claude Thermes[2], Sylvie Rétaux[3] and Didier Casane[1,5,*]

[1] Évolution, Génomes, Comportement, Écologie. CNRS, IRD, Univ Paris-Sud. Université Paris-Saclay. F-91198 Gif-sur-Yvette, France.

[2] Institute for Integrative Biology of the Cell (I2BC), CEA, CNRS, Université Paris-Sud, UMR 9198, FRC 3115, Avenue de la Terrasse, Bâtiment 24, Gif-sur-Yvette, Paris F-91198, France.

[3] DECA group, Paris-Saclay Institute of Neuroscience, UMR 9197, CNRS, Gif sur Yvette, France.

[4] Plateforme Bioinformatique Toulouse, Midi-Pyrénées, UBIA, INRA, Auzeville Castanet-Tolosan, France

[5] Université Paris Diderot, Sorbonne Paris Cité, France.

* Corresponding author:

Didier Casane

Laboratoire Évolution, Génomes, Comportement, Écologie, UMR 9191 CNRS, 1 avenue de la Terrasse, 91198 Gif sur Yvette, France.

Tel: +33169823759

Email: Didier.Casane@egce.cnrs-gif.fr

Figure S1 to S26

Distribution of allele frequencies at 26 locus in 15 populations studied by Bradic at al. (2012)

See Figure 1 for the geographic distribution of the populations.

Four groups of surface populations (S1, S2, S3, S4)

Three “new” cave populations (N1: Molino, N2: Caballo Moro, N3: Subterráneo)

Eight “old” cave populations (O1: Pachón, O2: Yerbaniz, O3: Japonés, O4: Arroyo, O5: Tinaja, O6: Curva, O7: Toro, O8: Chica

For each population, the first, second third and fourth most frequent allele are respectively colored in red, orange, yellow and green. If the frequency is below 10%, the allele is colored in black.

For each population it is also indicated the number of allele scored, the number of different alleles (N) and the effective number of allele (Ne).

Figure S1

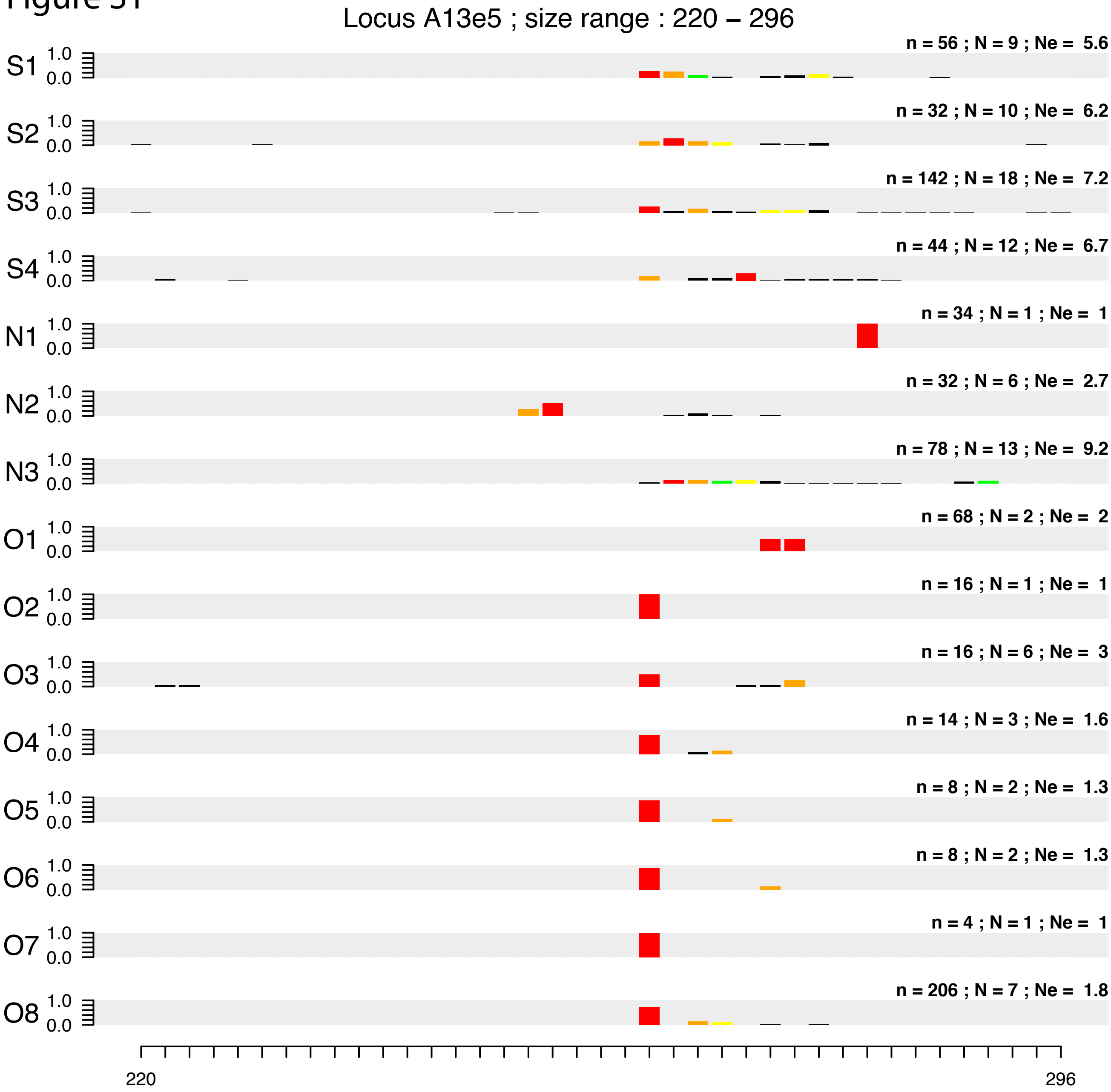
Locus A13e5 ; size range : 220 – 296
S1
n = 56 ; N = 9 ; Ne = 5.6
S2
n = 32 ; N = 10 ; Ne = 6.2
S3
n = 142 ; N = 18 ; Ne = 7.2
S4
n = 44 ; N = 12 ; Ne = 6.7
N1
n = 34 ; N = 1 ; Ne = 1
N2
n = 32 ; N = 6 ; Ne = 2.7
N3
n = 78 ; N = 13 ; Ne = 9.2
O1
n = 68 ; N = 2 ; Ne = 2
O2
n = 16 ; N = 1 ; Ne = 1
O3
n = 16 ; N = 6 ; Ne = 3
O4
n = 14 ; N = 3 ; Ne = 1.6
O5
n = 8 ; N = 2 ; Ne = 1.3
O6
n = 8 ; N = 2 ; Ne = 1.3
O7
n = 4 ; N = 1 ; Ne = 1
O8
n = 206 ; N = 7 ; Ne = 1.8
1.0
0.0
220
296

Figure S2

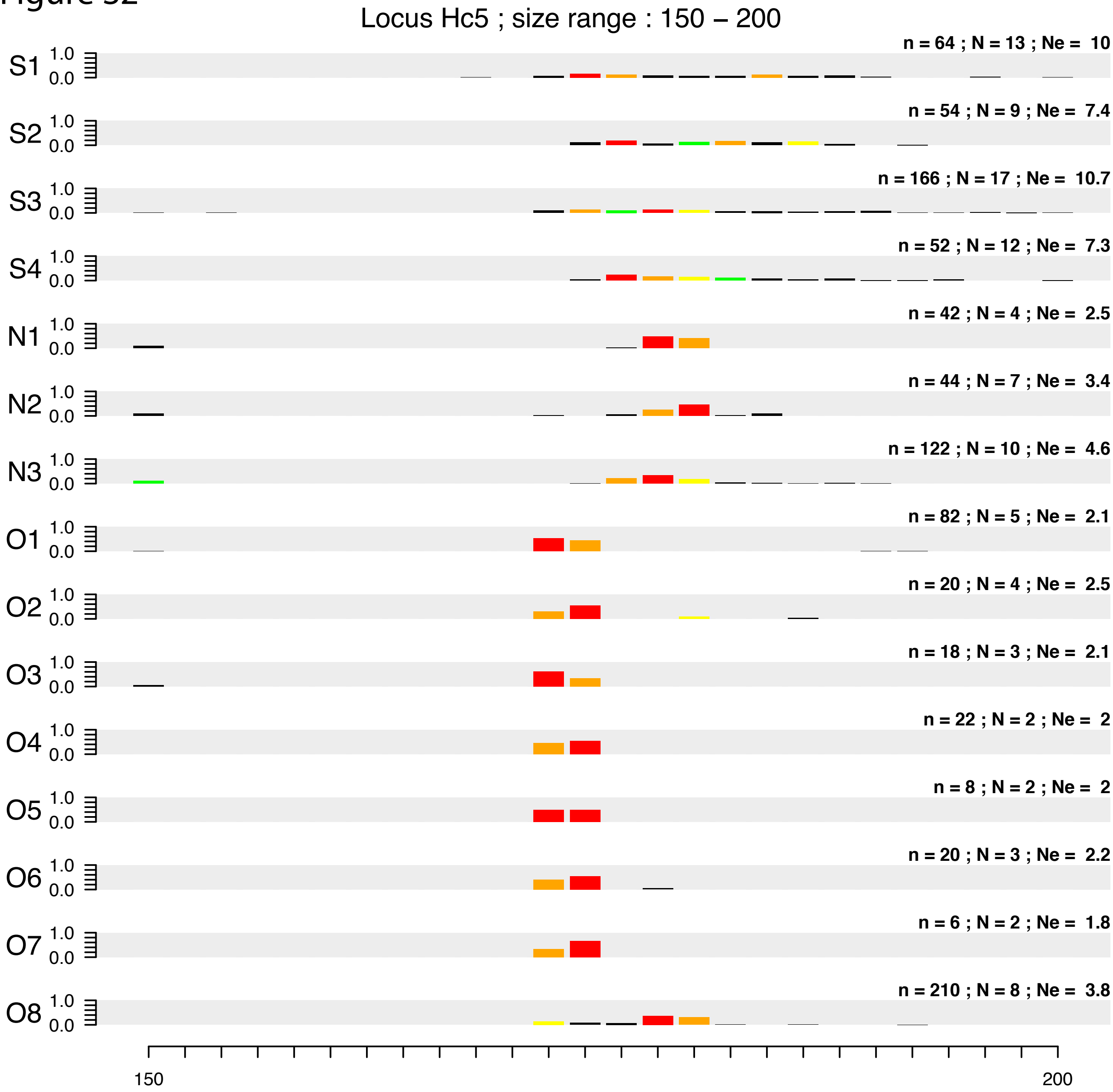
Locus Hc5 ; size range : 150 – 200
S1
n = 64 ; N = 13 ; Ne = 10
S2
n = 54 ; N = 9 ; Ne = 7.4
S3
n = 166 ; N = 17 ; Ne = 10.7
S4
n = 52 ; N = 12 ; Ne = 7.3
N1
n = 42 ; N = 4 ; Ne = 2.5
N2
n = 44 ; N = 7 ; Ne = 3.4
N3
n = 122 ; N = 10 ; Ne = 4.6
O1
n = 82 ; N = 5 ; Ne = 2.1
O2
n = 20 ; N = 4 ; Ne = 2.5
O3
n = 18 ; N = 3 ; Ne = 2.1
O4
n = 22 ; N = 2 ; Ne = 2
O5
n = 8 ; N = 2 ; Ne = 2
O6
n = 20 ; N = 3 ; Ne = 2.2
O7
n = 6 ; N = 2 ; Ne = 1.8
O8
n = 210 ; N = 8 ; Ne = 3.8
1.0
0.0
150
200

Figure S3

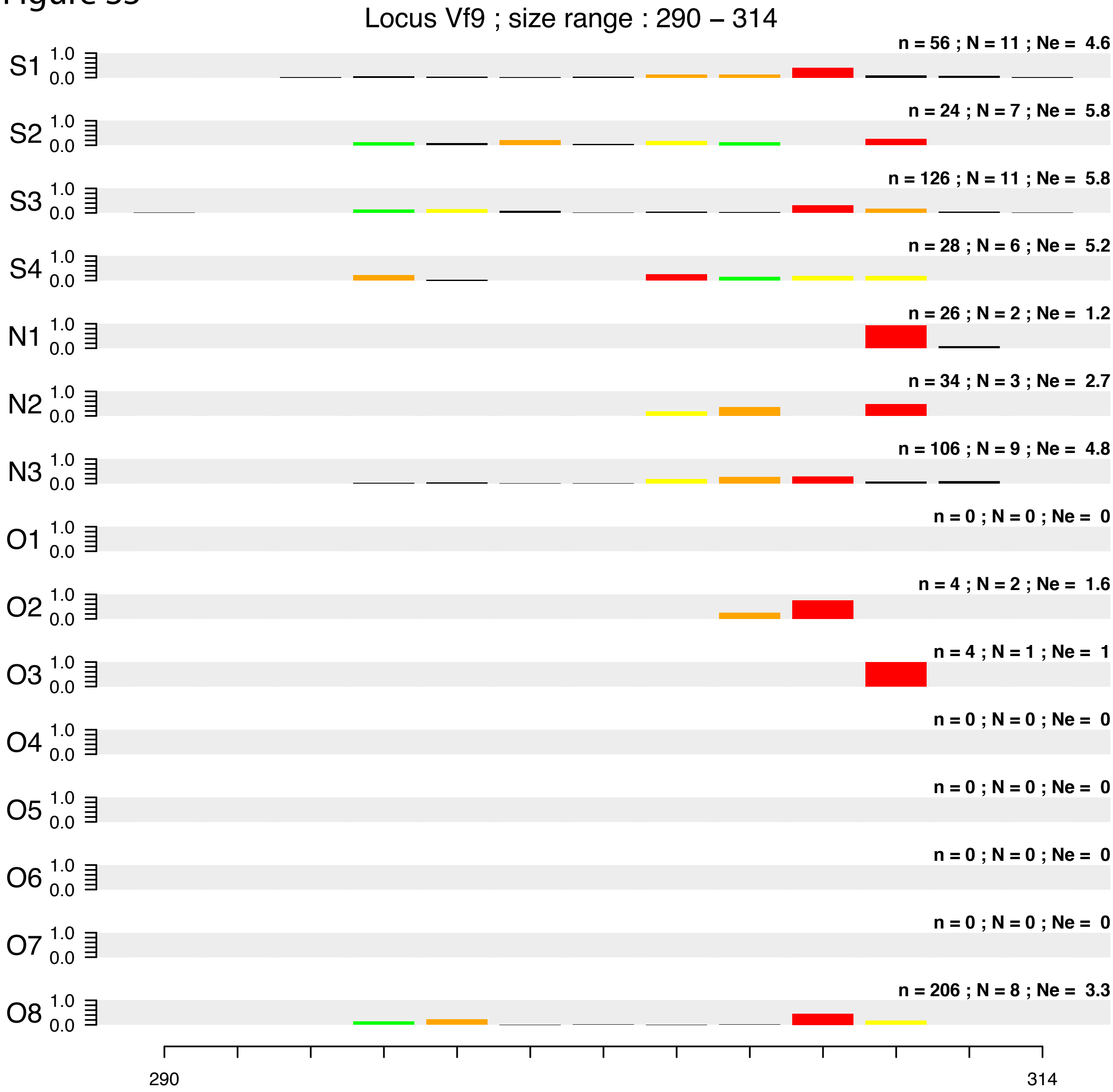

Locus Vf9 ; size range : 290 – 314
S1
n = 56 ; N = 11 ; Ne = 4.6
S2
n = 24 ; N = 7 ; Ne = 5.8
S3
n = 126 ; N = 11 ; Ne = 5.8
S4
n = 28 ; N = 6 ; Ne = 5.2
N1
n = 26 ; N = 2 ; Ne = 1.2
N2
n = 34 ; N = 3 ; Ne = 2.7
N3
n = 106 ; N = 9 ; Ne = 4.8
O1
n = 0 ; N = 0 ; Ne = 0
O2
n = 4 ; N = 2 ; Ne = 1.6
O3
n = 4 ; N = 1 ; Ne = 1
O4
n = 0 ; N = 0 ; Ne = 0
O5
n = 0 ; N = 0 ; Ne = 0
O6
n = 0 ; N = 0 ; Ne = 0
O7
n = 0 ; N = 0 ; Ne = 0
O8
n = 206 ; N = 8 ; Ne = 3.3
1.0
0.0
290
314

Figure S4

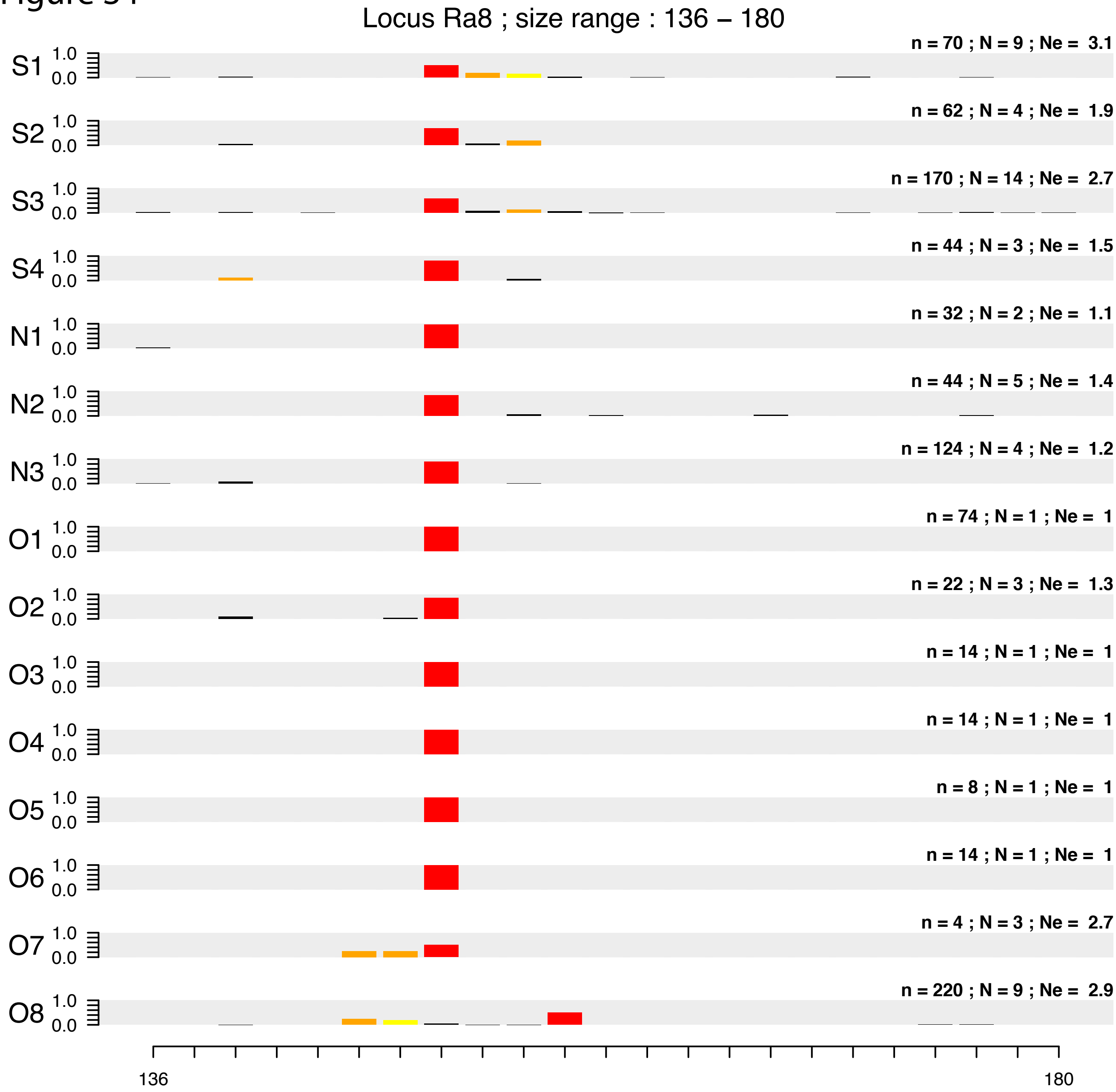
Locus Ra8 ; size range : 136 – 180
S1
n = 70 ; N = 9 ; Ne = 3.1
S2
n = 62 ; N = 4 ; Ne = 1.9
S3
n = 170 ; N = 14 ; Ne = 2.7
S4
n = 44 ; N = 3 ; Ne = 1.5
N1
n = 32 ; N = 2 ; Ne = 1.1
N2
n = 44 ; N = 5 ; Ne = 1.4
N3
n = 124 ; N = 4 ; Ne = 1.2
O1
n = 74 ; N = 1 ; Ne = 1
O2
n = 22 ; N = 3 ; Ne = 1.3
O3
n = 14 ; N = 1 ; Ne = 1
O4
n = 14 ; N = 1 ; Ne = 1
O5
n = 8 ; N = 1 ; Ne = 1
O6
n = 14 ; N = 1 ; Ne = 1
O7
n = 4 ; N = 3 ; Ne = 2.7
O8
n = 220 ; N = 9 ; Ne = 2.9
1.0
0.0
136
180

Figure S5

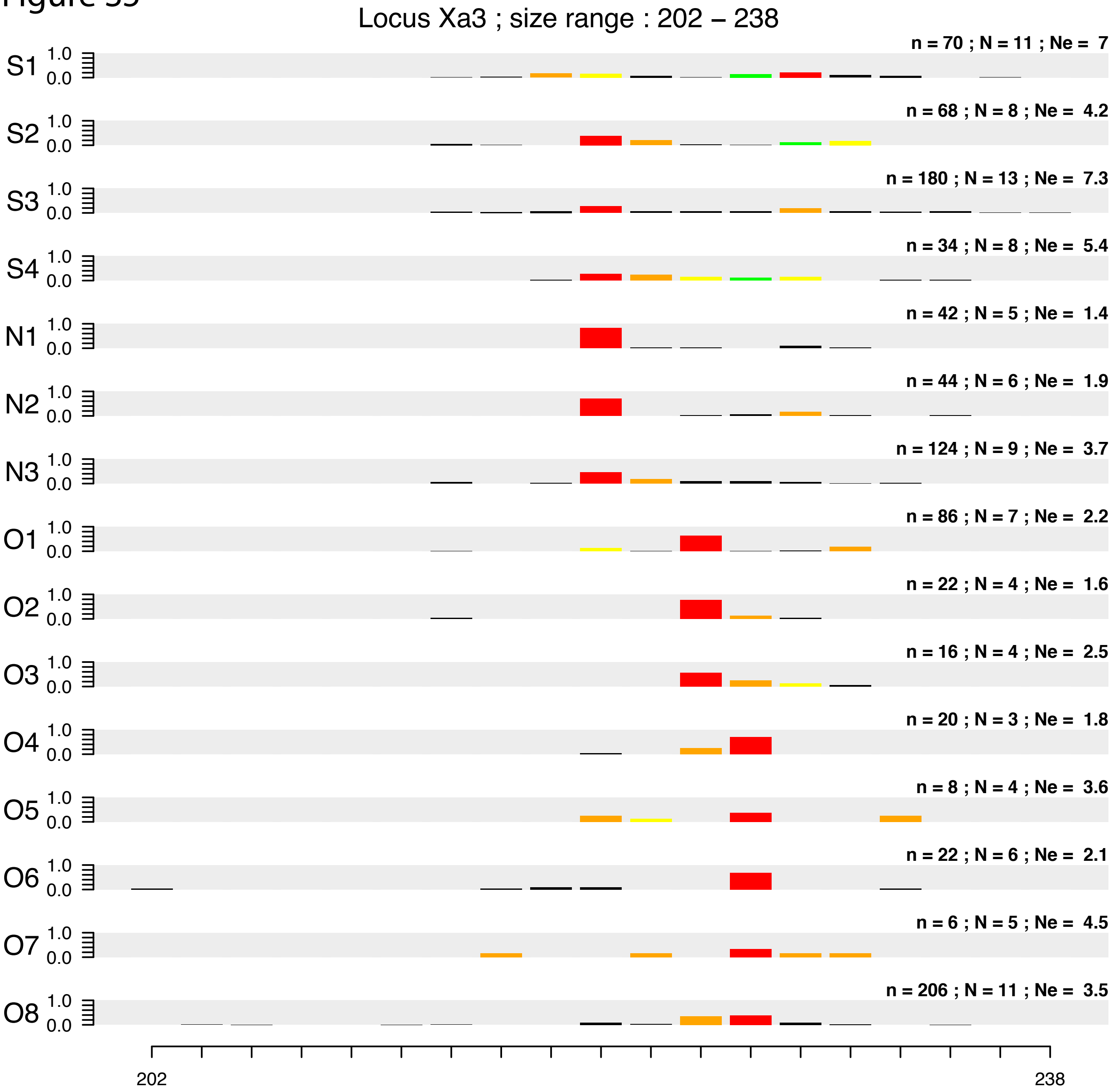

Locus Xa3 ; size range : 202 – 238
S1
n = 70 ; N = 11 ; Ne = 7
S2
n = 68 ; N = 8 ; Ne = 4.2
S3
n = 180 ; N = 13 ; Ne = 7.3
S4
n = 34 ; N = 8 ; Ne = 5.4
N1
n = 42 ; N = 5 ; Ne = 1.4
N2
n = 44 ; N = 6 ; Ne = 1.9
N3
n = 124 ; N = 9 ; Ne = 3.7
O1
n = 86 ; N = 7 ; Ne = 2.2
O2
n = 22 ; N = 4 ; Ne = 1.6
O3
n = 16 ; N = 4 ; Ne = 2.5
O4
n = 20 ; N = 3 ; Ne = 1.8
O5
n = 8 ; N = 4 ; Ne = 3.6
O6
n = 22 ; N = 6 ; Ne = 2.1
O7
n = 6 ; N = 5 ; Ne = 4.5
O8
n = 206 ; N = 11 ; Ne = 3.5
1.0
0.0
202
238

Figure S6

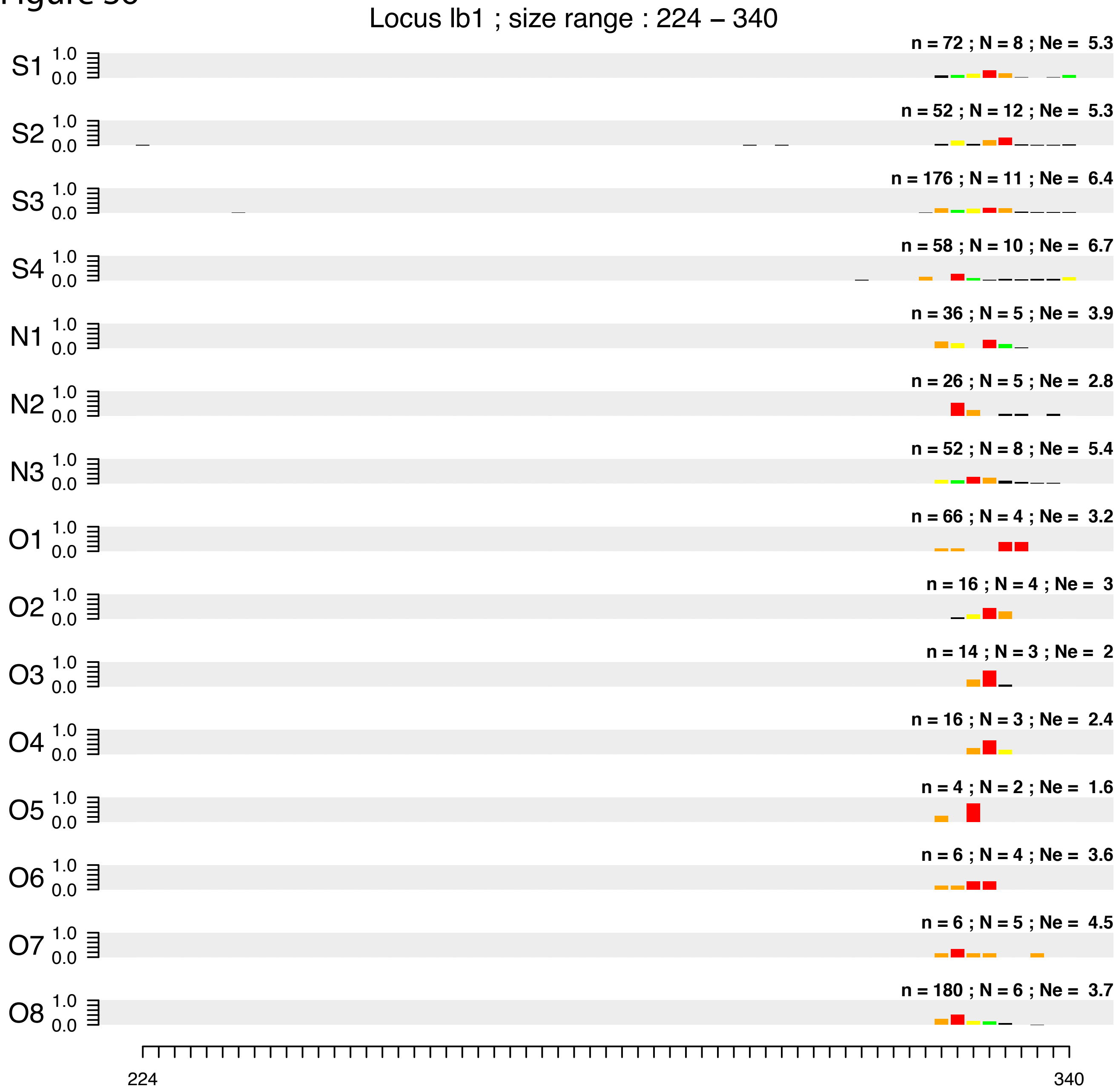
Locus lb1 ; size range : 224 – 340
S1
n = 72 ; N = 8 ; Ne = 5.3
S2
n = 52 ; N = 12 ; Ne = 5.3
S3
n = 176 ; N = 11 ; Ne = 6.4
S4
n = 58 ; N = 10 ; Ne = 6.7
N1
n = 36 ; N = 5 ; Ne = 3.9
N2
n = 26 ; N = 5 ; Ne = 2.8
N3
n = 52 ; N = 8 ; Ne = 5.4
O1
n = 66 ; N = 4 ; Ne = 3.2
O2
n = 16 ; N = 4 ; Ne = 3
O3
n = 14 ; N = 3 ; Ne = 2
O4
n = 16 ; N = 3 ; Ne = 2.4
O5
n = 4 ; N = 2 ; Ne = 1.6
O6
n = 6 ; N = 4 ; Ne = 3.6
O7
n = 6 ; N = 5 ; Ne = 4.5
O8
n = 180 ; N = 6 ; Ne = 3.7
1.0
0.0
224
340

Figure S7

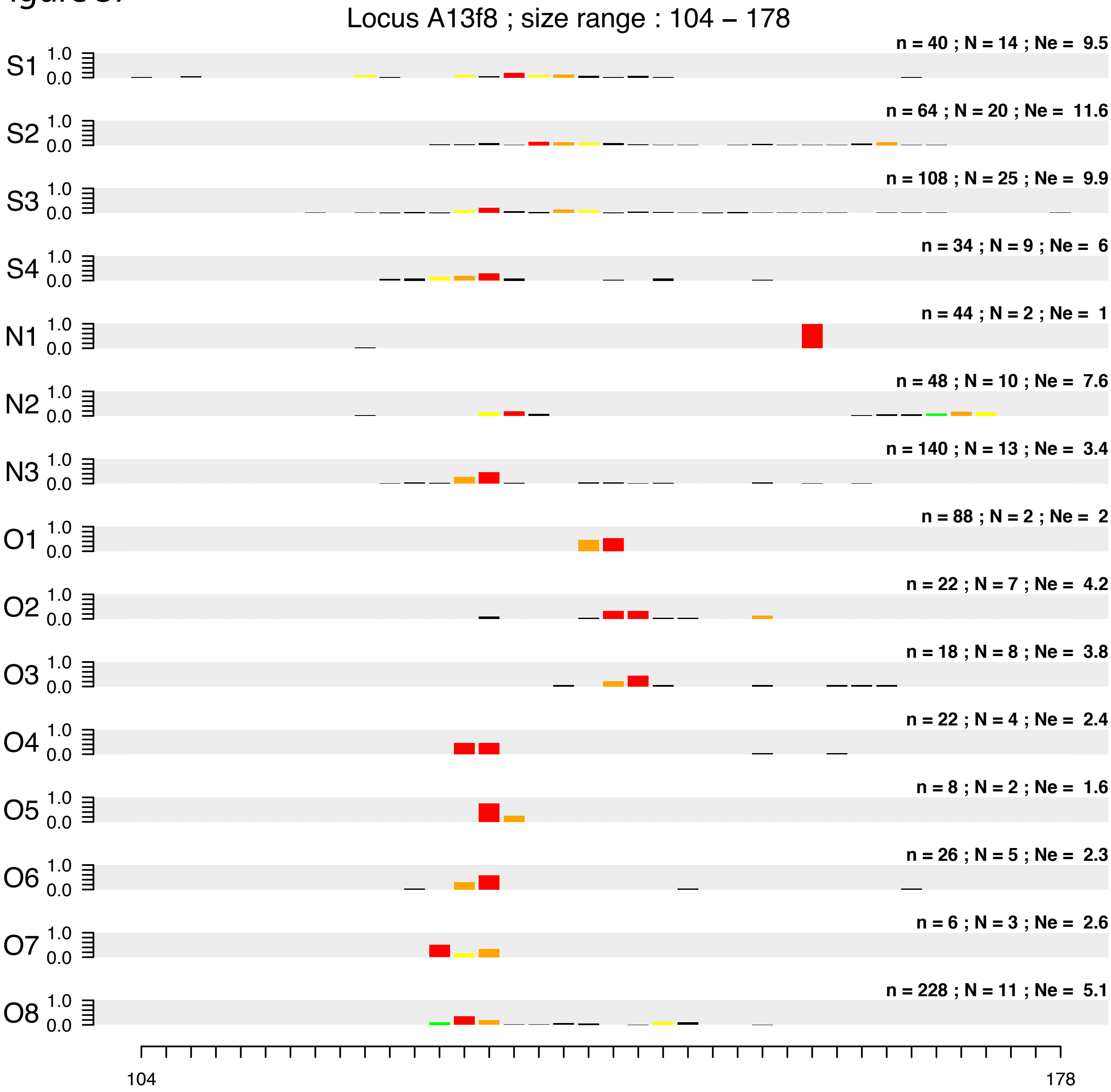
Locus A13f8 ; size range : 104 – 178
S1
n = 40 ; N = 14 ; Ne = 9.5
S2
n = 64 ; N = 20 ; Ne = 11.6
S3
n = 108 ; N = 25 ; Ne = 9.9
S4
n = 34 ; N = 9 ; Ne = 6
N1
n = 44 ; N = 2 ; Ne = 1
N2
n = 48 ; N = 10 ; Ne = 7.6
N3
n = 140 ; N = 13 ; Ne = 3.4
O1
n = 88 ; N = 2 ; Ne = 2
O2
n = 22 ; N = 7 ; Ne = 4.2
O3
n = 18 ; N = 8 ; Ne = 3.8
O4
n = 22 ; N = 4 ; Ne = 2.4
O5
n = 8 ; N = 2 ; Ne = 1.6
O6
n = 26 ; N = 5 ; Ne = 2.3
O7
n = 6 ; N = 3 ; Ne = 2.6
O8
n = 228 ; N = 11 ; Ne = 5.1
1.0
0.0
104
178

Figure S8

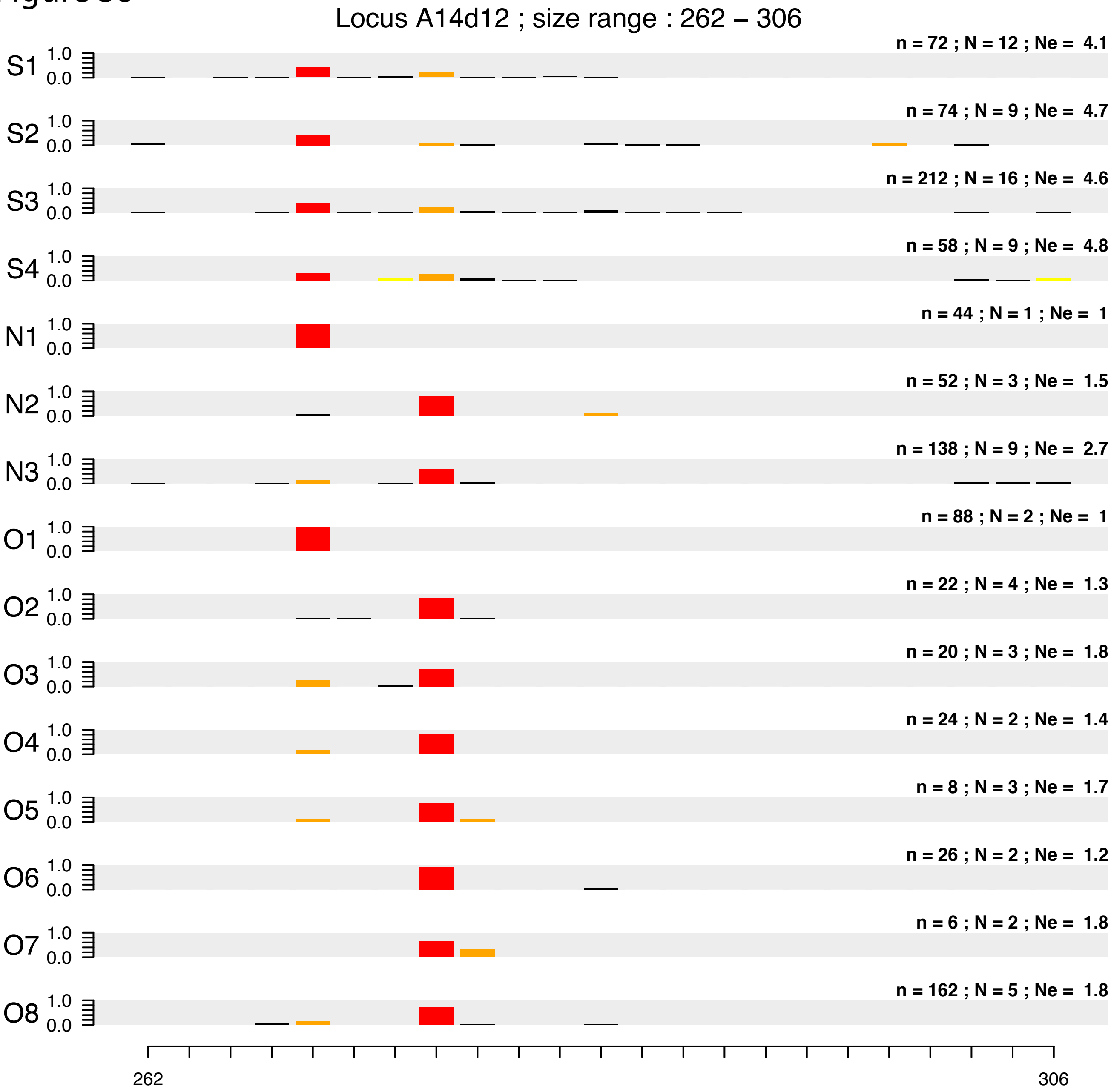
Locus A14d12 ; size range : 262 – 306
S1
n = 72 ; N = 12 ; Ne = 4.1
S2
n = 74 ; N = 9 ; Ne = 4.7
S3
n = 212 ; N = 16 ; Ne = 4.6
S4
n = 58 ; N = 9 ; Ne = 4.8
N1
n = 44 ; N = 1 ; Ne = 1
N2
n = 52 ; N = 3 ; Ne = 1.5
N3
n = 138 ; N = 9 ; Ne = 2.7
O1
n = 88 ; N = 2 ; Ne = 1
O2
n = 22 ; N = 4 ; Ne = 1.3
O3
n = 20 ; N = 3 ; Ne = 1.8
O4
n = 24 ; N = 2 ; Ne = 1.4
O5
n = 8 ; N = 3 ; Ne = 1.7
O6
n = 26 ; N = 2 ; Ne = 1.2
O7
n = 6 ; N = 2 ; Ne = 1.8
O8
n = 162 ; N = 5 ; Ne = 1.8
1.0
0.0
262
306

Figure S9

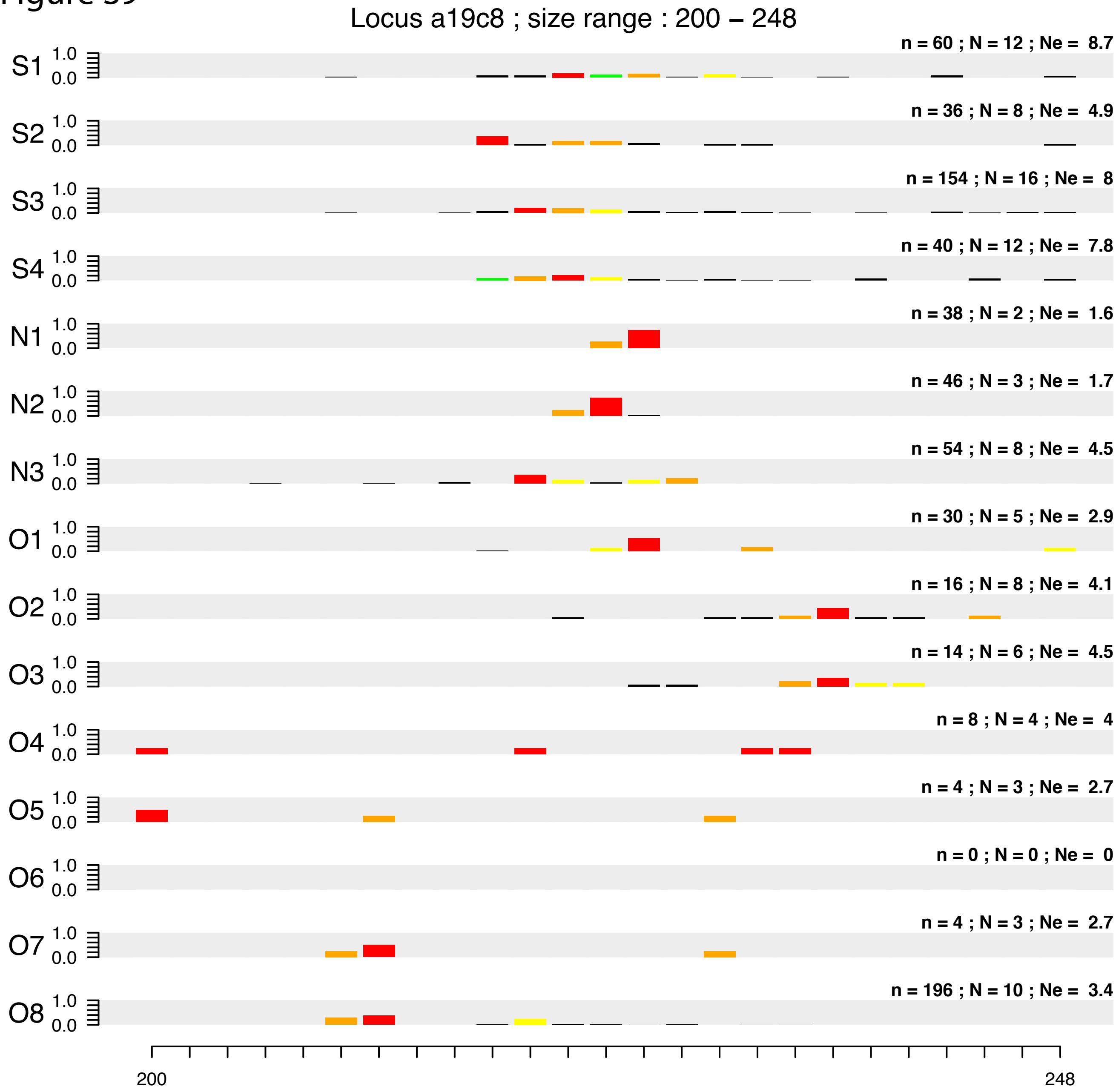
Locus a19c8 ; size range : 200 – 248
S1
n = 60 ; N = 12 ; Ne = 8.7
S2
n = 36 ; N = 8 ; Ne = 4.9
S3
n = 154 ; N = 16 ; Ne = 8
S4
n = 40 ; N = 12 ; Ne = 7.8
N1
n = 38 ; N = 2 ; Ne = 1.6
N2
n = 46 ; N = 3 ; Ne = 1.7
N3
n = 54 ; N = 8 ; Ne = 4.5
O1
n = 30 ; N = 5 ; Ne = 2.9
O2
n = 16 ; N = 8 ; Ne = 4.1
O3
n = 14 ; N = 6 ; Ne = 4.5
O4
n = 8 ; N = 4 ; Ne = 4
O5
n = 4 ; N = 3 ; Ne = 2.7
O6
n = 0 ; N = 0 ; Ne = 0
O7
n = 4 ; N = 3 ; Ne = 2.7
O8
n = 196 ; N = 10 ; Ne = 3.4
1.0
0.0
200
248

Figure S10

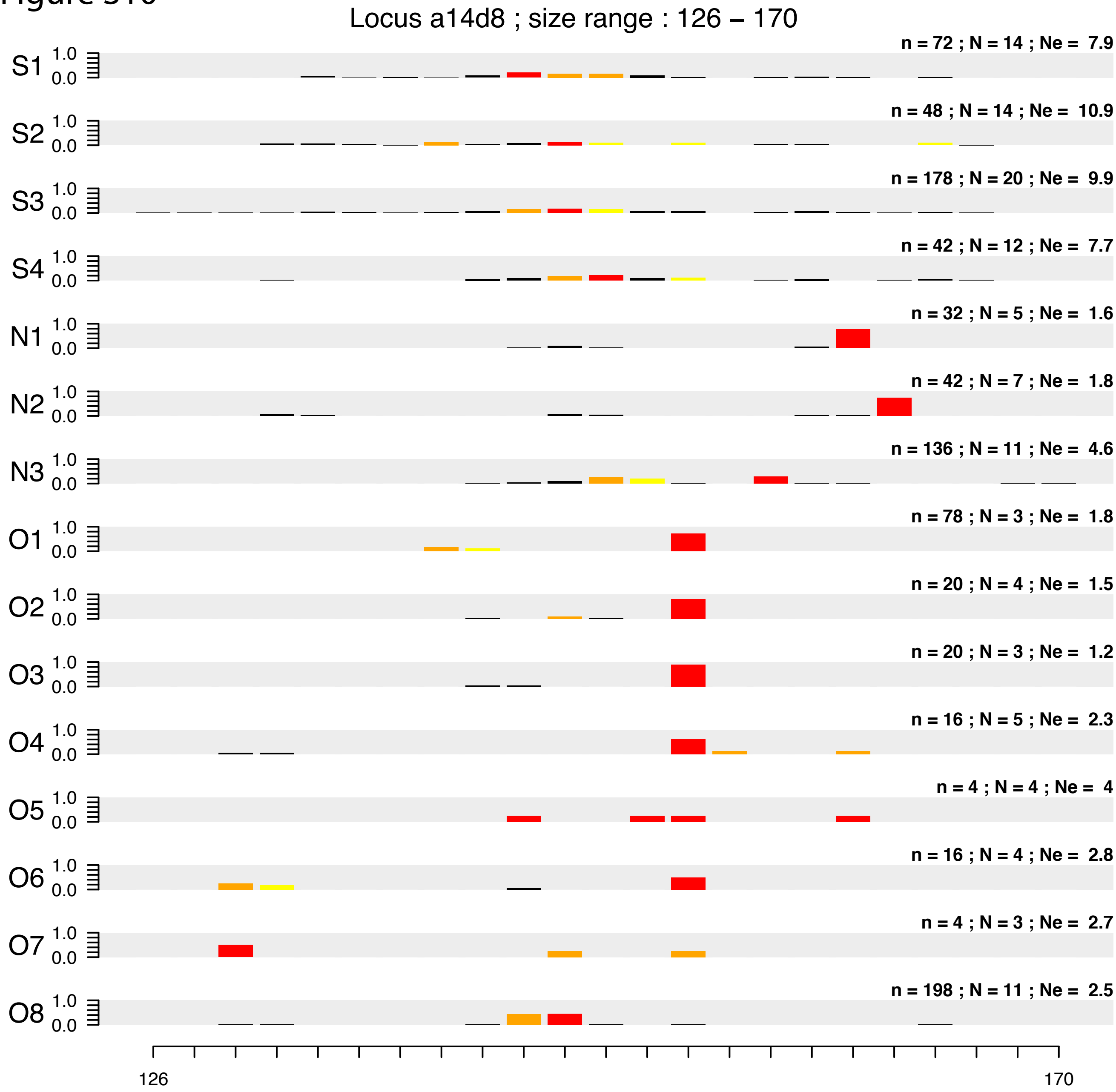

Locus a14d8 ; size range : 126 – 170
S1
n = 72 ; N = 14 ; Ne = 7.9
S2
n = 48 ; N = 14 ; Ne = 10.9
S3
n = 178 ; N = 20 ; Ne = 9.9
S4
n = 42 ; N = 12 ; Ne = 7.7
N1
n = 32 ; N = 5 ; Ne = 1.6
N2
n = 42 ; N = 7 ; Ne = 1.8
N3
n = 136 ; N = 11 ; Ne = 4.6
O1
n = 78 ; N = 3 ; Ne = 1.8
O2
n = 20 ; N = 4 ; Ne = 1.5
O3
n = 20 ; N = 3 ; Ne = 1.2
O4
n = 16 ; N = 5 ; Ne = 2.3
O5
n = 4 ; N = 4 ; Ne = 4
O6
n = 16 ; N = 4 ; Ne = 2.8
O7
n = 4 ; N = 3 ; Ne = 2.7
O8
n = 198 ; N = 11 ; Ne = 2.5
1.0
0.0
126
170

Figure S11

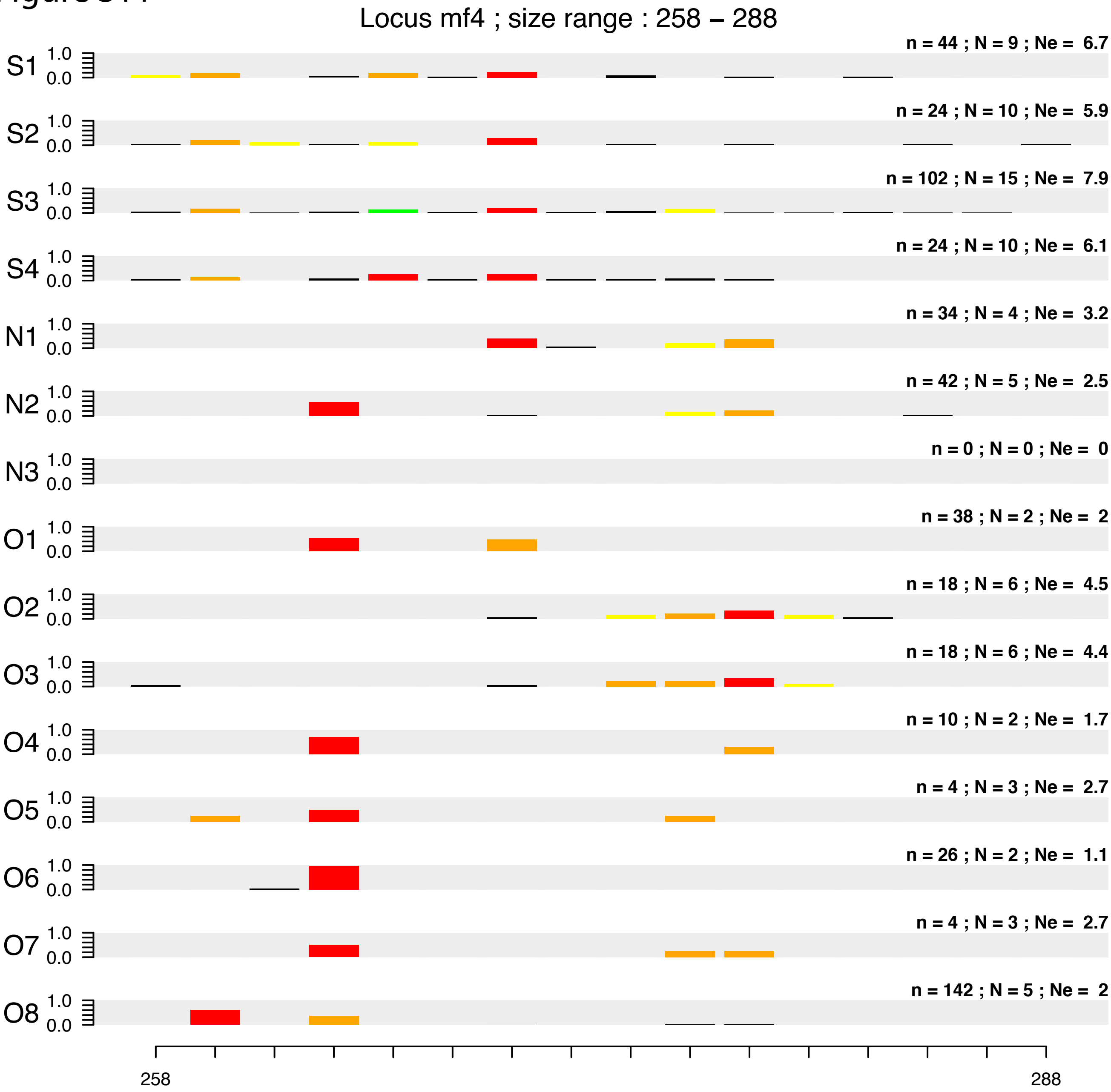

Locus mf4 ; size range : 258 – 288
S1
n = 44 ; N = 9 ; Ne = 6.7
S2
n = 24 ; N = 10 ; Ne = 5.9
S3
n = 102 ; N = 15 ; Ne = 7.9
S4
n = 24 ; N = 10 ; Ne = 6.1
N1
n = 34 ; N = 4 ; Ne = 3.2
N2
n = 42 ; N = 5 ; Ne = 2.5
N3
n = 0 ; N = 0 ; Ne = 0
O1
n = 38 ; N = 2 ; Ne = 2
O2
n = 18 ; N = 6 ; Ne = 4.5
O3
n = 18 ; N = 6 ; Ne = 4.4
O4
n = 10 ; N = 2 ; Ne = 1.7
O5
n = 4 ; N = 3 ; Ne = 2.7
O6
n = 26 ; N = 2 ; Ne = 1.1
O7
n = 4 ; N = 3 ; Ne = 2.7
O8
n = 142 ; N = 5 ; Ne = 2
1.0
0.0
258
288

Figure S12

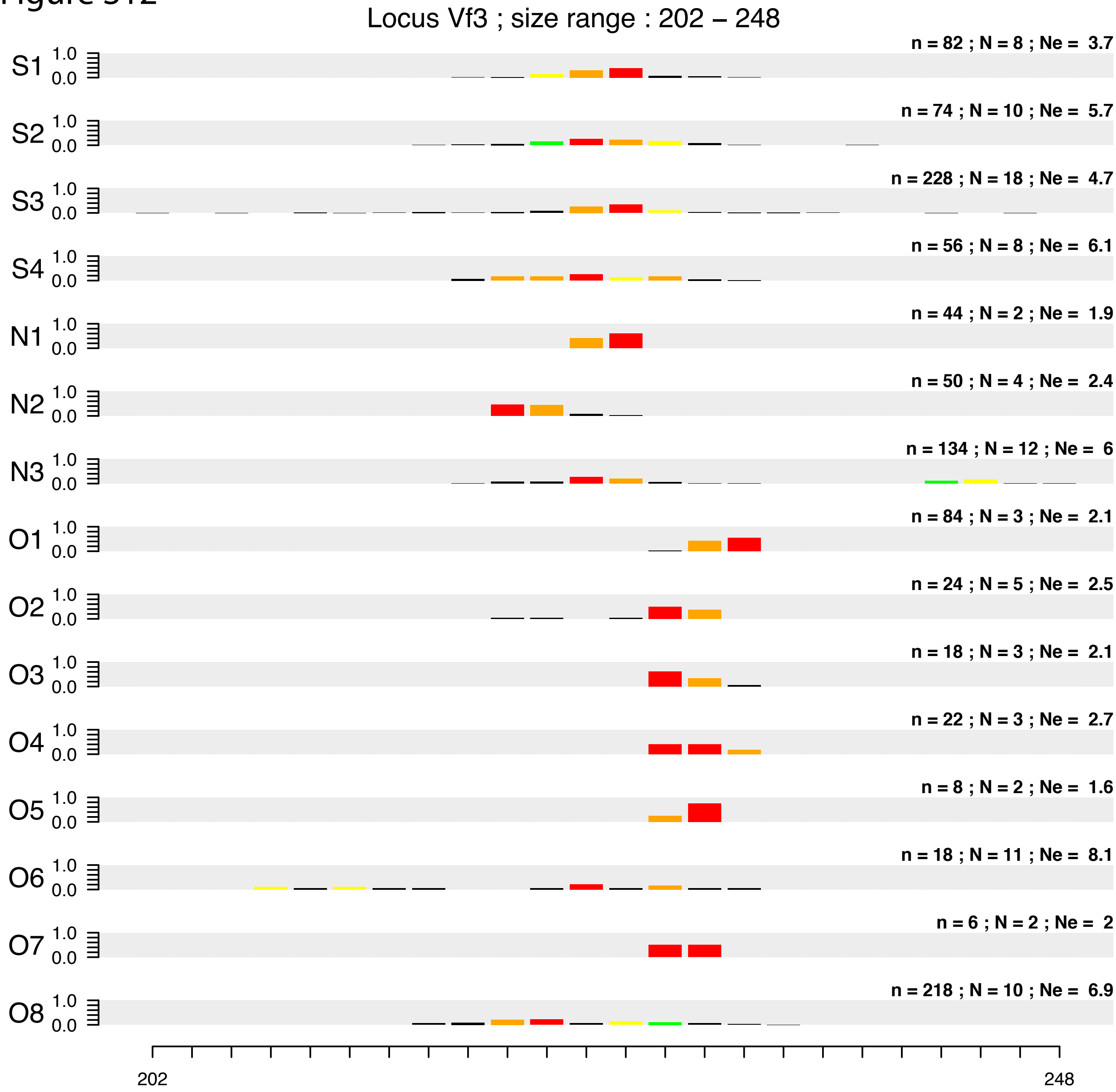
Locus Vf3 ; size range : 202 – 248
S1
n = 82 ; N = 8 ; Ne = 3.7
S2
n = 74 ; N = 10 ; Ne = 5.7
S3
n = 228 ; N = 18 ; Ne = 4.7
S4
n = 56 ; N = 8 ; Ne = 6.1
N1
n = 44 ; N = 2 ; Ne = 1.9
N2
n = 50 ; N = 4 ; Ne = 2.4
N3
n = 134 ; N = 12 ; Ne = 6
O1
n = 84 ; N = 3 ; Ne = 2.1
O2
n = 24 ; N = 5 ; Ne = 2.5
O3
n = 18 ; N = 3 ; Ne = 2.1
O4
n = 22 ; N = 3 ; Ne = 2.7
O5
n = 8 ; N = 2 ; Ne = 1.6
O6
n = 18 ; N = 11 ; Ne = 8.1
O7
n = 6 ; N = 2 ; Ne = 2
O8
n = 218 ; N = 10 ; Ne = 6.9
1.0
0.0
202
248

Figure S13

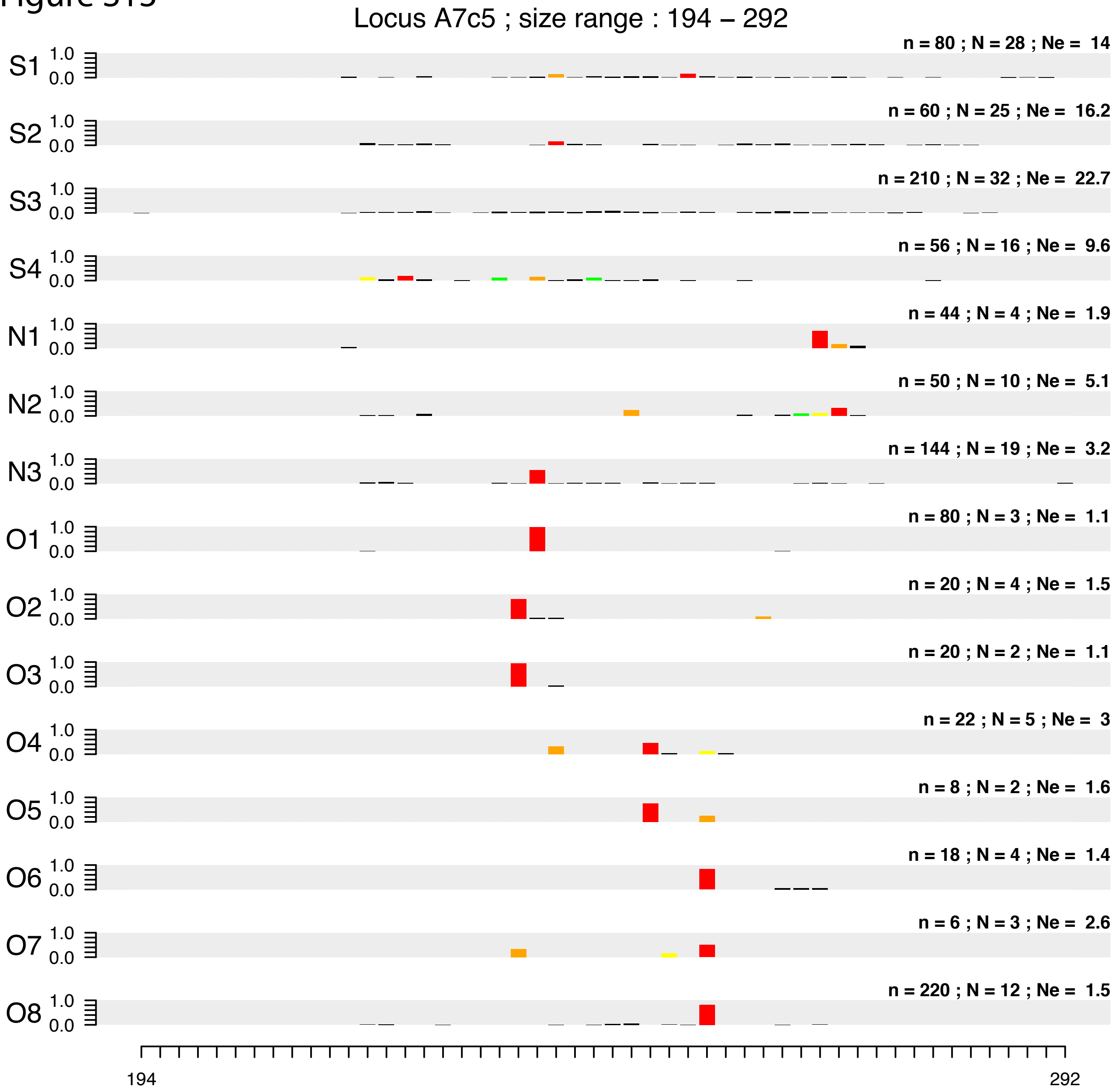
Locus A7c5 ; size range : 194 – 292
S1
n = 80 ; N = 28 ; Ne = 14
S2
n = 60 ; N = 25 ; Ne = 16.2
S3
n = 210 ; N = 32 ; Ne = 22.7
S4
n = 56 ; N = 16 ; Ne = 9.6
N1
n = 44 ; N = 4 ; Ne = 1.9
N2
n = 50 ; N = 10 ; Ne = 5.1
N3
n = 144 ; N = 19 ; Ne = 3.2
O1
n = 80 ; N = 3 ; Ne = 1.1
O2
n = 20 ; N = 4 ; Ne = 1.5
O3
n = 20 ; N = 2 ; Ne = 1.1
O4
n = 22 ; N = 5 ; Ne = 3
O5
n = 8 ; N = 2 ; Ne = 1.6
O6
n = 18 ; N = 4 ; Ne = 1.4
O7
n = 6 ; N = 3 ; Ne = 2.6
O8
n = 220 ; N = 12 ; Ne = 1.5
1.0
0.0
194
292

Figure S14

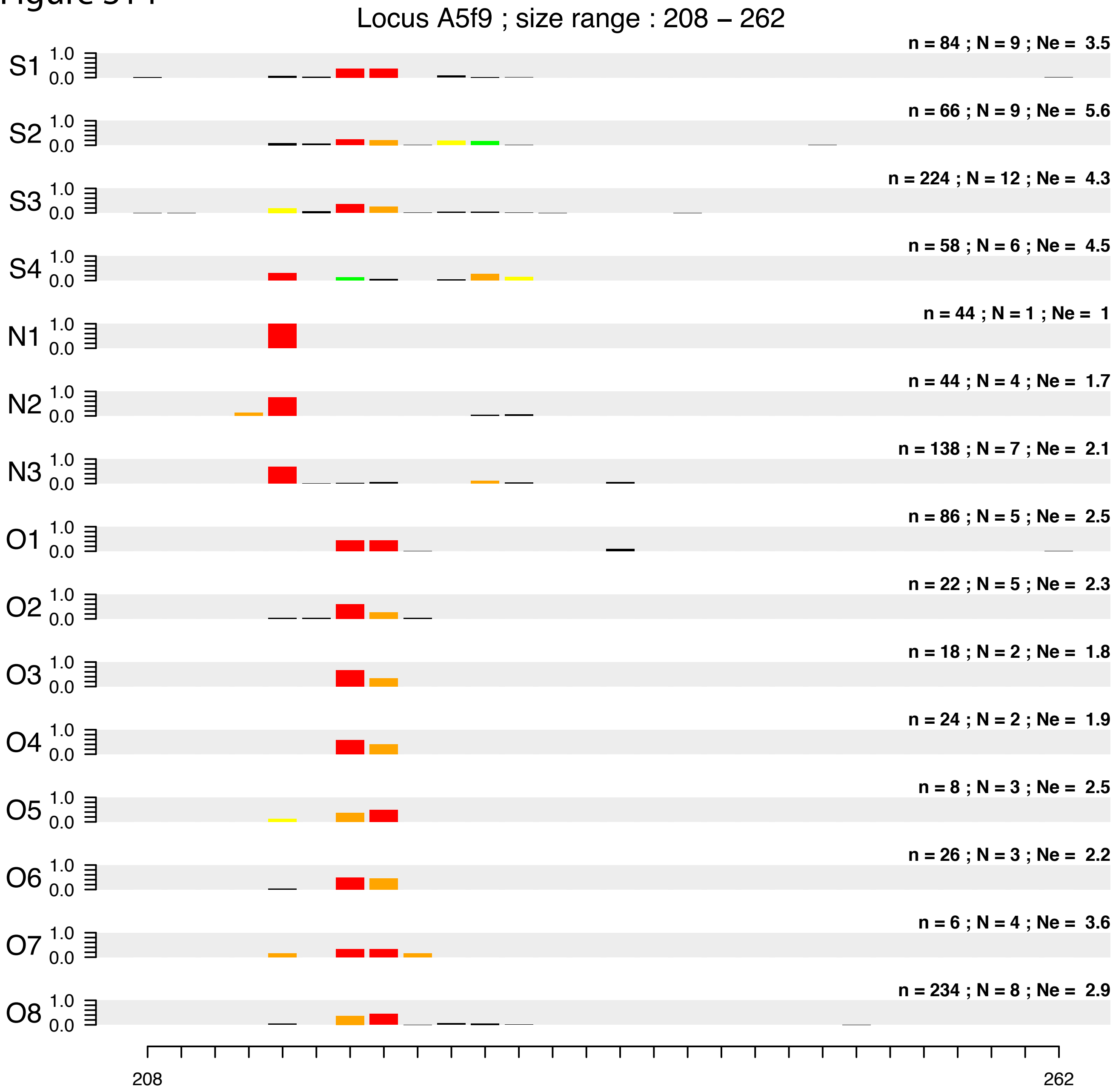
Locus A5f9 ; size range : 208 – 262
S1
n = 84 ; N = 9 ; Ne = 3.5
S2
n = 66 ; N = 9 ; Ne = 5.6
S3
n = 224 ; N = 12 ; Ne = 4.3
S4
n = 58 ; N = 6 ; Ne = 4.5
N1
n = 44 ; N = 1 ; Ne = 1
N2
n = 44 ; N = 4 ; Ne = 1.7
N3
n = 138 ; N = 7 ; Ne = 2.1
O1
n = 86 ; N = 5 ; Ne = 2.5
O2
n = 22 ; N = 5 ; Ne = 2.3
O3
n = 18 ; N = 2 ; Ne = 1.8
O4
n = 24 ; N = 2 ; Ne = 1.9
O5
n = 8 ; N = 3 ; Ne = 2.5
O6
n = 26 ; N = 3 ; Ne = 2.2
O7
n = 6 ; N = 4 ; Ne = 3.6
O8
n = 234 ; N = 8 ; Ne = 2.9
1.0
0.0
208
262

Figure S15

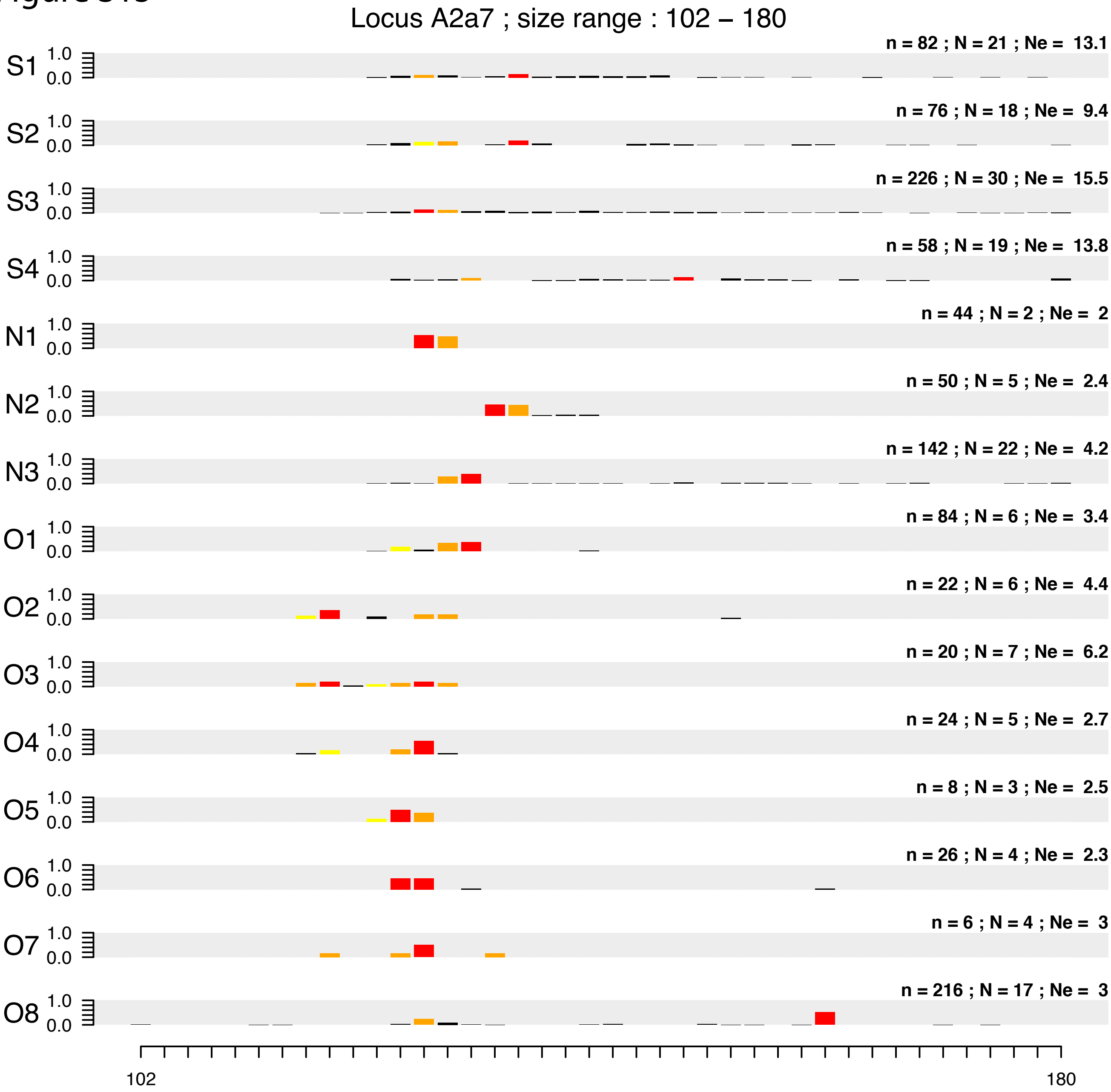

Locus A2a7 ; size range : 102 – 180
S1
n = 82 ; N = 21 ; Ne = 13.1
S2
n = 76 ; N = 18 ; Ne = 9.4
S3
n = 226 ; N = 30 ; Ne = 15.5
S4
n = 58 ; N = 19 ; Ne = 13.8
N1
n = 44 ; N = 2 ; Ne = 2
N2
n = 50 ; N = 5 ; Ne = 2.4
N3
n = 142 ; N = 22 ; Ne = 4.2
O1
n = 84 ; N = 6 ; Ne = 3.4
O2
n = 22 ; N = 6 ; Ne = 4.4
O3
n = 20 ; N = 7 ; Ne = 6.2
O4
n = 24 ; N = 5 ; Ne = 2.7
O5
n = 8 ; N = 3 ; Ne = 2.5
O6
n = 26 ; N = 4 ; Ne = 2.3
O7
n = 6 ; N = 4 ; Ne = 3
O8
n = 216 ; N = 17 ; Ne = 3
1.0
0.0
102
180

Figure S16

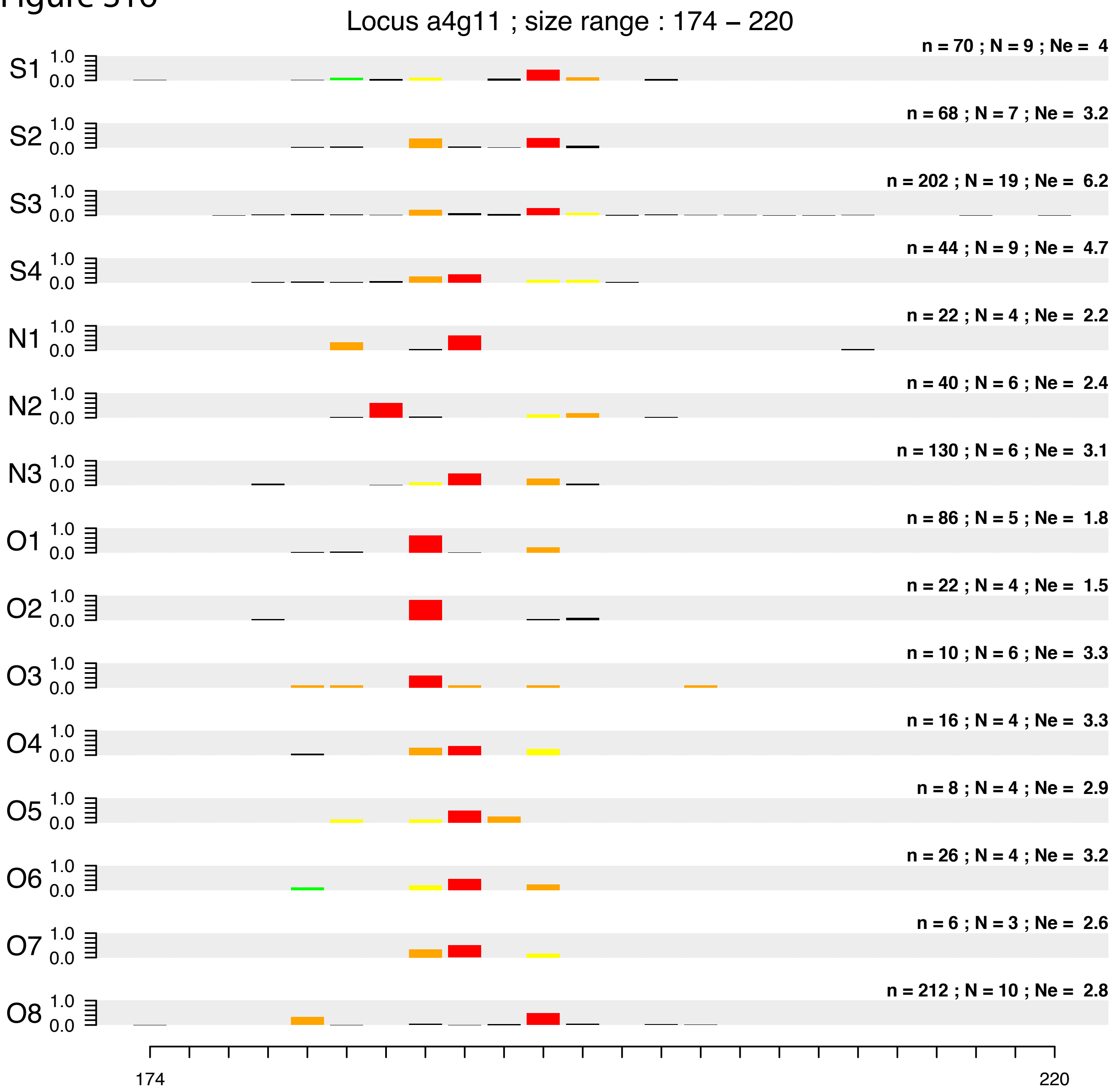
Locus a4g11 ; size range : 174 – 220
S1
n = 70 ; N = 9 ; Ne = 4
S2
n = 68 ; N = 7 ; Ne = 3.2
S3
n = 202 ; N = 19 ; Ne = 6.2
S4
n = 44 ; N = 9 ; Ne = 4.7
N1
n = 22 ; N = 4 ; Ne = 2.2
N2
n = 40 ; N = 6 ; Ne = 2.4
N3
n = 130 ; N = 6 ; Ne = 3.1
O1
n = 86 ; N = 5 ; Ne = 1.8
O2
n = 22 ; N = 4 ; Ne = 1.5
O3
n = 10 ; N = 6 ; Ne = 3.3
O4
n = 16 ; N = 4 ; Ne = 3.3
O5
n = 8 ; N = 4 ; Ne = 2.9
O6
n = 26 ; N = 4 ; Ne = 3.2
O7
n = 6 ; N = 3 ; Ne = 2.6
O8
n = 212 ; N = 10 ; Ne = 2.8
1.0
0.0
174
220

Figure S17

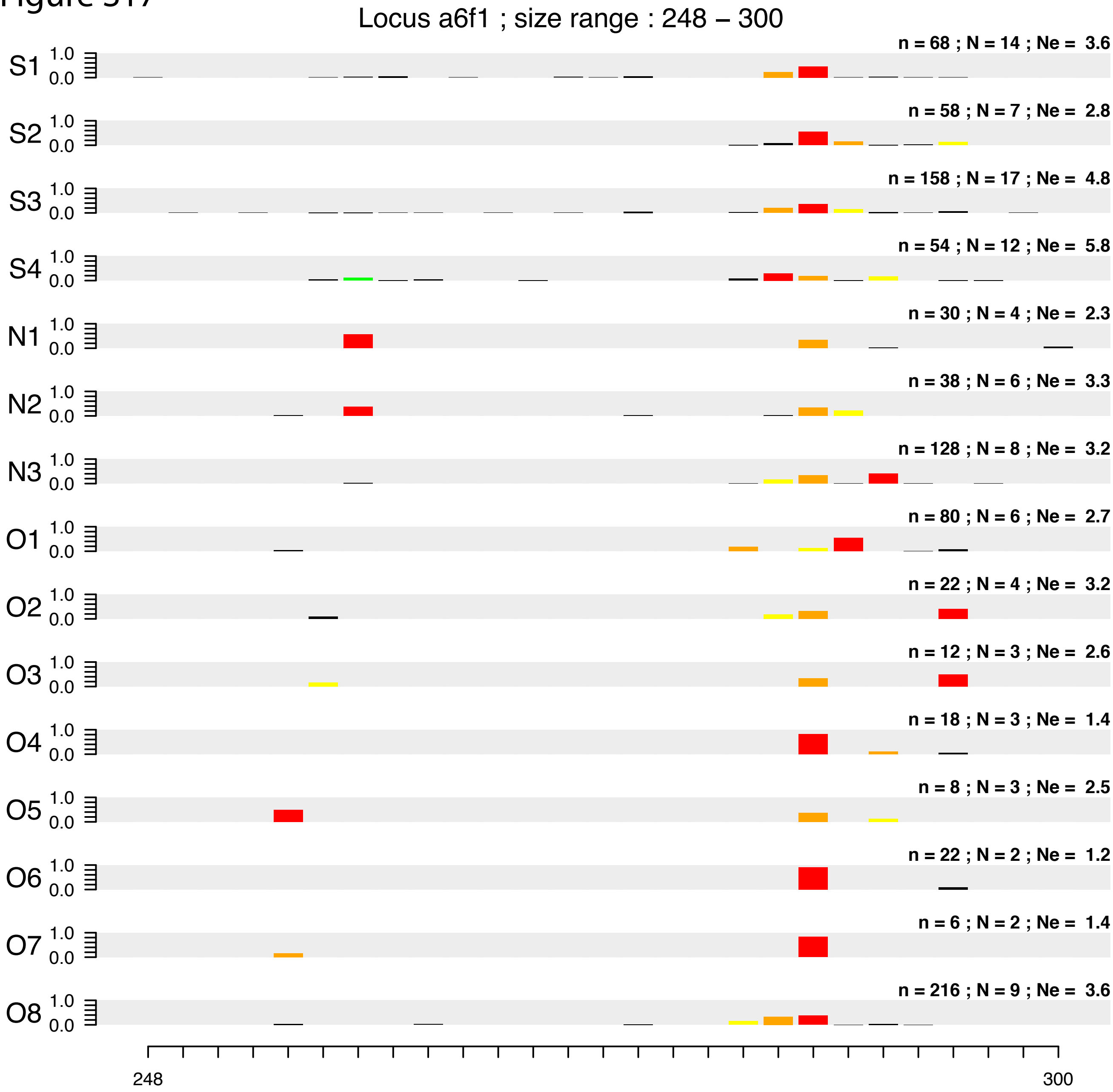
Locus a6f1 ; size range : 248 – 300
S1
n = 68 ; N = 14 ; Ne = 3.6
S2
n = 58 ; N = 7 ; Ne = 2.8
S3
n = 158 ; N = 17 ; Ne = 4.8
S4
n = 54 ; N = 12 ; Ne = 5.8
N1
n = 30 ; N = 4 ; Ne = 2.3
N2
n = 38 ; N = 6 ; Ne = 3.3
N3
n = 128 ; N = 8 ; Ne = 3.2
O1
n = 80 ; N = 6 ; Ne = 2.7
O2
n = 22 ; N = 4 ; Ne = 3.2
O3
n = 12 ; N = 3 ; Ne = 2.6
O4
n = 18 ; N = 3 ; Ne = 1.4
O5
n = 8 ; N = 3 ; Ne = 2.5
O6
n = 22 ; N = 2 ; Ne = 1.2
O7
n = 6 ; N = 2 ; Ne = 1.4
O8
n = 216 ; N = 9 ; Ne = 3.6
1.0
0.0
248
300

Figure S18

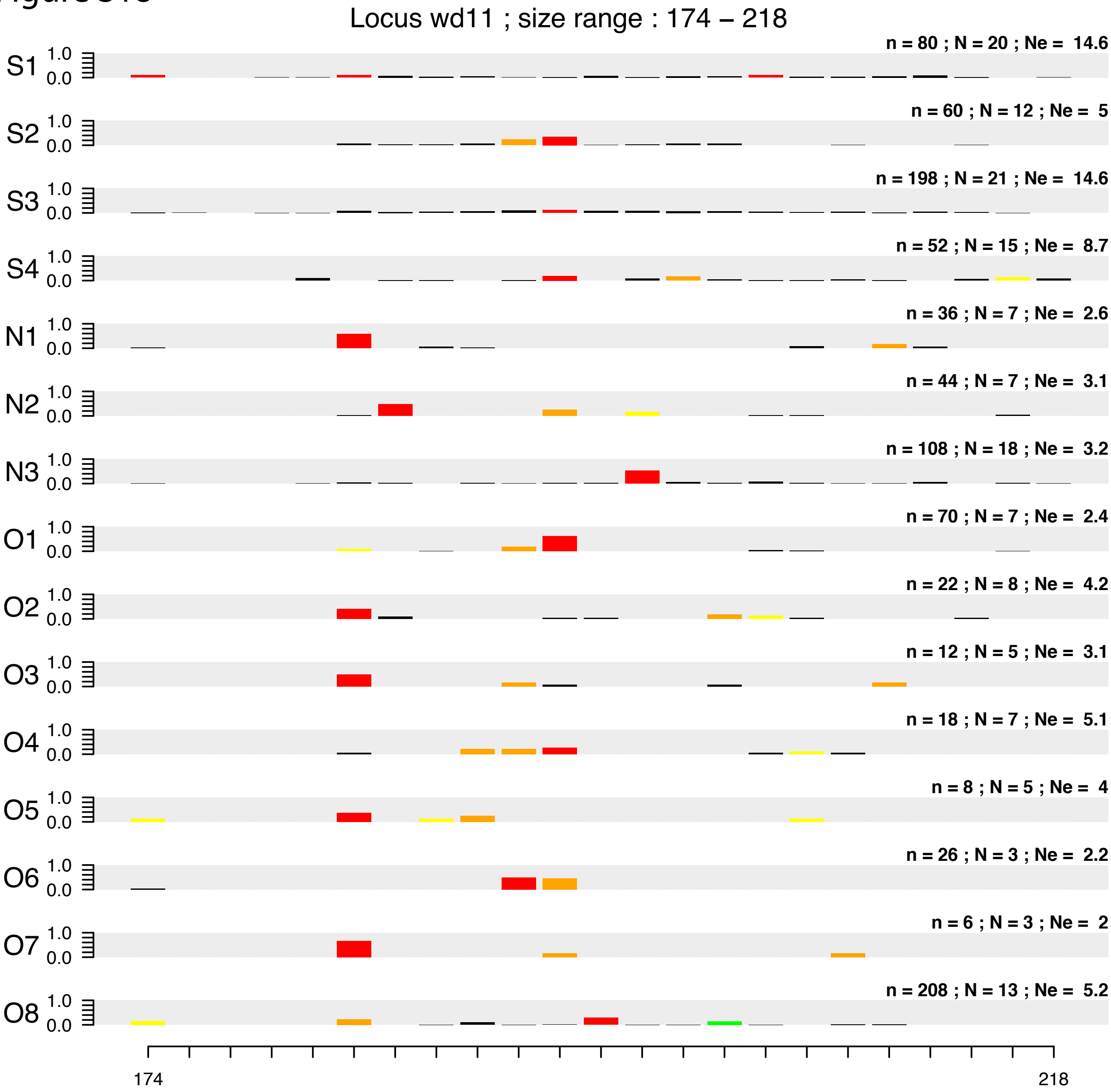

Locus wd11 ; size range : 174 – 218
S1
n = 80 ; N = 20 ; Ne = 14.6
S2
n = 60 ; N = 12 ; Ne = 5
S3
n = 198 ; N = 21 ; Ne = 14.6
S4
n = 52 ; N = 15 ; Ne = 8.7
N1
n = 36 ; N = 7 ; Ne = 2.6
N2
n = 44 ; N = 7 ; Ne = 3.1
N3
n = 108 ; N = 18 ; Ne = 3.2
O1
n = 70 ; N = 7 ; Ne = 2.4
O2
n = 22 ; N = 8 ; Ne = 4.2
O3
n = 12 ; N = 5 ; Ne = 3.1
O4
n = 18 ; N = 7 ; Ne = 5.1
O5
n = 8 ; N = 5 ; Ne = 4
O6
n = 26 ; N = 3 ; Ne = 2.2
O7
n = 6 ; N = 3 ; Ne = 2
O8
n = 208 ; N = 13 ; Ne = 5.2
1.0
0.0
174
218

Figure S19

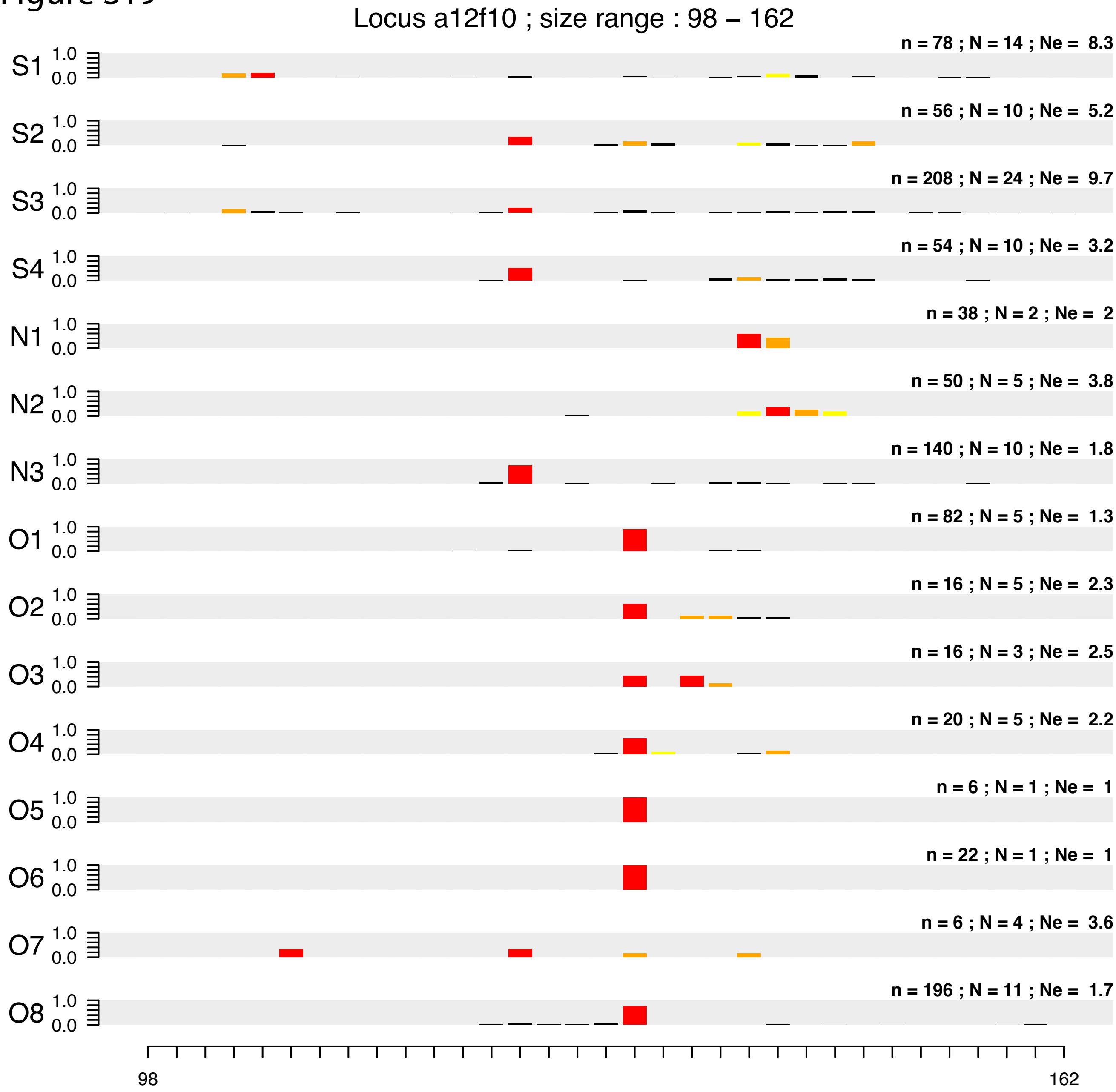
Locus a12f10 ; size range : 98 – 162
S1
n = 78 ; N = 14 ; Ne = 8.3
S2
n = 56 ; N = 10 ; Ne = 5.2
S3
n = 208 ; N = 24 ; Ne = 9.7
S4
n = 54 ; N = 10 ; Ne = 3.2
N1
n = 38 ; N = 2 ; Ne = 2
N2
n = 50 ; N = 5 ; Ne = 3.8
N3
n = 140 ; N = 10 ; Ne = 1.8
O1
n = 82 ; N = 5 ; Ne = 1.3
O2
n = 16 ; N = 5 ; Ne = 2.3
O3
n = 16 ; N = 3 ; Ne = 2.5
O4
n = 20 ; N = 5 ; Ne = 2.2
O5
n = 6 ; N = 1 ; Ne = 1
O6
n = 22 ; N = 1 ; Ne = 1
O7
n = 6 ; N = 4 ; Ne = 3.6
O8
n = 196 ; N = 11 ; Ne = 1.7
1.0
0.0
98
162

Figure S20

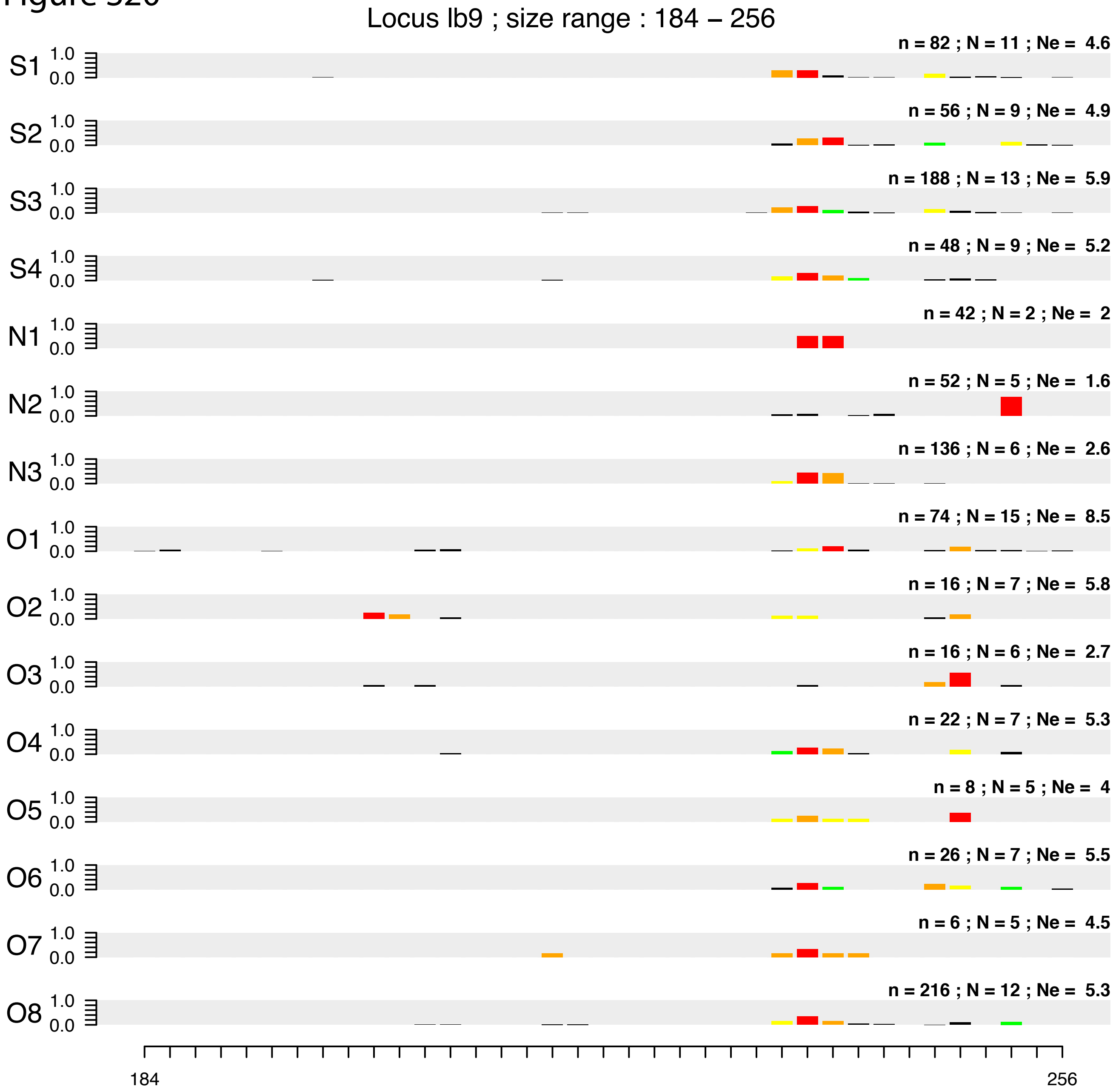
Locus lb9 ; size range : 184 – 256
S1
n = 82 ; N = 11 ; Ne = 4.6
S2
n = 56 ; N = 9 ; Ne = 4.9
S3
n = 188 ; N = 13 ; Ne = 5.9
S4
n = 48 ; N = 9 ; Ne = 5.2
N1
n = 42 ; N = 2 ; Ne = 2
N2
n = 52 ; N = 5 ; Ne = 1.6
N3
n = 136 ; N = 6 ; Ne = 2.6
O1
n = 74 ; N = 15 ; Ne = 8.5
O2
n = 16 ; N = 7 ; Ne = 5.8
O3
n = 16 ; N = 6 ; Ne = 2.7
O4
n = 22 ; N = 7 ; Ne = 5.3
O5
n = 8 ; N = 5 ; Ne = 4
O6
n = 26 ; N = 7 ; Ne = 5.5
O7
n = 6 ; N = 5 ; Ne = 4.5
O8
n = 216 ; N = 12 ; Ne = 5.3
1.0
0.0
184
256

Figure S21

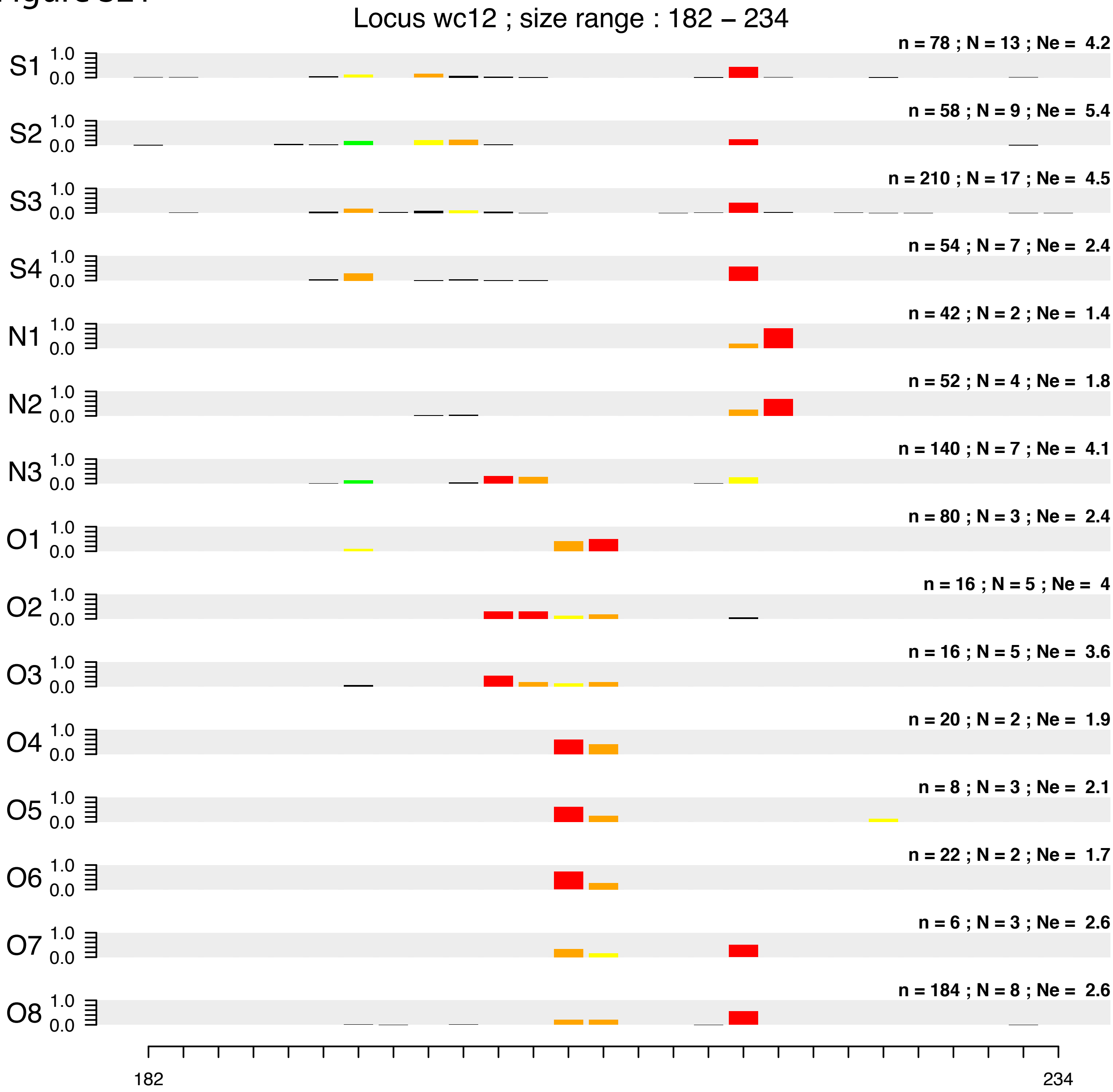

Locus wc12 ; size range : 182 – 234
S1
n = 78 ; N = 13 ; Ne = 4.2
S2
n = 58 ; N = 9 ; Ne = 5.4
S3
n = 210 ; N = 17 ; Ne = 4.5
S4
n = 54 ; N = 7 ; Ne = 2.4
N1
n = 42 ; N = 2 ; Ne = 1.4
N2
n = 52 ; N = 4 ; Ne = 1.8
N3
n = 140 ; N = 7 ; Ne = 4.1
O1
n = 80 ; N = 3 ; Ne = 2.4
O2
n = 16 ; N = 5 ; Ne = 4
O3
n = 16 ; N = 5 ; Ne = 3.6
O4
n = 20 ; N = 2 ; Ne = 1.9
O5
n = 8 ; N = 3 ; Ne = 2.1
O6
n = 22 ; N = 2 ; Ne = 1.7
O7
n = 6 ; N = 3 ; Ne = 2.6
O8
n = 184 ; N = 8 ; Ne = 2.6
1.0
0.0
182
234

Figure S22

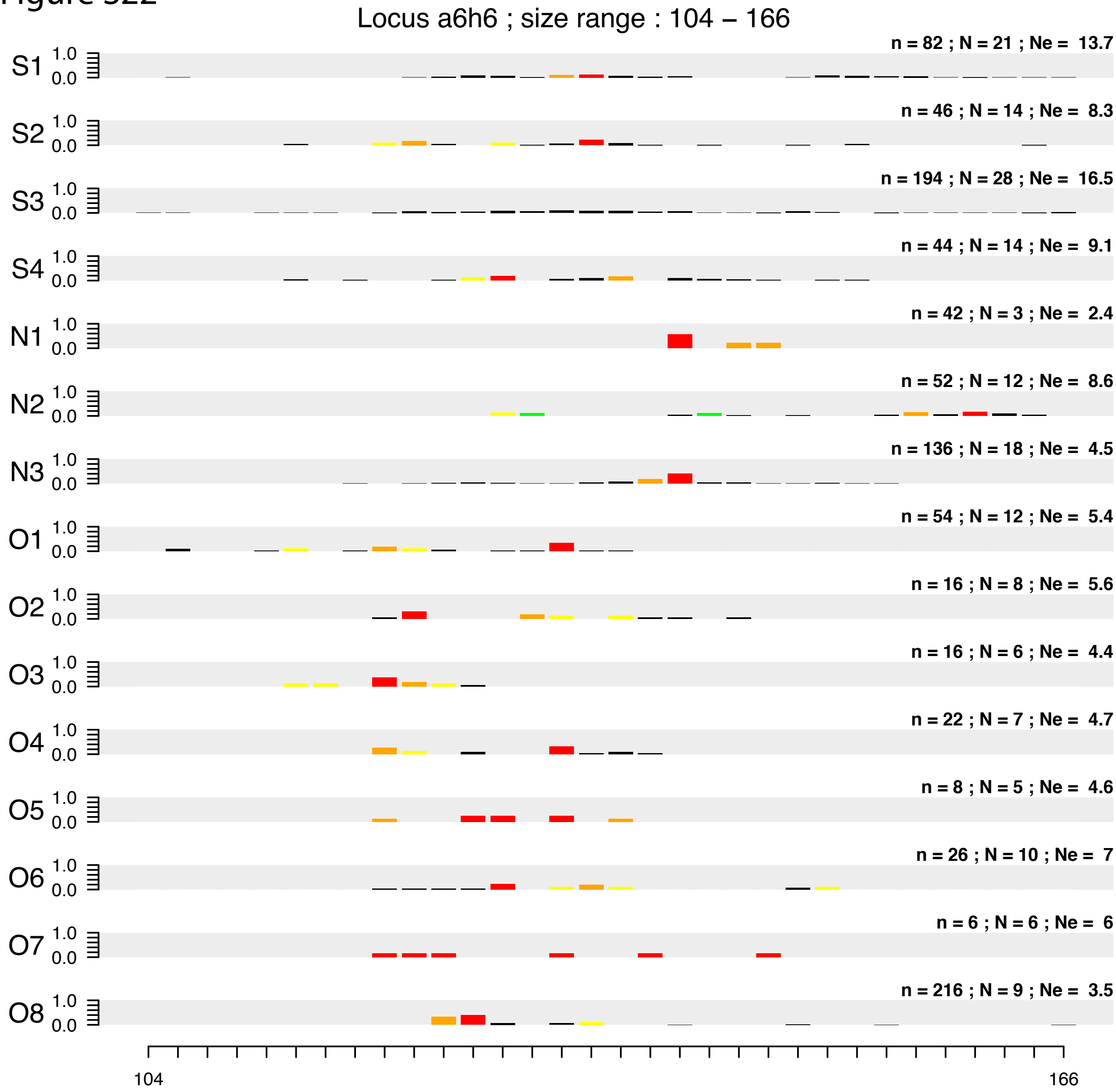

Locus a6h6 ; size range : 104 – 166
S1
n = 82 ; N = 21 ; Ne = 13.7
S2
n = 46 ; N = 14 ; Ne = 8.3
S3
n = 194 ; N = 28 ; Ne = 16.5
S4
n = 44 ; N = 14 ; Ne = 9.1
N1
n = 42 ; N = 3 ; Ne = 2.4
N2
n = 52 ; N = 12 ; Ne = 8.6
N3
n = 136 ; N = 18 ; Ne = 4.5
O1
n = 54 ; N = 12 ; Ne = 5.4
O2
n = 16 ; N = 8 ; Ne = 5.6
O3
n = 16 ; N = 6 ; Ne = 4.4
O4
n = 22 ; N = 7 ; Ne = 4.7
O5
n = 8 ; N = 5 ; Ne = 4.6
O6
n = 26 ; N = 10 ; Ne = 7
O7
n = 6 ; N = 6 ; Ne = 6
O8
n = 216 ; N = 9 ; Ne = 3.5
1.0
0.0
104
166

Figure S23

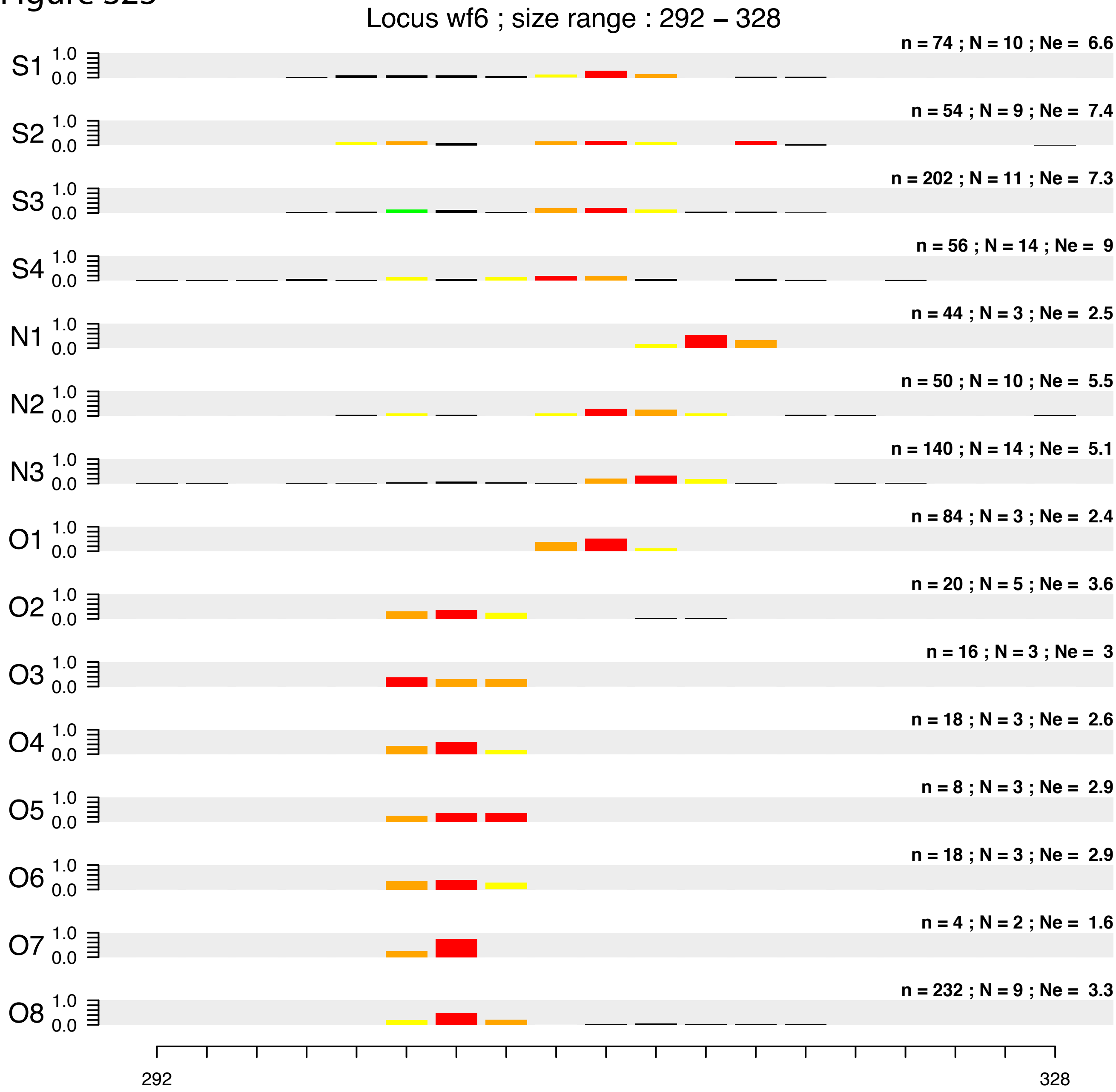

Locus wf6 ; size range : 292 – 328
S1
n = 74 ; N = 10 ; Ne = 6.6
S2
n = 54 ; N = 9 ; Ne = 7.4
S3
n = 202 ; N = 11 ; Ne = 7.3
S4
n = 56 ; N = 14 ; Ne = 9
N1
n = 44 ; N = 3 ; Ne = 2.5
N2
n = 50 ; N = 10 ; Ne = 5.5
N3
n = 140 ; N = 14 ; Ne = 5.1
O1
n = 84 ; N = 3 ; Ne = 2.4
O2
n = 20 ; N = 5 ; Ne = 3.6
O3
n = 16 ; N = 3 ; Ne = 3
O4
n = 18 ; N = 3 ; Ne = 2.6
O5
n = 8 ; N = 3 ; Ne = 2.9
O6
n = 18 ; N = 3 ; Ne = 2.9
O7
n = 4 ; N = 2 ; Ne = 1.6
O8
n = 232 ; N = 9 ; Ne = 3.3
1.0
0.0
292
328

Figure S24

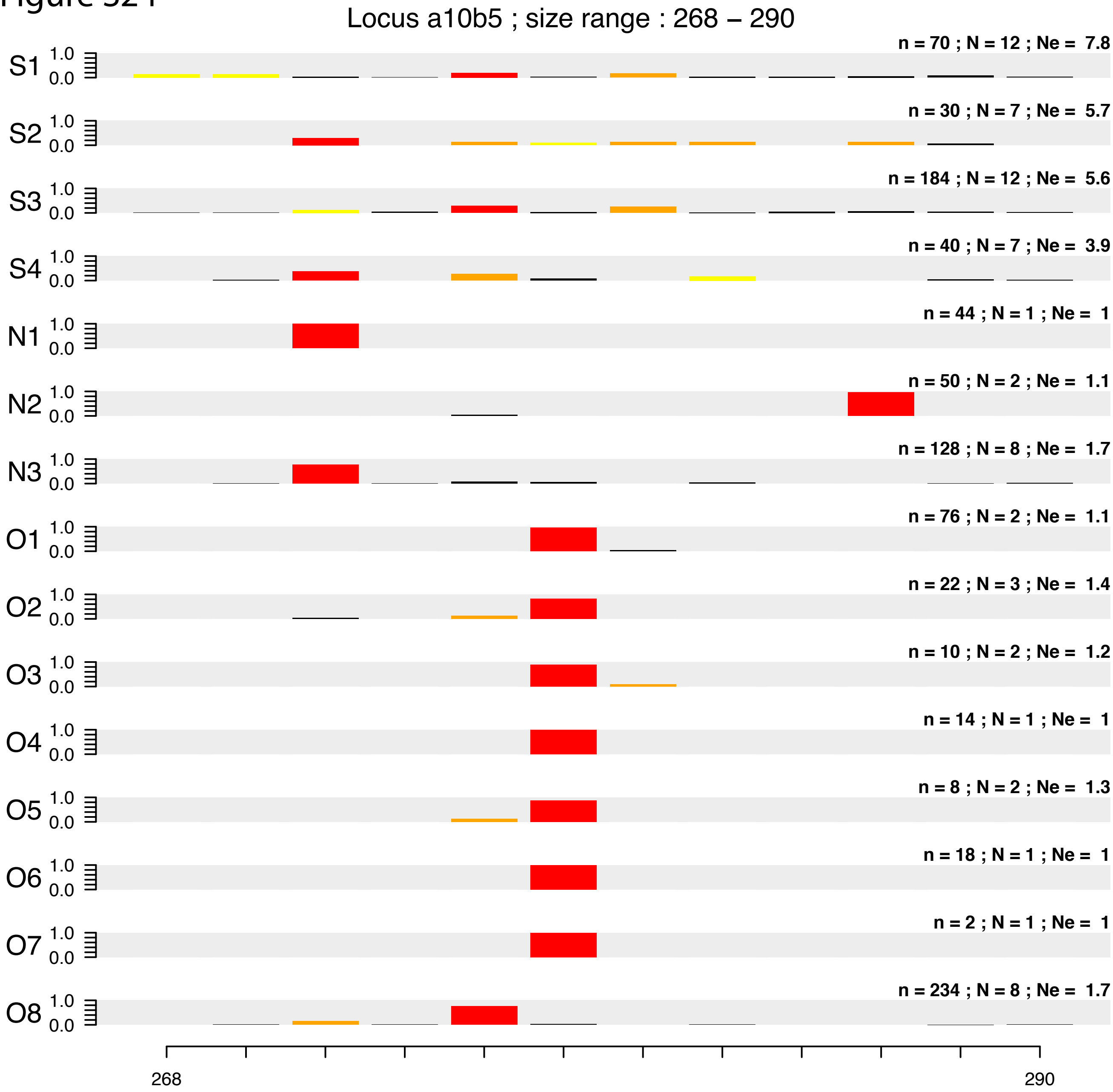

Locus a10b5 ; size range : 268 – 290
S1
n = 70 ; N = 12 ; Ne = 7.8
S2
n = 30 ; N = 7 ; Ne = 5.7
S3
n = 184 ; N = 12 ; Ne = 5.6
S4
n = 40 ; N = 7 ; Ne = 3.9
N1
n = 44 ; N = 1 ; Ne = 1
N2
n = 50 ; N = 2 ; Ne = 1.1
N3
n = 128 ; N = 8 ; Ne = 1.7
O1
n = 76 ; N = 2 ; Ne = 1.1
O2
n = 22 ; N = 3 ; Ne = 1.4
O3
n = 10 ; N = 2 ; Ne = 1.2
O4
n = 14 ; N = 1 ; Ne = 1
O5
n = 8 ; N = 2 ; Ne = 1.3
O6
n = 18 ; N = 1 ; Ne = 1
O7
n = 2 ; N = 1 ; Ne = 1
O8
n = 234 ; N = 8 ; Ne = 1.7
1.0
0.0
268
290

Figure S25

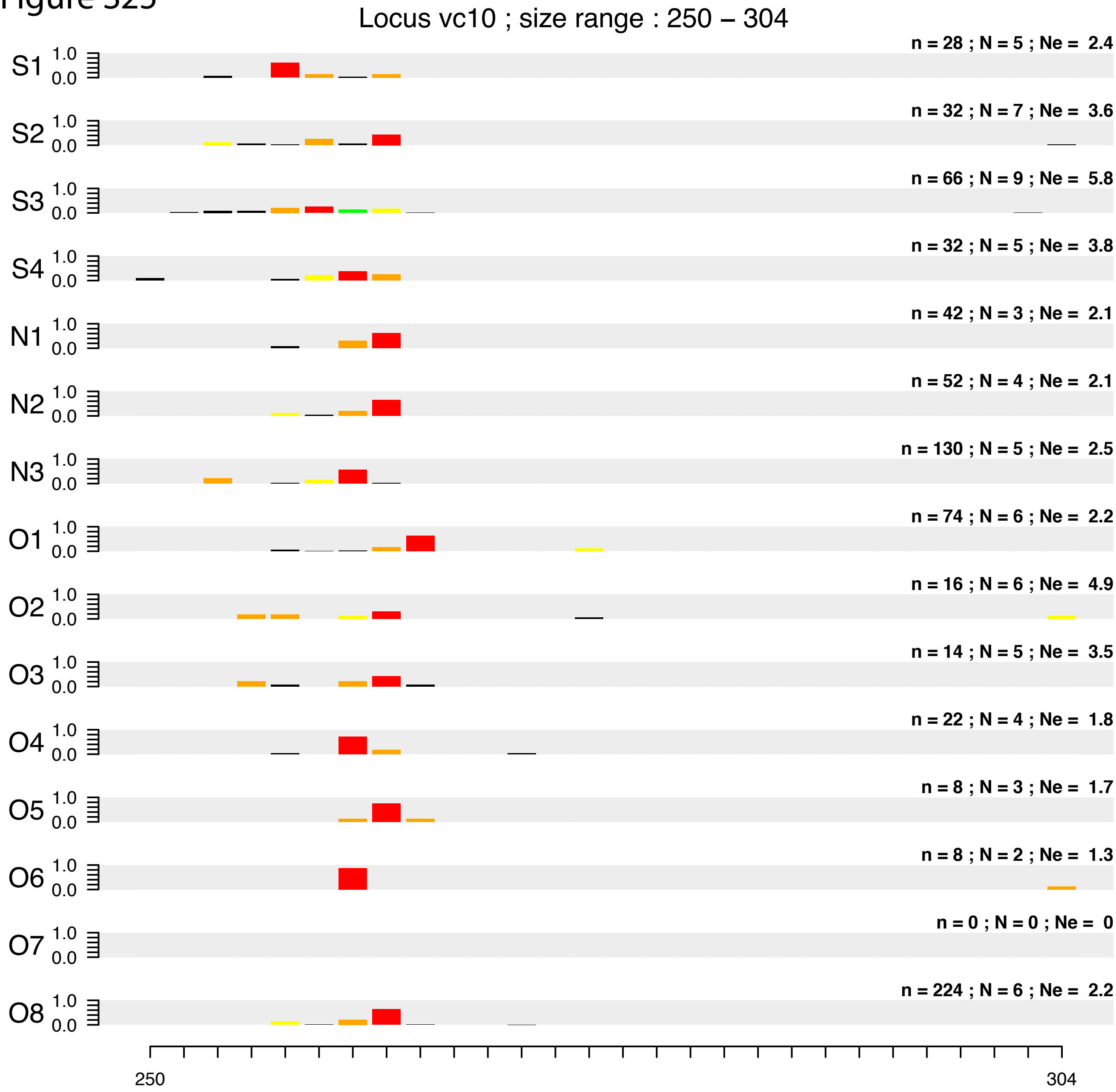

Locus vc10 ; size range : 250 – 304
S1
n = 28 ; N = 5 ; Ne = 2.4
S2
n = 32 ; N = 7 ; Ne = 3.6
S3
n = 66 ; N = 9 ; Ne = 5.8
S4
n = 32 ; N = 5 ; Ne = 3.8
N1
n = 42 ; N = 3 ; Ne = 2.1
N2
n = 52 ; N = 4 ; Ne = 2.1
N3
n = 130 ; N = 5 ; Ne = 2.5
O1
n = 74 ; N = 6 ; Ne = 2.2
O2
n = 16 ; N = 6 ; Ne = 4.9
O3
n = 14 ; N = 5 ; Ne = 3.5
O4
n = 22 ; N = 4 ; Ne = 1.8
O5
n = 8 ; N = 3 ; Ne = 1.7
O6
n = 8 ; N = 2 ; Ne = 1.3
O7
n = 0 ; N = 0 ; Ne = 0
O8
n = 224 ; N = 6 ; Ne = 2.2
1.0
0.0
250
304

Figure S26

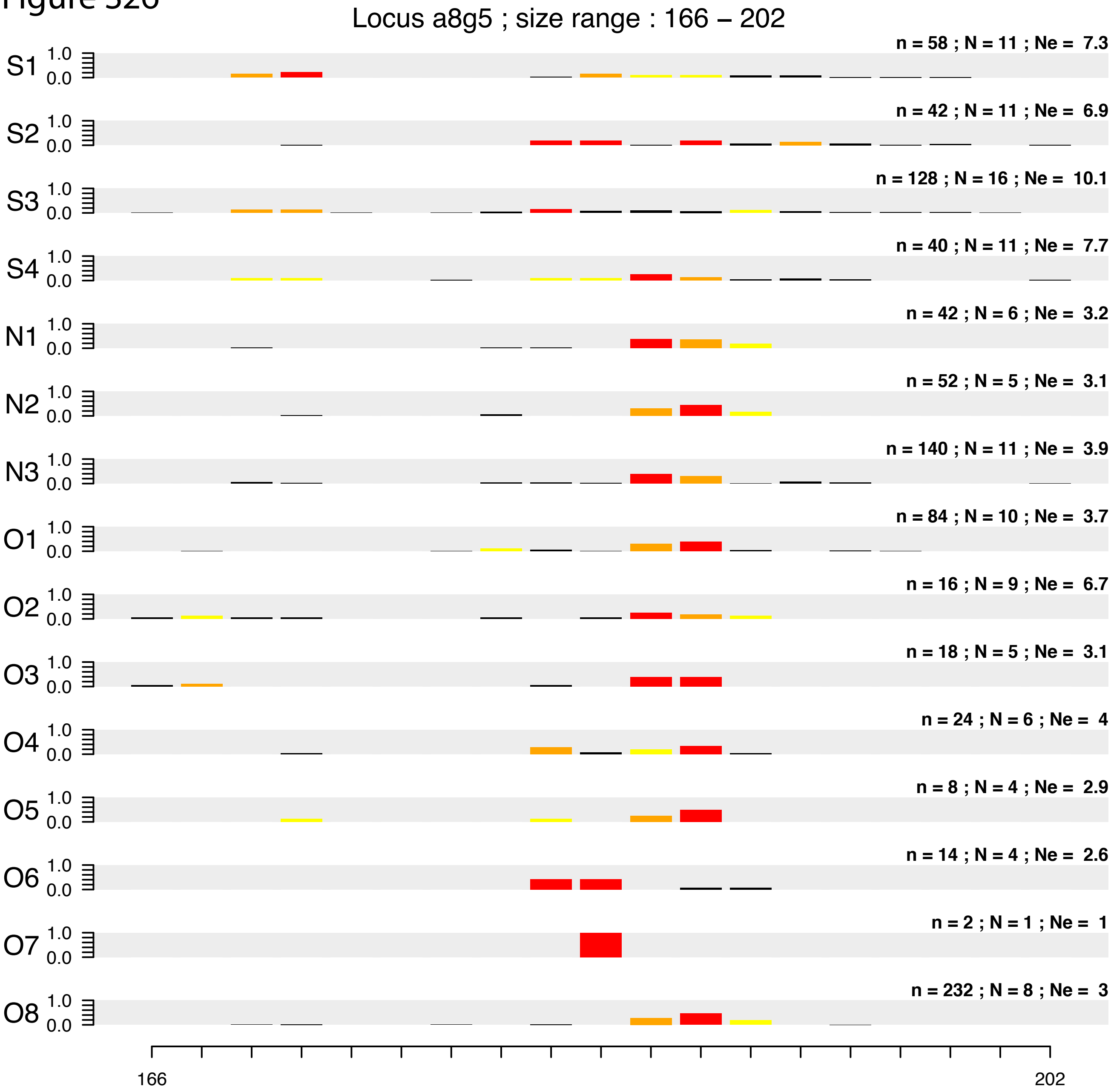
Locus a8g5 ; size range : 166 – 202
S1
n = 58 ; N = 11 ; Ne = 7.3
S2
n = 42 ; N = 11 ; Ne = 6.9
S3
n = 128 ; N = 16 ; Ne = 10.1
S4
n = 40 ; N = 11 ; Ne = 7.7
N1
n = 42 ; N = 6 ; Ne = 3.2
N2
n = 52 ; N = 5 ; Ne = 3.1
N3
n = 140 ; N = 11 ; Ne = 3.9
O1
n = 84 ; N = 10 ; Ne = 3.7
O2
n = 16 ; N = 9 ; Ne = 6.7
O3
n = 18 ; N = 5 ; Ne = 3.1
O4
n = 24 ; N = 6 ; Ne = 4
O5
n = 8 ; N = 4 ; Ne = 2.9
O6
n = 14 ; N = 4 ; Ne = 2.6
O7
n = 2 ; N = 1 ; Ne = 1
O8
n = 232 ; N = 8 ; Ne = 3
1.0
0.0
166
202
