# Supplementary material for: Evidence for late Pleistocene origin of Astyanax mexicanus cavefish
Source: BMC Evol Biol. 2018 Apr 18;18:43. doi: 10.1186/s12862-018-1156-7 (PMC5905186; doi:10.1186/s12862-018-1156-7)
Supplement: Supplementary file 4 — Rationale of dating with pool-seq SNPs. (PDF 420 kb) [file 12862_2018_1156_MOESM4_ESM.pdf]

Additional Information for

Evidence of late Pleistocene origin of *Astyanax mexicanus* cavefish

Julien Fumey[1,2], Hélène Hinaux[3], Céline Noirot[4], Claude Thermes[2], Sylvie Rétaux[3] and Didier Casane[1,5,*]

[1] Évolution, Génomes, Comportement, Écologie. CNRS, IRD, Univ Paris-Sud. Université Paris-Saclay. F-91198 Gif-sur-Yvette, France.

[2] Institute for Integrative Biology of the Cell (I2BC), CEA, CNRS, Université Paris-Sud, UMR 9198, FRC 3115, Avenue de la Terrasse, Bâtiment 24, Gif-sur-Yvette, Paris F-91198, France.

[3] DECA group, Paris-Saclay Institute of Neuroscience, UMR 9197, CNRS, Gif sur Yvette, France.

[4] Plateforme Bioinformatique Toulouse, Midi-Pyrénées, UBIA, INRA, Auzeville Castanet-Tolosan, France

[5] Université Paris Diderot, Sorbonne Paris Cité, France.

* Corresponding author:

Didier Casane

Laboratoire Évolution, Génomes, Comportement, Écologie, UMR 9191 CNRS, 1 avenue de la Terrasse, 91198 Gif sur Yvette, France.

Tel: +33169823759

Email: Didier.Casane@egce.cnrs-gif.fr

This PDF file includes:

1 Detailed method

1.1 Rationale of the method

1.2 Summary statistics

1.3 Pool-seq and estimation of summary statistics

1.4 Evaluation of the method

1 Detailed method

1.1 Rationale of the method

To illustrate how the pattern of polymorphism and substitutions rate vary within and between two isolated populations before mutations/drift equilibrium is established, we ran a simulation with two populations, one with a population size equal to 5,000 and another with a population size equal to 10,000 (**Figure S1A**). We can see that, over one million years, the derived alleles are fixed at the same rate in both populations (**Figure S1B**). However, if we focus on the first one hundred thousand years after the split of the ancestral population, more derived alleles are fixed in the small population (**Figures S1C and S1D**). This is the consequence of a higher rate of fixation of standing derived alleles in the small population (**Figure S1E**). *De novo* mutations also arise in both populations and start fixing at the same rate, but with a delay in the larger population (**Figure S1F**). The polymorphism in the larger population is stable because it is a continuum of the ancestral population that was in mutation/drift equilibrium, whereas the polymorphism in the smaller population reaches a new equilibrium value after a few thousand years which is, as expected, half the value observed in the population that is two times larger (**Figure S1G**). The shared polymorphism decreases quickly (**Figure S1H**). Thus, an excess of derived alleles fixed in a small population could be a signature of it recent origin (**Figure S1C**). We defined summary statistics (see below for details) that describe: 1) differences in substitution rates, 2) population specific and shared polymorphism. Using simulations of the evolution of two closely related populations of different sizes and allowing gene flow, we looked for the set of parameters, *i.e.* population sizes, migration rates and cavefish population age that could best explain the observed summary statistics.

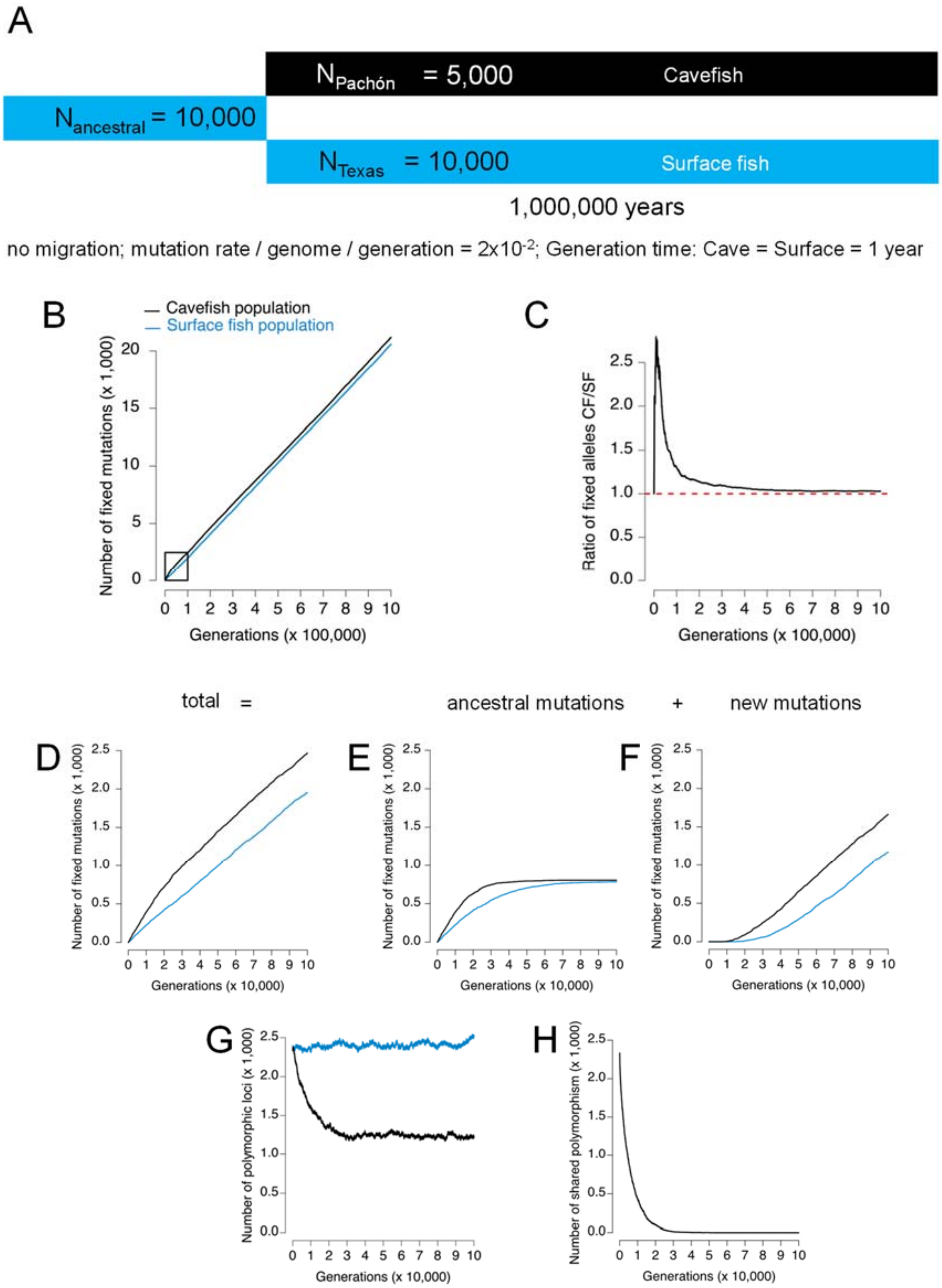

Figure S1: A simulation to illustrate a simple explanation of an excess of neutral substitutions in a small cavefish population. (A) evolutionary model; (B) numbers of substitutions over 10^6 generations; (C) CF/SF substitution ratio; (D) the first 10^5 generations; (E) fixation of mutations already present in the ancestral population; (D) fixation of new mutations that appeared after the separation of the populations; (G) number of polymorphic sites; (H) number of shared polymorphic sites.

1.2 Summary statistics

We estimated the relative frequency of eight classes of SNPs described below (**Figure S2**) in order to evaluate: 1) the divergence of cavefish (CF) and surface fish (SF), 2) lineage specific and shared polymorphism.

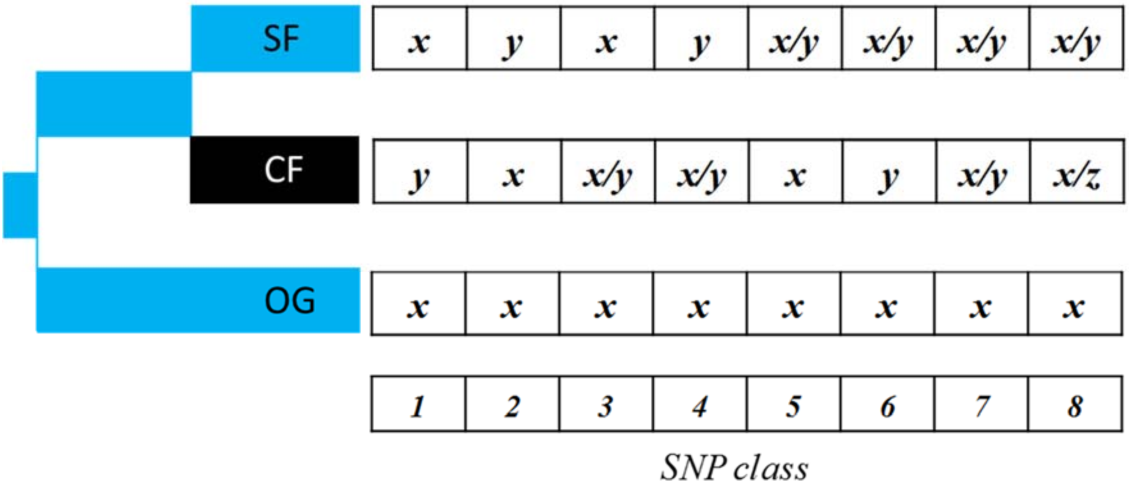

Figure S2. Analysis of polymorphisms in *Astyanax mexicanus*. Texas surface (SF) *vs* Pachón cave (CF) using *Hyphessobrycon anisitsi* as an outgroup (OG). The eight SNP classes identified from all possible polymorphism patterns within and between two populations. Class 1: Different alleles fixed in each population, derived allele in cavefish; Class 2: Different alleles fixed in each population. derived allele in surface fish; Class 3: Polymorphism in cavefish, ancestral allele fixed in surface fish; Class 4: Polymorphism in cavefish, derived allele fixed in surface fish; Class 5: Polymorphism in surface fish. ancestral allele fixed in cavefish; Class 6: Polymorphism in surface fish. derived allele fixed in cavefish; Class 7: Shared polymorphism; Class 8: Divergent polymorphism. x. y and z can be one of the four nucleotides A, T, G, C.

1.3 Pool-seq and robustness of the estimations of summary statistics

In order to estimate the summary statistics defined above, we apply a pool-seq approach [1]. We sequenced RNA extracted from pooled embryos. The accuracy of this approach was evaluated by simulations of the sampling process. We simulated the evolution of two populations and alleles frequencies in each populations and the summary statistics were calculated. Then we pick up *n* fish in each population. The genotype of a fish at a polymorphic locus was generated according to the frequencies of the alleles at that locus in its population. For each locus, each fish produced *m* RNA sequences, *m* being a random number drawn from a uniform distribution between 0 and x - we also examine the case in

which there is one read and only one read (only one allele is sequenced) for each fish, *i.e.* no variation of the number of reads/fish. This way we simulated the possibility that the contribution of the different fish in the total number of reads could have been very heterogeneous. We found that if we picked up more than 10 fish, and even if there was a very heterogeneous contribution of each fish to the pool of reads (between 0 and 1000), this is sufficient to obtain accurate estimations of the summary statistics (with 20 fish the estimations are very close to the expected values)(**Figures S3A and S3B**), even if the estimations of the allele frequencies at each locus were not accurate (**Figure S3C**).

A

SNP class	Simulation	10 / 0:1000	20 / 0:1000
1	0.13	0.14	0.13
2	0.07	0.09	0.08
3	0.11	0.11	0.11
4	0.03	0.03	0.03
5	0.47	0.46	0.47
6	0.10	0.10	0.10
7	0.09	0.07	0.07
Score		0.01	0.00

$$Score = \sum_{i=1}^{7} \frac{(o_i - m_i)^2}{m_i}$$

o_i: frequency of SNPs in the class *i in the simulation*
m_i: frequency of SNPs in the class *i* when RNA pooled-seq was simulated

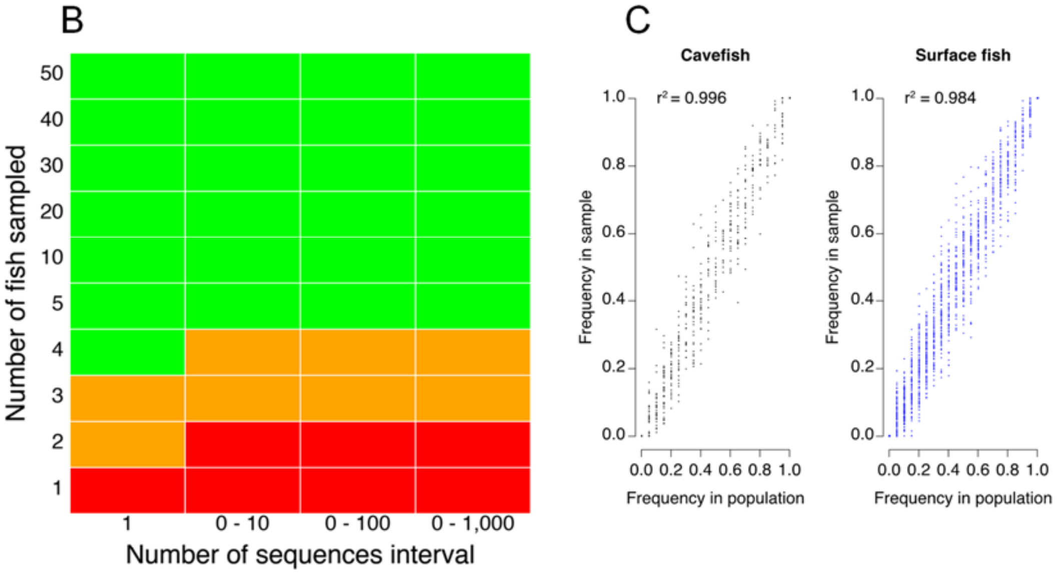

Figure S3. Pool-seq and robustness of the estimations of summary statistics. (A) comparison of the true summary statics using the SNPs found in two simulated populations with the summary statistics estimated after sampling 10 or 20 fish (the number of reads for each fish was a random number between 0 and 1,000). (B) Heatmap of the score according to the number of fish sample in the population and the number of reads per fish which is a random number between 0 and m (m = maximum number of reads). Green: Score < 3; orange: 3 < Score < 15; red: Score > 15. (C) comparison of derived allele frequencies in each population with their estimations with a sample of 20 fish (each fish contributing for m reads ($0 \leq m \leq 1{,}000$).

Reference

1. Schlotterer C, Tobler R, Kofler R, Nolte V: **Sequencing pools of individuals - mining genome-wide polymorphism data without big funding**. *Nat Rev Genet* 2014, **15**(11):749-763.
